# Practical guidelines for rigor and reproducibility in preclinical and clinical studies on cardioprotection

**DOI:** 10.1007/s00395-018-0696-8

**Published:** 2018-08-17

**Authors:** Hans Erik Bøtker, Derek Hausenloy, Ioanna Andreadou, Salvatore Antonucci, Kerstin Boengler, Sean M. Davidson, Soni Deshwal, Yvan Devaux, Fabio Di Lisa, Moises Di Sante, Panagiotis Efentakis, Saveria Femminò, David García-Dorado, Zoltán Giricz, Borja Ibanez, Efstathios Iliodromitis, Nina Kaludercic, Petra Kleinbongard, Markus Neuhäuser, Michel Ovize, Pasquale Pagliaro, Michael Rahbek-Schmidt, Marisol Ruiz-Meana, Klaus-Dieter Schlüter, Rainer Schulz, Andreas Skyschally, Catherine Wilder, Derek M. Yellon, Peter Ferdinandy, Gerd Heusch

**Affiliations:** 1 Department of Cardiology, Aarhus University Hospital, Palle-Juul Jensens Boulevard 99, 8200 Aarhus N, Denmark; 2 The Hatter Cardiovascular Institute, University College London, 67 Chenies Mews, London, WC1E 6HX UK; 3 The National Institute of Health Research, University College London Hospitals Biomedial Research Centre, Research and Development, London, UK; 4 National Heart Research Institute Singapore, National Heart Centre, Singapore, Singapore; 5 Yon Loo Lin School of Medicine, National University Singapore, Singapore, Singapore; 6 Cardiovascular and Metabolic Disorders Program, Duke-National University of Singapore, 8 College Road, Singapore, 169857 Singapore; 7 Laboratory of Pharmacology, Faculty of Pharmacy, National and Kapodistrian University of Athens, Athens, Greece; 8 Department of Biomedical Sciences, CNR Institute of Neuroscience, University of Padova, Via Ugo Bassi 58/B, 35121 Padua, Italy; 9 Institute for Physiology, Justus-Liebig University Giessen, Giessen, Germany; 10 Cardiovascular Research Unit, Luxembourg Institute of Health, Strassen, Luxembourg; 11 Department of Clinical and Biological Sciences, University of Torino, Turin, Italy; 12 Experimental Cardiology, Vall d’Hebron Institut de Recerca (VHIR), Hospital Universitari Vall d’Hebron, Pg. Vall d’Hebron 119-129, 08035 Barcelona, Spain; 13 Department of Pharmacology and Pharmacotherapy, Semmelweis University, Budapest, Hungary; 14 Pharmahungary Group, Szeged, Hungary; 15 Centro Nacional de Investigaciones Cardiovasculares Carlos III (CNIC), IIS-Fundación Jiménez Díaz, CIBERCV, Madrid, Spain; 16 Second Department of Cardiology, Faculty of Medicine, Attikon University Hospital, National and Kapodistrian University of Athens, Athens, Greece; 17 Institute for Pathophysiology, West German Heart and Vascular Center, University of Essen Medical School, Essen, Germany; 18 Department of Mathematics and Technology, Koblenz University of Applied Science, Remagen, Germany; 19 Institute for Medical Informatics, Biometry, and Epidemiology, University Hospital Essen, Essen, Germany; 20 Explorations Fonctionnelles Cardiovasculaires, Hôpital Louis Pradel, Lyon, France; 21 UMR, 1060 (CarMeN), Université Claude Bernard, Lyon1, Villeurbanne, France

## Introduction

The potential for ischemic preconditioning to reduce infarct size was first recognized more than 30 years ago [180]. Despite extension of the concept to ischemic postconditioning [460] and remote ischemic conditioning [202, 344] and literally thousands of experimental studies in various species and models which identified a multitude of signaling steps [199], so far there is only a single and very recent study, which has unequivocally translated cardioprotection to improved clinical outcome as the primary endpoint in patients [155, 200]. Many potential reasons for this disappointing lack of clinical translation of cardioprotection have been proposed, including lack of rigor and reproducibility in preclinical studies [54, 195], and poor design and conduct of clinical trials [196, 206]. There is, however, universal agreement that robust preclinical data are a mandatory prerequisite to initiate a meaningful clinical trial. In this context, it is disconcerting that the CAESAR consortium (Consortium for preclinicAl assESsment of cARdioprotective therapies) in a highly standardized multi-center approach of preclinical studies identified only ischemic preconditioning, but not nitrite or sildenafil, when given as adjunct to reperfusion, to reduce infarct size [230]. However, ischemic preconditioning—due to its very nature—can only be used in elective interventions, and not in acute myocardial infarction [181, 197, 203]. Therefore, better strategies to identify robust and reproducible strategies of cardioprotection, which can subsequently be tested in clinical trials must be developed [184]. We refer to the recent guidelines for experimental models of myocardial ischemia and infarction [279], and aim to provide now practical guidelines to ensure rigor and reproducibility in preclinical and clinical studies on cardioprotection. In line with the above guidelines [279], we define rigor as standardized state-of-the-art design, conduct and reporting of a study, which is then a prerequisite for reproducibility, i.e. replication of results by another laboratory when performing exactly the same experiment.

## Randomization, blinding, power analysis and statistics

### Study design, randomization, blinding, power analysis, statistics

The ICH (International Conference on Harmonization) E9 guideline [168] is the most prominent guideline for statistical principles in clinical trials. In some analogy, the ARRIVE (Animal Research: Reporting of In vivo Experiments) guidelines make recommendations for reporting animal research [94, 212, 249], notably study design, including power analysis and sample size planning, randomization of study groups, blinding of investigators, and adequate statistical procedures to evaluate and interpret the data [345]. Some journals [54, 176] have established additional guidelines for planning, performing and reporting experimental studies and the respective data. For clinical trials, the ICH E6 guideline for Good Clinical Practice (see http://www.ich.org/fileadmin/Public_Web_Site/ICH_Products/Guidelines/Efficacy/E6/E6_R2__Step_4_2016_1109.pdf) must be considered. This guideline addresses various issues related to quality control, including monitoring before, during, and after the trial. In addition, there are clinical trial registries to provide better transparency of and access to clinical trials. Similar registries would also be useful for preclinical trials.

Here, we aim to provide a pragmatic framework for study design, including power analysis, randomization, blinding and statistical data analysis for the presentation of preclinical and clinical studies (for clinical trials see also http://www.consort-statement.org/) on acute myocardial infarction and cardioprotection.

### Exploratory vs. prospective studies

The gold standard for clinical trial design is a prospective, randomized, blinded, controlled study. The bias of such prospective studies is lowest, and their data are most robust. However, most studies on cardioprotection fall into the category of observational/exploratory study; other studies retrospectively analyze previously recorded data. The selection of the design (observational/exploratory vs. prospective) for a given project always represents a compromise of a most rigorous approach vs. limited resources and feasibility. Clearly, an exploratory study, while aiming for novelty, is associated with a greater risk of false-positive findings due to confounding factors, whereas a prospective study aims for an existing specific hypothesis but provides more robust data.

### Study design

#### Selection of variables/endpoints

According to the ICH E9 guideline [168], the primary variable “should be the variable capable of providing the most relevant and convincing evidence directly related to the primary objective of the trial”. This is also true for experimental studies, and ideally there is only one primary endpoint. Infarct size is the gold standard primary endpoint; ventricular function or release of biomarkers during reperfusion may also be used as endpoints of cardioprotection.

When infarct size is used as the primary endpoint, secondary endpoints can be hemodynamics such as coronary blood flow or ventricular function. Further, secondary endpoints can be mitochondrial function or expression or activation of signaling proteins. Material from biopsies is often analyzed at only one time point, mostly at the end of the experiment.

#### Number of experimental groups

The simplest design of a cardioprotection study has only two groups (control/placebo with myocardial infarction without intervention versus the verum group with myocardial infarction and with cardioprotective intervention). This basic model can be extended to other groups, e.g. myocardial infarction with cardioprotective intervention and blocking agents or another cardioprotective intervention, etc. Clearly, the choice and, therefore, the number of groups depend on the aim of the experiment. The primary endpoint must be measured in all groups. When there are more than two groups and/or more than one endpoint, one has to carry out more than one statistical test. As a consequence, the multiplicity of tests must be considered to control for the rate of false-positive conclusions at an acceptable level. When a significant difference in at least one endpoint is sufficient to claim an effect, the significance level *α* can be adjusted using the Bonferroni correction, where *P* values are compared with *α*/*k* instead of α when performing *k* statistical tests. For instance, if *α* = 0.05 (as usual) and there are four endpoints, a *P* value has to be smaller than 0.05/4 = 0.0125 for achieving significance. The Bonferroni correction is the easiest adjustment; there are more sophisticated and more efficient alternatives [319]. When the decision rule is that statistical significance is needed for all primary variables, no special method such as the Bonferroni adjustment to consider for the multiplicity of tests is needed. All tests can be performed with the unadjusted *α*-level, but all single *P* values have to be ≤ *α* for significance. This procedure inflates the type II error rate, which must be taken into account for sample size determination.

#### Inclusion and exclusion criteria

Inclusion and exclusion criteria must be specified in advance and reported as transparently and as detailed as possible [54, 94, 176, 212, 249]. Individual animals from one breeder with comparable baseline characteristics (age, gender, body weight, etc.) should be included. When genetic variants are analyzed in one experimental group, their wild-type littermates or animals with a comparable genetic background must serve as controls. All factors aside from the intervention (age, sex and housing conditions of the animals, but also the individual investigator), should be kept unchanged. Major deviations, as defined in advance, must result in exclusion from analysis in experimental studies. Exclusion is particularly mandatory for factors known to impact on the intervention itself (e.g. use of blocking agent from different batches, different procedures and algorithms of the protective intervention, etc.) and for factors known to impact on the endpoints (e.g. temperature, etc.). However, in prospective preclinical and clinical trials all randomized preparations/individuals (patients/animals) must be included in the intention-to-treat analysis. An additional per-protocol analysis excluding preparations/animals/patients who seriously violated the protocol (see ICH E9 guideline [168]) is frequently of scientific relevance. A similar procedure with two separate analyses is also possible when potentially interfering variables become apparent only in retrospect: one may perform the data analysis twice, once with all data points and in addition once after exclusion of the respective data points. In prospective experimental studies, however, it is common and also accepted [94, 212, 249] that exclusion criteria are reported in advance, excluded experiments are reported, but the analysis as such is performed only on per-protocol experiments.

#### Effect size and sample size

Most often, the investigator has an approximate idea of the expected infarct size in the control/placebo group from prior data. Using such data [e.g. infarct size is 50 ± 15% of area at risk (mean ± standard deviation)], the sample size can be calculated when specifying the effect size. At an expected infarct size reduction from 50 to 35 and an expected standard deviation of 15, the effect size is (50 – 35)/15 = 1. In a two-sided *t* test (with a significance level *α* = 0.05), 23 animals/group are necessary for a power of 90%. With 17 animals/group, the power is 80%.

A study should only be started if the required sample size is in fact achievable. When the required sample size is unattainably high, other endpoints with less variability or changes in study design must be considered. For instance, if a paired design is possible, a pre-specified power can be achieved with fewer observations. However, the power should be at least 80% (see ICH E9 guideline [168]), and we recommend a larger power of 90% or even more. There are various software tools for sample size calculation, for example, the free software *R* has several functions for sample size calculation.

#### Randomization

Online and free-of-charge tools for randomization such as the *R* package randomize *R*, the Research Randomizer available at www.randomizer.org, or the Random Allocation Software proposed by Saghaei [361] should be used to randomize to treatment groups. For preparation of the randomization, the total sample size is needed when generating the random list for the entire study. In clinical trials, a block randomization is commonly used to reduce bias and achieve balance in the allocation of participants to treatment arms, especially when the sample size is small [120]. Whenever possible, blinding is recommended. When blinding is not possible or can be compromised by the effects of the intervention (e.g. when a cardioprotective intervention is apparently performed or induces clearly visible responses), this fact must be reported. Unblinding of the analyzing investigators and other staff should be avoided until all data of all experiments are obtained. Any interim analysis is only permitted if planned in advance. However, such interim analysis is an additional analysis, which again results in a multiplicity of statistical tests. When the analysis of the experiment results in no or only a borderline significance, it is not permitted to change the sample size post hoc, to add a new randomized group, or to supplement additional experiments.

When, for whatever reason, the required sample size cannot be achieved and the study has to be terminated, data can be analyzed, but the statistical power is obviously low. The premature study termination and reasons for it must be reported.

### Data analysis

All endpoints must be evaluated in a blinded fashion and/or by investigators not involved in the actual experiment. Endpoints should be assessed as objectively as possible and, if possible, automatically using a predefined algorithm, especially when blinding is not possible. Quantification of infarct size by histology and/or software-assisted planimetry must follow standardized protocols in demarcation of infarcted areas from non-infarcted areas, camera/microscope settings and image processing.

#### Parametric vs. non-parametric statistics

The statistical test(s) for the primary endpoint(s) must be selected before the experiment is conducted. Additional statistical analyses can be performed as sensitivity analyses to investigate the robustness of the results. Non-parametric tests are “distribution-free” and, as such, can also be used for non-normally distributed variables. In contrast, parametric tests are those that make assumptions such as that obtained data are normally distributed. If data are a priori expected to deviate from normality, nonparametric tests can be used. Examples are the Wilcoxon–Mann–Whitney and the Kruskal–Wallis tests which are analogs to the Student *t*- and analysis of variance (ANOVA) *F* tests.

In some applications, pretests are performed to test data for normality or to test for homogeneity of variances. Those pretests cannot be recommended, since the failure to reject the null hypothesis does not imply that the null hypothesis holds, in particular since the power of the pretests is often low. Furthermore, preliminary tests have their own assumptions and it would be inconsistent not to check these assumptions.

A transformation, such as the logarithmic transformation, can be useful to, e.g. normalize the data. However, transformations should be determined in advance based on previous experimental or scientific evidence.

#### Missing values and outliers

Missing values can bias the results. In the presence of many missing values, the ability to draw valid conclusions is seriously compromised. Unfortunately, there is “no methodological approach for handling missing values that is universally accepted in all situations” [1]. There are imputation methods to replace missing values. When the primary endpoint is repeatedly measured (e.g. infarct size calculated from release of biomarkers during the experiment), the last observation carried forward (LOCF) method can be used, i.e. missing values of subsequent time points are replaced by previous measurements. The LOCF method is considered a conservative method when the difference between groups increases with time in placebo-controlled studies. Statistical programs such as SAS or *R* provide easy access to the LOCF method. If no imputation method is applied, missing values in the primary endpoint result in the exclusion of the patient/animal/preparation from further analysis of the primary endpoint.

**Fig. 1:**
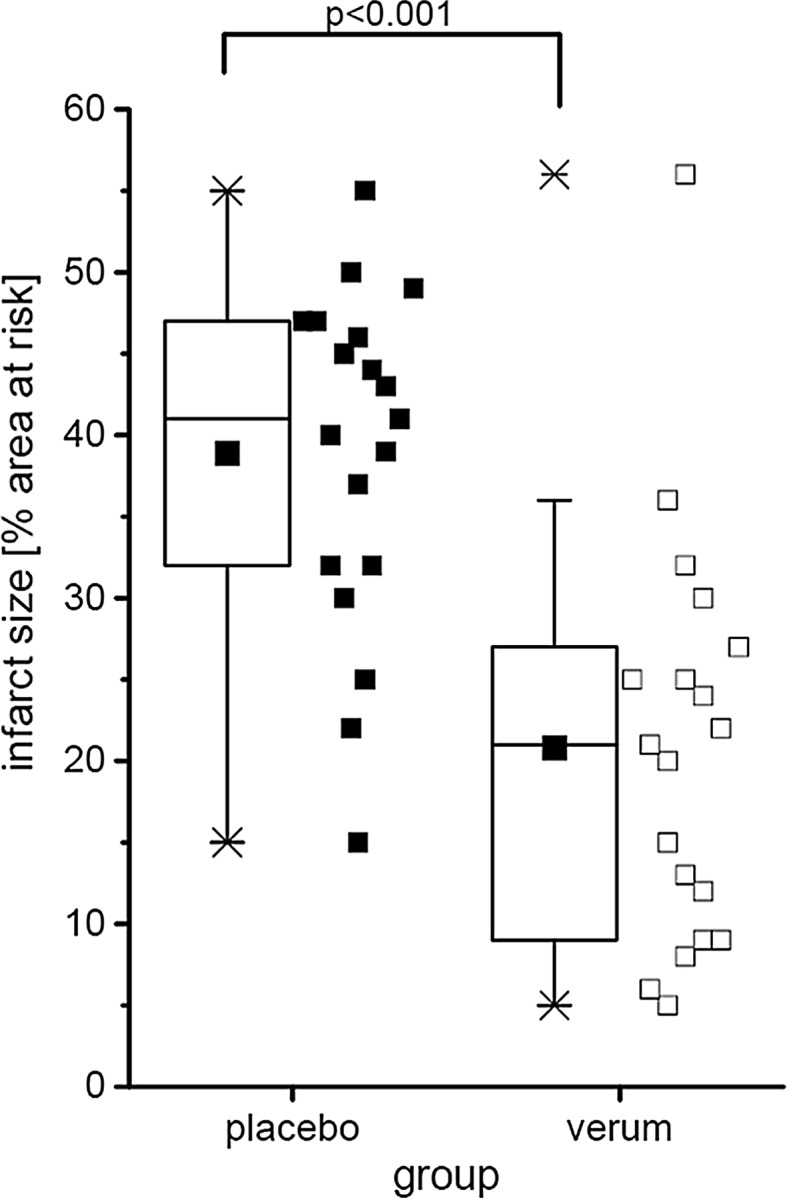
Virtually created data on infarct size as percent of area at risk. For two groups, placebo vs. verum, data are presented as minimum and maximum (crosses), interquartile range from 25 to 75% (box), mean (square), and median (line) in a box plot. The maximum value of the verum group is identified as outlier. All single values (squares) are depicted. *P* value as the result of a two-sided Student *t* test

A variety of tests may identify outliers (e.g. the Dean and Dixon test). Graphical methods for the identification of outliers are recommended. Box plots (Fig. 1) are useful for quantitative data whether normally distributed or not, and the identification of outliers is easy. The “whiskers” of the full box plot according to Tukey’s [428] original definition extend to the most extreme data within the inner fences, that is, first quartile—1.5 inter-quartile range and third quartile + 1.5 inter-quartile range. Data outside the inner fences, i.e. data points outside of the range depicted by the box and whiskers, are considered outliers. Except for the intention-to-treat analysis of clinical trials, outliers can be deleted before statistical analysis if there is a rational, context-related reason or if the observed value is theoretically not possible. However, when outliers are identified, one analysis each with and without outliers is recommended, and the procedure of outlier identification must be reported. There are also statistical methods which are robust with regard to outliers such as nonparametric methods based on ranks.

#### Data presentation and statistical analysis

Data presentation and statistical analysis must be specified [54, 176, 249]. For normally distributed data, it is appropriate to present means and standard deviations. However, presentation of all single values and a box plot with means, medians and quartiles visualizes the data in more detail (Fig. 1) [428].

For each statistical test, the value of the test statistic, the degree(s) of freedom if appropriate, the estimates of effect sizes with their standard deviation or standard error, and the accurate *P* value (when the *P* value is lower than 0.001 just mention *P* < 0.001) must be reported. Whenever a *P* value is given, in particular for important comparisons, the 95% confidence intervals are provided, if available. Caveat—different statistical software packages work with slightly different assumptions and can come to different outcomes, as demonstrated for the Wilcoxon–Mann–Whitney [38]. An example for infarct size data presentation is given in Fig. 1. Infarct size of a placebo and a verum group is presented: data are presented as box plot and single data points, and a two-sided Student *t* test was used for comparison, there is one outlier in the verum group. When the analysis is performed with all data including the outlier, the test statistic is *t* = 4.82 (36 degrees of freedom) and the *P* value is given as *P* < 0.001. As an effect size we can consider the difference between the group means. This difference is 38.89–20.79 = 18.11 with a standard deviation of 11.58. The 95% confidence interval for this difference ranges from 10.48 to 25.73. When the analysis is performed without the outlier, the test statistic is *t* = 6.08 (35 degrees of freedom), the *P* value is given as *P* < 0.001, and the difference between the group means is 38.89–18.83 = 20.06 with a standard deviation of 10.03. The 95% confidence interval for this difference ranges from 13.36 to 26.76.

When more than two groups are compared (e.g. placebo, verum ± blocking agents), a one-way ANOVA is appropriate to compare the different groups. When a significant difference is detected in the ANOVA analysis, pairwise comparisons of the individual groups can follow. Again, the multiplicity of tests must be considered, and the Bonferroni adjustment or another more sophisticated method can be applied. Statistical software such as *R* or SAS offers various methods to adjust for multiple comparisons; in any case, the respective *P* values must be given.

### Use of statistical analyses in experimental and clinical cardioprotection studies

When comparing just a single endpoint at one single time point, a Student *t* test is appropriate for comparison of two groups; for more than two groups, a one-way ANOVA with subsequent tests for pairwise comparisons of mean values is appropriate. This statistical analysis is appropriate for viability assessments at the end of a hypoxia/reoxygenation protocol in isolated cardiomyocytes or for infarct size assessments in isolated perfused heart and in vivo experiments.

**Fig. 2:**
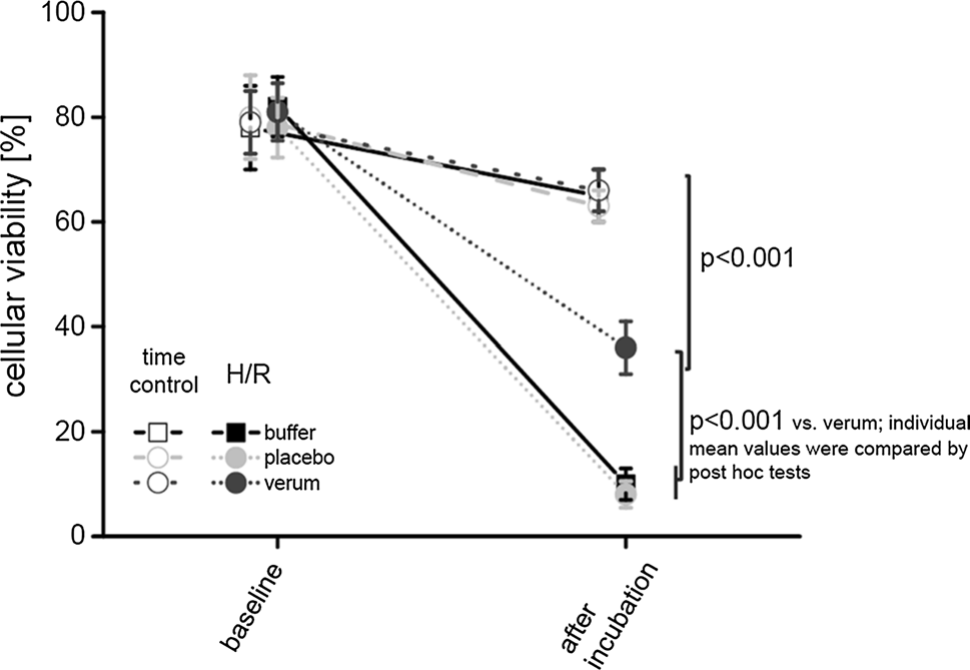
Virtually created data on cellular viability. Sequential data are analyzed throughout the time course (baseline and after incubation) of the different experimental groups (time control and hypoxia/reoxygenation (H/R) with buffer, placebo, and verum, respectively). Data are presented as mean ± standard deviation. *P* values as the result of two-way repeated-measures ANOVA (group, time) with subsequent post hoc tests are reported

**Fig. 3:**
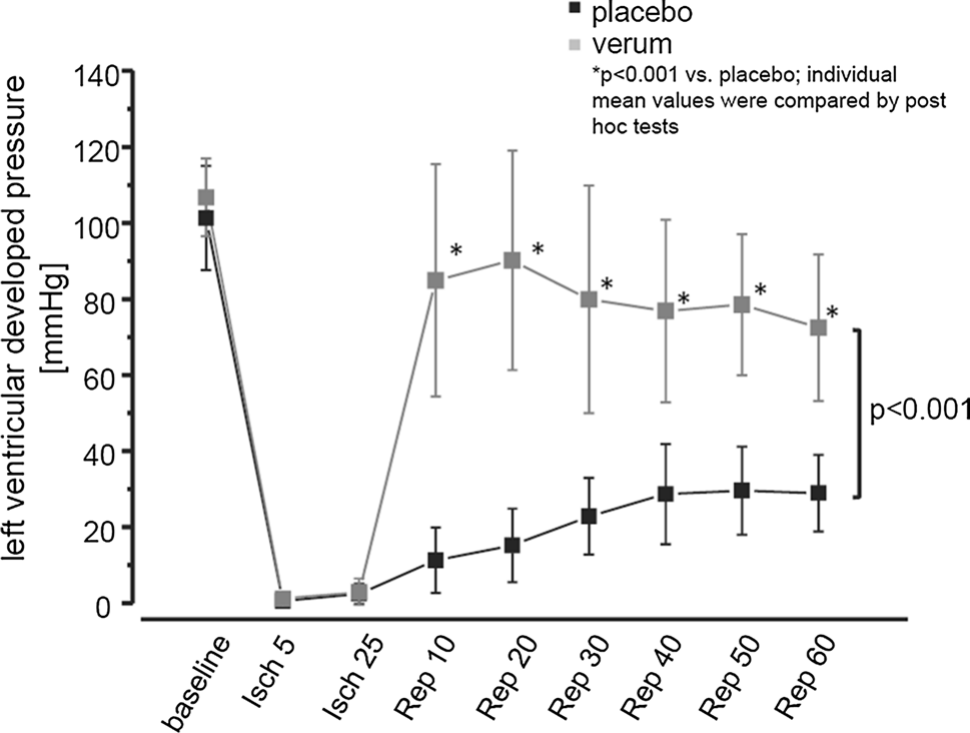
Virtually created data on left ventricular developed pressure during ischemia (Isch) and reperfusion (Rep). For two groups, placebo vs. verum, data are presented as mean ± standard deviation. *P* values as the result of two-way repeated-measures ANOVA (group, time) with subsequent post hoc tests are reported

When sequential data points are analyzed throughout the time course of the experiment, such as viability from baseline to reoxygenation in isolated cardiomyocytes or hemodynamics in vitro or in vivo or the results of multiple Western blots, a two-way repeated-measures ANOVA (group, time) is appropriate (Figs. 2, 3). Again, the Bonferroni or another adjustment must be made for multiple testing. However, to reduce the risk of false positive results, sample size and variance homogeneity must be taken into account [286].

To assess the outcome of infarction and cardioprotection interventions, survival analyses are used in animal experiments and in clinical trials. For such survival time and other time-to-event endpoints [as combined endpoints, e.g. major adverse cardiac events or major adverse cardiac and cerebral events (MACCE)], statistical methods such as the logrank test or Cox’s proportional hazards regression are used. Sample size and power calculations are then based on an assumed hazard ratio, which is based on the expected difference between groups. For example, the hazard ratio is 0.2 when 90% of the animals in one group and 59% of animals in the other group are still alive at the same time (assuming a constant hazard rate). Then, for the logrank test, 47 individuals/group are needed for a power of 90% (*α* = 0.05, two-sided).

### Specific guidance for power calculations and statistics in clinical myocardial infarction trials

Appropriate power calculation depends on the choice of endpoint, which is myocardial salvage in proof-of-concept trials or a combination of cardiac death and hospitalization for heart failure in clinical outcome trials. Using imaging modalities for the quantification of myocardial salvage, previous studies of primary percutaneous coronary intervention (PPCI) with stenting have demonstrated an area at risk within the magnitude of 30% of the left ventricle and a salvage index of 0.50 [243, 370]. A final infarct size of ≤ 12% of left ventricle is a rational target, because an infarct size of this magnitude is associated with very low mortality [63, 306]. A 20% reduction in final infarct size from 15% to 12% corresponds to an increase in salvage index from 0.5 [(30 − 15)/30] to 0.6 [(30 − 12)/30]. Assuming a standard deviation of the salvage index of 0.23 [370] and that data conform with a parametric distribution, a demonstration of an increase in salvage index of 0.1 requires a sample size of 83 patients in each group with a power of 80% and a two-sided *α* value of 0.05. With a 5% drop-out rate, a cohort size of 176 is required. For a power of 90%, 113 patients/group are required, resulting in a total of 238 patients after adjusting for a drop-out rate of 5%. From a review of means and standard deviations of infarct size in CMR studies on cardioprotection in ST segment elevation myocardial infarction (STEMI) patients, Bulluck et al. here provided a table to estimate sample size, based on patient selection criteria and expected effect size [62].

Using a primary combined endpoint of cardiac death and heart failure hospitalization at 12 months for clinical outcome, studies will require a number of patients in the order of magnitude of 5200 patients, when using a *χ*^2^ test. Current cardioprotection studies in patients undergoing reperfusion for STEMI are challenged by the excellent outcome with modern standard therapy. Cardiac mortality at 12 months has decreased continuously over the last 25 years [368] and is now below 6% [126], while rehospitalisation for heart failure is 8% [126]. Our most recent observations in patients included in the CONDI-2/ERIC-PPCI study (CONDI-2: effect of remote ischemic conditioning on clinical outcomes in STEMI patients undergoing pPCI; ERIC-PPCI: Effect of Remote Ischemic Conditioning on clinical outcomes in ST-segment elevation myocardial infarction patients undergoing Primary Percutaneous Coronary Intervention) [185] have demonstrated a further decrease with a cardiac mortality of 2.1%, rehospitalization for heart failure at 12 months of 6.4% and a composite endpoint incidence of 8.0%. An effect size of 25% relative reduction in the event rate is rationalized by proof-of-concept clinical studies, in which remote ischemic conditioning was reported to induce 30% reductions in infarct size [185]. To demonstrate a 25% reduction in the primary composite endpoint in the intervention group (from 8.0 to 6.0%), with 80% power and at the 5% significance level, will require 2554 patients/treatment arm which equates to 5108 patients in total. With 2% drop-out rate at 12 months, recruitment of approximately 5200 patients is required.

In further analyses, stratification according to infarct location, TIMI (Thrombolysis In Myocardial Infarction) flow on admission and collateral flow using analysis of covariance is recommended, and stratification according to duration of chest pain, presence of preinfarction angina and presence of comorbidities (predominantly diabetes mellitus) is encouraged. Power calculations must be provided to ensure the validity of the stratified analyses.

### Specific guidance for power calculations and statistics in clinical cardiac surgery trials

For proof-of-concept studies using biochemical ischemic markers of myocardial injury, the geometric mean (95% CI) of the area under the curve for the biomarker concentration in serum, calculated according to the trapezoidal rule, is compared between standard treatment and the cardioprotective intervention group. The reduction of biochemical marker release by a cardioprotective intervention has varied from no effect to up to 43% reduction by remote ischemic conditioning in previous studies [202]. Although not powered for clinical outcome, a previous study demonstrated that a 17.3% reduction in the troponin-I (TnI) area under the curve by remote ischemic conditioning translated into a clinical benefit in terms of increased survival and lower numbers of MACCE [421]. Sample size also depends on the requirement of study power. Because outcome following cardiac surgery is favorable, a high power is desirable. In the study by Thielmann et al., a power (1-beta) of 95% was used, and with reduction of 20% in the area under the curve TnI release over 72 h, a total of 162 patients was required in each group. With a power of 80%, the number decreases to 98 patients in each group.

For clinical outcome analysis, the event rate of the composite endpoint after 12 months varies between 14 and 28% [182, 302]. Earlier data indicate that 20% of participants undergoing cardiac surgery have MACCE within 12 months [274]. A reduction of an event rate of 25% has been assumed to be associated with improved short- and long-term outcome. With a power of 80% and a *P* value < 0.05, 905 patients are required in each group.

For two-group studies, *t* tests are appropriate for protocols. Stratification according to presence of comorbidities (predominantly diabetes mellitus) and medications is encouraged. Power calculations must be provided to ensure the validity of the stratified analyses.

## Isolated cardiomyocytes

Adult cardiomyocytes are terminally differentiated non-replicative cells. They have unique rod-shaped morphology and are one of the largest cells of the body (around 150 µm length). For their continuous contraction–relaxation activity, cardiomyocytes experience cyclic increases and decreases in intracellular calcium concentration, initiated and terminated by the sequential activation of the sarcolemma (membrane depolarization), sarcoplasmic reticulum (calcium release and reuptake mechanisms) and mitochondria [energy demand–supply matching through adenosine triphosphate (ATP) generation].

Cardiomyocytes are the cells with the highest aerobic requirement of the body, a feature that makes them particularly dependent on mitochondrial bioenergetics. Moreover, changes in energy demand occur instantly and experience continuous fluctuations due to variations in contractile activity. To guarantee the supply of ATP and to ensure dynamic energy demand/supply matching, mitochondria occupy as much as 40% of cardiomyocyte volume and are functionally interconnected with other mitochondria and with the sarcoplasmic reticulum. Both electrochemical coupling and energy demand–supply matching rely on an adequate coordination between mitochondria and the sarcoplasmic reticulum, supported by a tight interorganelle connection that facilitates calcium exchange on a beat-to-beat basis [411]. Calcium transferred from sarcoplasmic reticulum through ryanodine receptors drives the activity of the low-affinity calcium uniporter in mitochondria [350]. Indeed, mitochondrial calcium uptake is more dependent on the amount of calcium released by ryanodine receptors than on bulk cytosolic calcium concentration [411]. Mitochondrial calcium uptake modulates the activity of several enzymes of the Krebs cycle and provides the reducing substrates necessary for the regeneration of the antioxidant glutathione [289]. Some conditions, such as aging, impair mitochondrial respiratory efficiency and bioenergetic matching by a mechanism related to a defective sarcoplasmic reticulum–mitochondria calcium exchange [135]. This impairment may underlie the transition from healthy towards failing cardiomyocytes [70, 257].

Moreover, cardiomyocytes in vivo work as a functional syncytium, anatomically sustained by tight cell-to-cell contacts among them. Contact points contain thousands of highly specialized intercellular structures, called gap junctions, which directly connect the cytoplasm of two adjacent cells, allowing direct passage of different molecules and ions through a regulated gate system [430]. This communication system is essential for cardiomyocyte function (it allows the transmission of electrical impulses and the passage of some signaling molecules between neighboring cells) but it may also amplify cell damage under certain conditions, such as ischemia/reperfusion, in which necrosis has been shown to propagate from cell-to-cell by a gap junction-mediated mechanism, increasing infarct size [149].

Altogether, the specific morphological traits (big size, rectangular shape) and functional properties (powerful contractile system, huge aerobic metabolism, cell-to-cell connections) of adult cardiomyocytes make these cells particularly vulnerable to the conventional chemical and mechanical dissociation procedures used for cell isolation. Indeed, to disrupt cell-to-cell contacts, hearts must be perfused with calcium-free buffer for no less than 20–25 min. However, cardiomyocytes do not tolerate the restoration of physiological calcium levels once they have been depleted of calcium for more than 2 min without experiencing a pathological response consisting of an abrupt cell shortening and massive myofibrillar hypercontraction (“calcium paradox”). Adhesion to the culture dish surface allowing superfusion and rapid extracellular media change was another important step towards the consolidation of the use of isolated cardiomyocytes in basic and translational cardiac research [399]. Nowadays, most protocols for the isolation and cultivation of adult cardiomyocytes are based on this approach and use collagenase for the enzymatic digestion of the extracellular matrix [321]. Restoration of calcium after calcium-free perfusion is one of the main critical aspects of cardiomyocyte isolation and must be performed gradually, following a step-by-step protocol that inevitably results in some degree of cell damage.

### Protocol for the isolation and culture of adult rat or mouse ventricular cardiomyocytes

#### General aspects

Freshly isolated adult ventricular cardiomyocytes are obtained from retrograde perfusion of the heart after aortic cannulation using a Langendorff system. Usually, hearts from rats aged 3–4 months, with an average weight of 250–350 g, are used for such a protocol. One rat heart is sufficient for 20 culture dishes (1 mL/dish; inner diameter of the culture dish: 35 mm) with an approximate cell density of 1.5 × 10^4^ cells/1000 mm^2^. In principle, this method is based on dissociation of cell–cell contacts between cardiomyocytes by nominal calcium-free perfusion of the hearts and subsequent isolation of the cells with collagenase to disrupt cell contacts with the extracellular matrix. The protocol given here has to be adapted to each individual rat strain. Moreover, we strictly recommend the use of rats of either female or male sex for standardization of collagen content and, therefore, of the collagenase concentration. As the isolation procedure requires at least in part calcium-free perfusion, this will cause a calcium paradox and damage during re-administration of physiological amounts of calcium. This is normally achieved by repeated steps of increasing calcium concentrations. Although in principle the following protocol is valid for the isolation of mouse cardiomyocytes, too, the smaller size of mouse hearts makes the cannulation of the aorta more difficult. This is generally performed under a binocular loupe or magnifying glasses. The final cardiomyocyte yield obtained from mouse hearts is substantially lower than that from rat hearts. Also, because mouse cardiomyocytes are more prone to develop hypercontraction, Powell medium should contain either a contractile blocker (i.e. 10 mmol/L of 2,3-butanedione monoxime), or alternatively re-administration of calcium to mouse cells should contain more steps. Within this part, the protocol will be described for rat hearts.

#### Preparation of media and reagents for the isolation

##### Powell medium (1 L)

6.43 g NaCl (110 mmol/L)

0.19 g KCl (2.5 mmol/L)

0.16 g KH_2_PO_4_ (1.2 mmol/L)

0.3 g MgSO_4_·7H_2_O (1.2 mmol/L)

5.96 g 4-(2-hydroxyethyl)-1-piperazineethanesulfonic acid (HEPES) (25 mmol)

1.98 g d(+)-glucose monohydrate (10 mmol/L)

1 L sterile distilled water

Adjust pH with NaOH (2 mol/L) to 7.4 and filter (0.7 μm) the medium under sterile conditions. Store Powell medium at 4 °C.

##### Calcium chloride stock (CaCl_2_)

Prepare a 100 mmol/L CaCl_2_ solution (50 mL) and make aliquots containing 500 μL CaCl_2_. Freeze aliquots at − 20 °C.

##### Media

Three different media are required for the isolation and cultivation of myocytes. A pre-plating medium prepares culture dishes for attachment of cells. A plating medium is used to resuspend the cells after isolation from the heart and to plate them on the culture dishes. A washing medium is used to wash culture dishes after attachment of calcium-tolerant rod-shaped cardiomyocytes. The basis of all three media is called CCT medium, which is a modified Medium 199.

**CCT medium**

1 L of medium 199

3.6 g HEPES

(mix for 1 h)

655.5 mg creatine (5 mmol/L)

395.4 mg carnitine (2 mmol/L)

625.5 mg taurine (5 mmol/L)

Cytosine β-d-arabinofuranoside (10 μmol/L)

Penicillin/streptomycin (2% vol/vol)

Adjust the pH with NaOH (2 mmol/L) to 7.4

Filter the medium under sterile conditions

Store the CCT medium at 4 °C

*Pre*-*plating medium* Use 20 mL CCT medium, as described above, and add 4% (vol/vol) fetal calf serum (FCS). Note: FCS is batch dependent. Ask manufacturers for free samples of FCS and test different batches for attachment. Order the best one. Dependent on the FCS batch you use, the amount of FCS has to be increased or slightly decreased. However, in most cases, 4% (vol/vol) FCS will work. Put 1 mL of the pre-plating medium 2–12 h prior to the cell isolation on culture dishes (inner diameter: 35 mm) and incubate over night in a cell culture apparatus. If you want to use mouse myocytes, replace FCS by laminin (0.5 µg/cm^2^). If you also discover problems with FCS for rat myocytes, laminin can also be used for rat myocytes. For confocal microscopy studies, coat glass-bottom dishes for 2 h with laminin (50 µL of laminin at 50 µg/mL for a 12-mm-inner-diameter dish).

*Plating medium* Use CCT medium, as described above.

*Washing medium* Use CCT medium, as described above. If cells are cultivated for longer time periods (> 48 h) add 20% (vol/vol) FCS to the medium. Please note again, that the bioactivity of FCS is batch dependent.

*Culture dishes* Use preferentially culture dishes with an inner diameter of 35 mm (Falcon 3004). Avoid using any plastic dishes specifically prepared for primary cells that use collagen I or poly-d-lysine as attachment substrate because cardiomyocytes require either a mix of attachment gradients from FCS or laminin, but will not properly attach to collagen I or poly-d-lysin.

##### Materials

For the isolation of the myocytes you need a Langendorff aparatus, standard laboratory centrifuges, water baths and a motor-driven chopper (McIlwain Tissue Chopper, Campden Instruments, UK). The volumes given in this protocol may differ depending on the size of the Langendorff apparatus and might need adjustment according to the specific apparatus.

#### Cell isolation

Warm the plating medium and washing medium to 37 °C. Defreeze a tube of 500 μL CaCl_2_ and weigh in 25 mg of collagenase. Flush the Langendorff perfusion system with sterile distilled water, and afterwards let the Powell medium circulate through the system for 5 min. Fill the Langendorff perfusion system with 80 mL Powell medium, ensuring that it does not contain any air bubble, and gas the medium with carbogen (95% O_2_/5% CO_2_). Prepare a tube (50 mL) with 40 mL Powell medium, warm it to 37 °C, and gas it with carbogen. Prepare a thread of about 25 cm in length for fixing the heart to the cannula. Degrease a razor blade with alcohol (70% by volume) and fasten it to the motor-driven chopper. Clamp a plastic disk into the chopper.

Anesthetize the rat with 4–5% isoflurane and sacrifice it with cervical dislocation. Alternatively use 150 mg/kg of sodium pentobarbital because isoflurane may induce cardioprotective effects on its own. However, whether or not isoflurane affects cardioprotection in the isolated cell model is not clear. Note, the procedure on how to sacrifice the rat depends on the requirements of local authorities. Open the abdomen below the costal arch with an abdominal incision and, with the same pair of scissors, cut through the diaphragm to open the thoracic cavity. Remove the heart, together with the lungs and thymus, by cutting above the thymus. Transfer the material to ice-cold saline solution immediately. Remove the lung and thymus from the heart with dissecting scissors (large) and transfer the latter to a new saline solution. Remove excess tissue, such as residues of thymus, trachea, fat, and connective tissue from the heart using capsule forceps and dissecting scissors (large or small). Uncover the aorta and sever it with dissecting scissors (large or small) between the first and second brachial arch. Before cannulation, open the tap of the Langendorff system and allow the buffer to start dripping.

To initiate perfusion, insert the cannula of the Langendorff system through the aorta and fix it first with a crocodile clamp, and then by tying with the prepared thread tightly around the aorta where it covers the cannula. To ensure a high-quality preparation of cardiomyocytes, the time between extracting the heart from the body and the onset of perfusion should be as short as possible. A more prolonged time causes damage to the heart and results in a higher number of non-viable cardiomyocytes. Rinse the heart until it is free of blood. Immediately, dissolve 25 mg collagenase in 5 mL warm Powell medium and add 12.5 μL CaCl_2_ (end concentration of CaCl_2_ in 50 mL perfusion buffer: 25 μmol/L). Several commercially available batches of collagenase are available, each of them with a different quality and activity. It is recommended to test different samples and to adapt the time of digestion and the amount of collagenase for each new batch. Accordingly, the concentration and time of digestion described in this protocol can differ slightly from other protocols. To start the digestion of the heart, add the dissolved collagenase to the perfusion system. Start the perfusion for 25 min with a drop velocity of 1 drop/s. During perfusion, the heart should swell and develop a waxy appearance. Stop the perfusion after 25 min and remove the heart from the Langendorff perfusion system. A detailed video describing the initial steps is available [321]. Recently, an alternative method of isolation of cardiomyocytes using direct needle perfusion of the LV ex vivo and without the need for Langendorff perfusion has been reported [4].

For progressive calcium restoration, remove and discard the aorta, atria, and connective tissue from the heart, then open the right and left ventricles. Chop the heart twice at an angle of 90° (cutting width 0.7 mm; velocity 0.15 cm/s). Note, if no chopper is available, this step might be performed by quick cutting of the tissue with scalpels. Repeat this process manually with two scalpels for 10 s each side. Transfer 12 mL of the perfusion medium into a new tube (50 mL). Pour the cell slurry into this medium and digest cells for another 5 min at 37 °C. Mix the solution by pipetting up and down every minute. Filter the solution with the digested heart through a nylon mesh (200 μm) into a new tube (50 mL). Centrifuge the filtered solution at 29×*g* for 3 min. Discard the supernatant and add 6 mL warm Powell medium with 12.5 µL CaCl_2_ (210 μmol/L) to the cell pellet. Resuspend the pellet through smooth shaking movements. Centrifuge again at 29×*g* for 2 min. Discard the supernatant and add 6 mL warm Powell medium enriched with 25 μL CaCl_2_ (420 μmol/L). Resuspend the cell pellet through gentle shaking movements and add 12 mL warm Powell medium including 120 μL CaCl_2_ (1 mmol/L). Centrifuge for a third time at 16×*g* for 1 min. Again, discard the supernatant. All these steps can be seen on [321].

**Fig. 4:**
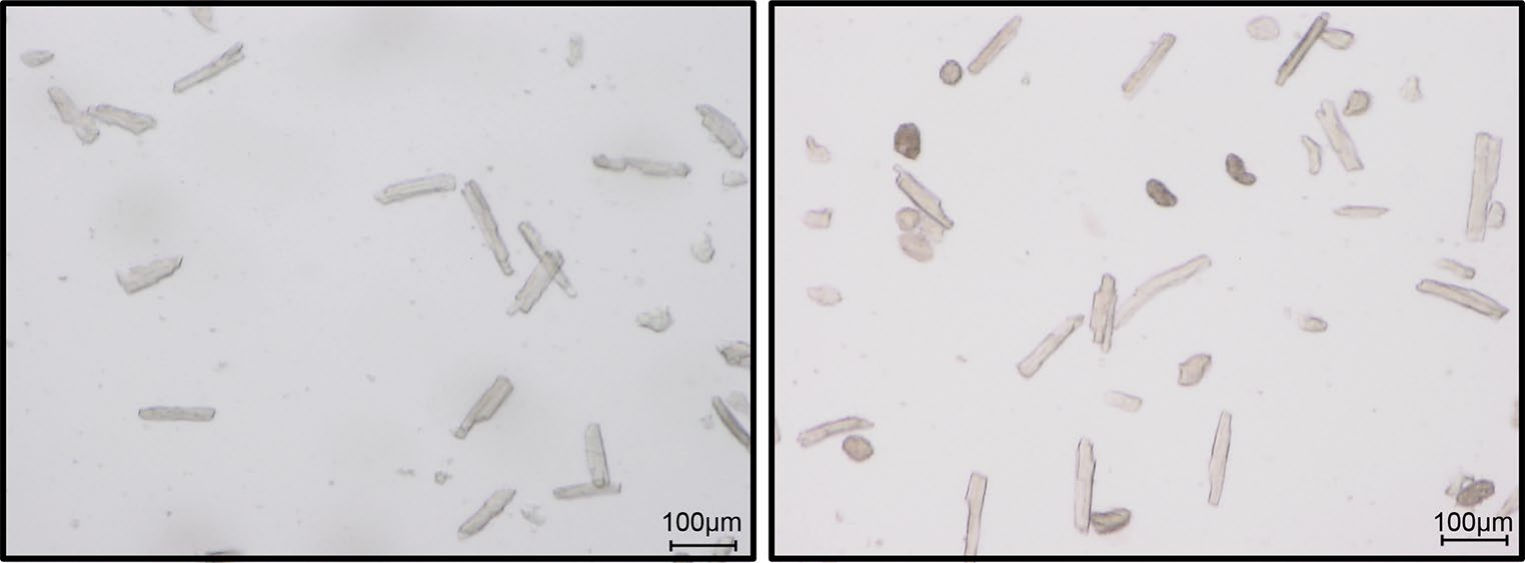
Representative figure of a good cell preparation (left) and a bad preparation (right). Please note the difference in the number of rod-shaped cells

For plating, mix the cell pellet with the pre-warmed plating medium. Remove pre-plating medium from culture plates. Transfer 1 mL plating medium, including the isolated cardiomyocytes, to each culture plate. Incubate freshly isolated cardiomyocytes at 37 °C for 1 h. Thereafter, a quality control should be performed. This includes a simple microscopic view (Fig. 4) and quantification of the number of rod-shaped cells (normally 60–80%; cultures should not be used with less than 70% rod-shaped cells).

Freshly isolated healthy cardiomyocytes are quiescent cells unless they are electrically stimulated. However, they can exhibit spontaneous contractions, calcium transients and calcium sparks. The more mechanical activity they have, the less stable they are in long-term experiments. Spontaneous contractile activity may induce slow calcium overload in cells not fully energized, as calcium reuptake is an ATP-dependent process. In isolated adult cardiomyocytes, cell damage may induce specific morphological changes. Thus, when the intracellular ATP level falls below a critical threshold (50–100 µmol/L), cardiomyocytes develop rigor contracture. In the intact myocardium, rigor contracture is a calcium-independent increase in the mechanical force of the tissue secondary to the formation of cross-bridges between actin and myosin promoted by low ATP conditions [13]. However, in isolated unrestricted cardiomyocytes, development of rigor contracture induces a partial shortening of cell length (square-shaped morphology). The rate and time course of rigor contracture development as well as the final cell shortening can be used to characterize the kinetics of energy depletion in experiments of ischemia, anoxia or metabolic inhibition, and to investigate the effect of some pharmacological interventions [356] or pathophysiological conditions, such as aging [134] on the mechanical fragility of the cells. Although cardiomyocytes with rigor contracture are viable and keep their normal cross-striation pattern, they are rather vulnerable to damage.

Rigor-shortened cells can develop hypercontraction upon reperfusion or reoxygenation. During cardiomyocyte isolation, excessively long procedures or inadequate oxygen bubbling during manipulation may result in a significant proportion of cells with square morphology as a consequence of rigor development.

Cardiomyocytes may undergo a specific form of damage consisting of an abrupt shortening associated with an irreversible distortion of cell architecture, usually known as hypercontraction. Hypercontraction is facilitated by the powerful contractile machinery of the adult cells that tends to overreact in the presence of abnormally elevated calcium level. Whereas in intact myocardium, hypercontraction induces sarcolemmal disruption and cell death, and isolated cardiomyocytes can freely shorten, undergoing irreversible rounding-up and preserving sarcolemmal integrity, due to the absence of the mechanical restriction imposed by the interaction with other cells. Rounded cells can be metabolically competent when the sarcolemma has not been damaged [383]; nevertheless they lose their normal morphology, do not adequately attach to the culture dish and are discarded during washing steps.

The quantification of the number of rounded cells relative to the number of rod-shaped cardiomyocytes is the index used to establish the quality of the cell preparation. In general, a good preparation of freshly isolated adult cardiomyocytes should contain ≥ 70% of rod-shaped calcium-tolerant cells. Nevertheless, even after an optimal isolation procedure, a significant proportion of cardiomyocytes develop calcium paradox-induced hypercontraction. It is important to take into account that the susceptibility of cardiomyocytes to isolation protocols may vary between animal species and depend on their age.

#### Cultivation of cardiomyocytes

Remove the plating medium from culture plates. Add 1 mL washing medium to each culture plate and store the plates at 37 °C for up to 6 days without changing the medium. For investigating the influence of different chemicals and treatments on cardiomyocytes, first refresh plating medium by washing medium and thereafter add different chemicals. Under these conditions, cardiomyocytes survive as rod-shaped cells for 48 h and can be used for further analysis such as growth control or function. Measuring the cell size enables growth control. Measure the long axis of the cells (*l*), and the diameter of the cell (*d*) at the middle of the long axis. Calculate cell volume according to the equation Volume = *l* × (*d*/2)^2^ × *π*, based on the assumption that myocytes display a cylindrical cell shape. The function of the cell can be analyzed by load-free cell shortening placing two silver electrodes into the cell culture medium and stimulating the cells between 0.5 and 2.0 Hz at room temperature [321].

Long-term cultures of cardiomyocytes can be performed by replacing serum-free media by media containing 20% (vol/vol) FCS. Under these conditions, cells rapidly (within 24 h) round up and reorganize their contractile apparatus. To perform an evaluation of the cultured cardiomyocytes throughout time with light microscopy, monitor 150–300 cardiomyocytes/day by light microscopy. Subdivide all counted cardiomyocytes into groups according to their appearance (e.g. “rod-shaped”, “round down”, “spreading”, and “unusual appearance”). The category “spreading” includes all cardiomyocytes with pseudopodia-like structures. “Unusual appearance” includes those cardiomyocytes that have an irregular surface and no detectable intact cell membrane. Please note that transfer of this rat cell protocol to mouse cardiomyocytes does not work.

### Some specific experimental cardiomyocyte models

#### Membrane-permeabilized cardiomyocytes

For sarcolemmal permeabilization, plated cardiomyocytes are washed with an “intracellular-like buffer” at 37 °C containing 5 mmol/L MgCl_2_, 10 mmol/L HEPES, 250 mmol/L sucrose, 25 mmol/L Tris, 0.5 mmol/L ethylene glycol tetra-acetic acid (EGTA), 5 mmol/L succinate, and 4 mmol/L ATP, at pH 7.2. 100 µmol/L digitonin is added for 1 min, cells are washed twice with intracellular-like buffer to remove digitonin and kept at 37 °C with intracellular-like buffer until the onset of the experiment. Alternatively, saponin 0.005% for ~ 30 s can be used for membrane permeabilization. Triton X-100 is discouraged in living cells because it can induce cytoskeletal damage. Membrane-permeabilized cells are extremely sensitive to the presence of calcium in the buffer. Therefore, they should always be kept in intracellular-like buffer in the presence of a calcium chelator, usually 1–2 mmol EGTA. The presence of calcium chelators requires the use of specific calcium titration programs to calculate the real free calcium concentration present in the medium. When membrane lysis is carried out, a concentration of 4 mmol/L (or higher) of ATP should always be present in the buffer to guarantee that cells retain the rod-cell morphology [14]. Cardiomyocytes with an accurate membrane permeabilization are capable of maintaining intact organelles and subcellular microdomains (i.e. sarcoplasmic reticulum–mitochondria communication), and are a good model for studies in which sarcolemmal barrier and membrane transport systems can interfere with the interpretation of the data.

#### End-to-end connected cardiomyocytes

To increase the proportion of end-to-end connected pairs of cardiomyocytes in the final cell preparation, the amount of calcium during collagenase perfusion is slightly increased (i.e. end concentration of CaCl_2_ in 50 mL perfusion buffer: 45–50 μmol/L instead of 25 µmol/L). Pairs of end-to-end connected cardiomyocytes may be used for electrical propagation and cell-to-cell communication studies.

### Use of isolated cardiomyocytes

Isolated cardiomyocytes can be used in a vast array of experiments measuring different aspects of their biology or pathophysiology. The most important applications are: (1) studies on tolerance to real or simulated ischemia/reperfusion or anoxia reoxygenation and the effect of interventions on cell damage/protection. This is a classical model in cardioprotection studies, and may involve fluorometric monitoring of ionic concentrations in the cytosol and other cell compartments (mitochondria, sarcoplasmic reticulum), immunofluorescence (protein interaction and trafficking), and biochemical analyses (signaling pathways, protein expression, enzyme release); (2) studies on contractile function in different conditions and response to interventions. This approach requires field stimulation of cardiomyocytes [190]; (3) electrophysiological studies, involving recording of electrical activity and impulse propagation. This requires introduction of micropipettes into the cytosol of cardiomyocytes for patch clamp studies and may be combined with cell imaging-based monitoring of intracellular ionic movement.

#### Isolated cardiomyocytes for pathophysiological studies

##### Mitochondrial de- and re-energization during ischemia/reperfusion

For ischemia/reperfusion experiments in isolated cardiomyocytes, two different approaches can be used: anoxic work-station under controlled atmosphere of 0% O_2_–95% N_2_–5% CO_2_ at 37 °C or microscope-adapted microperfusion chamber. In the first approach, an unlimited number of plated cardiomyocytes can be simultaneously incubated in a glucose-free acidic ischemic buffer and reoxygenated. The microscope-adapted microperfusion chamber is used for confocal microscopy studies, in which the kinetics of intracellular ionic changes or mitochondrial integrity and energy status, among other variables, can be investigated throughout time at the single-cell level from high-resolution images. For the anoxic work-station, cardiomyocytes are placed within the anoxic atmosphere and incubated for the desired time. When working with absolute anoxia (not hypoxia), the duration of incubation in O_2_-free atmosphere should not exceed 40–50 min, to get a significant proportion of surviving cells upon reperfusion (different times of anoxia must be tried out in each specific setting). To better simulate in vivo ischemia, use glucose-free acidic ischemic buffer previously deoxygenated in an autoclave and bubbled with N_2_ for 20 min to ensure that it will not introduce O_2_ to the chamber. Ischemic buffer contains 140 mmol/L NaCl, 3.6 mmol/L KCl, 1.2 mmol/L MgSO_4_, 1 mmol/L CaCl_2_, and 20 mmol/L HEPES at pH 6.4, and it is supplemented with 4 μmol/L resazurin, 100 μmol/L ascorbic acid, 0.5 mmol/L dithionite and 100 U/mL superoxide dismutase. For reperfusion, ischemic buffer has to be gently washed out (to avoid detachment of rigor-contractured cardiomyocytes) and the chamber oxygenated with glucose-containing control buffer at pH 7.4.

To simulate ischemia/reperfusion at the single-cell level, a glass-bottom dish with laminin-attached cardiomyocytes is placed on the stage of an inverted microscope. Cells are superfused with the aid of a peristaltic pump within a closed microperfusion chamber (airtight chamber adapted to the specific microscope setting) with the ischemic buffer at pH 6.4, continuously bubbled with N_2_. Reoxygenation is induced by switching to oxygenated, glucose-containing control superfusion, at pH 7.4. The effect of ischemia/reperfusion on cell morphology can be simultaneously assessed by quantification of the number of cells developing rigor shortening during oxygen deprivation (defined as a 25–40% reduction of cell length with preserved squared-shape morphology) and hypercontraction (defined as > 70% reduction of cell length with concomitant disruption of cytoarchitecture) upon reoxygenation.

##### Pelleted freshly isolated cardiomyocytes

Isolated cardiomyocytes obtained immediately after the isolation procedure may be plated in culture dishes (as indicated above) or pelleted after a low speed centrifugation step. Pelleted cardiomyocytes have many of the advantages of plated cardiomyocytes (no contribution of other cell types, absence of cell-to-cell interaction, intact and fresh preparations) but may offer additional advantages in some specific contexts, mainly for in vitro ischemia/reperfusion and preconditioning experiments [21, 431]. For the in vitro ischemic model, excess supernatant is removed (so that the fluid over the cells is extremely thin) and the pellet containing the viable cardiomyocytes is covered with a mineral oil layer and incubated at 37 °C for 30–120 min. Degree of ischemic damage can be determined with a viability staining method in cells obtained from the ischemic pellet at different time points. Reperfusion is simulated by replacing the oil layer with a glucose-containing oxygenated buffer and gentle resuspension of the cells. This model also allows to precondition the cells using brief periods of ischemic pelleting before the index ischemia [109] or by application of drugs [22] and has been widely used for the quantification of cell death, sarcolemmal osmotic fragility and ultrastructural changes associated with ischemia/reperfusion injury [191, 406]. Pelleting model with an oil layer exposes the cells to low-oxygen environment while reducing the extracellular space. These conditions induce the progressive exhaustion of metabolites and favor the accumulation of lactate and the simulation of the acidosis present in the in vivo ischemia with great fidelity.

### Modes of cell death and cellular viability assessment

Although detection of cell death may appear a simple task, several factors complicate it. The definition of cell death may be difficult when it occurs through a complex process during which cells may be still living, i.e. may retain metabolic activity and trans-membrane ionic gradients, although they have reached a no-return point in the process of cell death [442].

Cardiomyocyte cell death is particularly important in determining the clinical consequences of myocardial ischemia and reperfusion. Cardiomyocytes perform cardiac work and, in the adult heart, have little or no proliferative capacity [37]. In addition, their elevated energy requirements and dependence on aerobic metabolism, and their powerful contractile machinery coupled to ample and precise mitochondrial–sarcoplasmic reticulum calcium movements render cardiomyocytes more susceptible to ischemia/reperfusion than other cell types present in myocardium such as endothelial cells, smooth muscle cells or fibroblasts [235].

#### Cardiomyocyte necrosis

Necrotic cell death is characterized by the loss of cell membrane integrity and acute loss of cellular ATP [442]. In the context of ischemia/reperfusion, cardiomyocyte necrosis is generally associated with gross morphological changes as a consequence of the associated hypercontracture [150, 151]. There are two main types of experiments in which cell necrosis can be studied: cell preparations and myocardial tissue.

In isolated cardiomyocytes, necrosis can be demonstrated by a variety of methods based on the detection of membrane impermeable dyes within the cell. The two most widely used dyes are trypan blue and propidium iodide. In both cases, the marker is added to the extracellular media, the cells are washed and the presence of the marker within the cell is investigated. For the trypan blue test, a 0.4% solution of the dye is prepared in isotonic phosphate-buffered saline at pH 7.4 and added to the cell suspension or to the cells plated in a culture dish in a 1:1 volume for 2–3 min. Cell death is calculated as the number of blue stained (non-viable) cells divided by the total number of cells. Trypan blue-positive cells as well as the total number of cells may be detected and counted by microscopy. The results are in general clear, but the wash-out protocol and the timing of the readout as established in the protocol should be carefully followed. Longer incubations (> 10 min) may decrease cell viability and distort the results due to dye toxicity. Propidium iodide binds to nucleic acids in cells with permeabilized plasma membrane and emits red light, between 600 and 700 nm, when excited with 400–600 nm light. Usually, a volume of 5 µL of propidium iodide is added to 100 µL of the buffer containing the cells for 5–10 min. Counting of propidium iodide-positive cells can be performed by microscopy or flow cytometry, the latter having the advantage of allowing an automatic analysis of larger numbers of cells. Importantly, total cell count must be determined. Other tests, such as cell-permeable calcein acetoxymethyl labeling, are based on the ability of the AM esters to cross the plasma membrane of living cells. Once the non-fluorescent dye reaches the cytosol, it is hydrolyzed by intracellular esterases to green-fluorescent calcein. A standard protocol for cell loading includes 30 min of cell incubation in the presence of 1 µmol/L calcein acetoxymethyl ester at 37 °C followed by a washing step. Fluorescent-positive cells (viable) can be automatically measured using a fluorescent plate reader (excitation: 490 nm/emission: 520 nm).

Isolated adult cardiomyocytes, which undergo necrosis upon reoxygenation or reperfusion, invariably show hypercontracture. Unlike rigor contracture, hypercontraction is an energy-dependent response and can be considered the histological hallmark of reperfused myocardial infarcts [150].

However, hypercontracted cardiomyocytes may retain sarcolemmal integrity and metabolic activity if shortening is not restrained by firm physical interactions with other cells or surfaces [383]. Although these cells are alive, it may be reasonable to assume that they would have experienced sarcolemmal rupture and death if hypercontracture would have happened within intact myocardial tissue [30, 148]. Thus, to have a more informative picture of the effects of interventions on reperfusion injury in isolated adult ventricular cardiomyocytes, it is important to report the number of cells undergoing hypercontracture. Hypercontracted cardiomyocytes are clearly identifiable by visual inspection in microscopic fields and can be also automatically detected by flow cytometry [284].

Under certain conditions (i.e. osmotic stress) or in the presence of sarcolemmal fragility (induced by aggressive collagenase digestion) hypercontraction of isolated cardiomyocytes may be associated with sarcolemmal rupture and death [355].

Viability tests may provide information, which goes beyond the concept of sarcolemmal rupture. The [3-(4,5-dimethylthiazol-2-yl)-2,5-diphenyltetrazolium bromide] tetrazolium (MTT) reduction assay is a high throughput viability screening suitable for a 96-well format, although in small-well formats variability of readouts tends to be high between replicates. For the reaction, 0.2–0.5 mg/mL MTT substrate is dissolved in physiological buffer and added to the cells for 1–4 h at 37 °C. Viable metabolically competent cells convert MTT into a purple-colored formazan product that can be measured as a proportional increase in absorbance at 570 nm using a spectrophotometer. The particularity of this assay is that reduction of MTT into formazan seems to involve mitochondrial enzymes which transfer electrons to MTT. Therefore, it may be useful for measuring the lack of mitochondrial activity [41].

Cardiomyocyte necrosis can be indirectly assessed by measuring enzyme release. Lactate dehydrogenase (LDH) is the most widely used enzyme for this purpose and can be accurately measured in cell culture media or in the effluent of perfused hearts or myocardium using both a NADH-linked ultraviolet–visible spectrophotometric method or a NADH-linked LDH fluorescent assay.

In experiments using cardiomyocyte preparations, LDH should be normalized with respect to total LDH content of the preparation, as measured after inducing sarcolemmal rupture or permeabilization by osmotic or chemical methods.

#### Other forms of cell death

The possible contribution of cardiomyocyte apoptosis to reperfusion injury has been investigated in many studies [77]. However, recent data strongly support the notion that apoptosis has little or no role in reperfusion-induced cell death of adult cardiomyocytes. First, these cells do not express anymore the caspases necessary to execute the apoptotic program [364]. Moreover, genetic deletion of caspase 3 and 7 has no effect on infarct size or postinfarction remodeling in mice [222]. It is clear that embryonic or neonatal cardiomyocytes and modified myocyte lines such as HL1 or H9c2 cells can undergo apoptosis, but it is doubtful whether studying apoptotic pathways and targets in these cells can be relevant to cardioprotective interventions in adult patients.

Recently, another form of programmed cell necrosis has been described, known as necroptosis, in which the execution of a complex chain of events leads to cell swelling, rupture of the plasma membrane, release of cytosolic contents and activation of the innate immune response [5]. Activation of the receptor interacting kinase 3 (RIP3) by receptor interacting kinase 1 through the formation of a receptor interacting kinase 1/3 complex is an essential part of this chain of events [78] and requires blockade of caspase activation. A number of methods have been devised to detect necroptosis in isolated cells, histological preparations and even in vivo. As necroptosis leads to disruption of cell membranes, conventional methods to assess loss of sarcolemmal integrity, such as cell-impermeant dyes that bind to deoxyribonucleic acid (DNA) or enter the cytosol (propidium iodide, 4′,6-diamidino-2-phenylindole (DAPI), trypan blue), followed by imaging techniques or flow cytometry, can be used for its detection. Nevertheless, identification of necroptosis is challenged by the fact that cells readily shift from apoptosis to necroptosis and necrosis. Therefore, assessment of necroptosis should be complemented with time-dependent analysis of morphological changes and other technical approaches specifically targeted to the involved signaling pathways. In this respect, assessment of mitochondrial membrane potential with fluorescent probes which accumulate in healthy mitochondria, such as tetraethyl-benzimidazolyl-carbocyanine iodide(JC-1), methyl ester of tetramethylrhodamine (TMRM), and ethyl ester of tetramethylrhodamine (TMRE), may be a useful tool to discriminate between necroptosis and necrosis, as hyperpolarization of mitochondrial membrane potential (resulting in a transient increase in fluorescence) has been described in the early stages of necroptosis. Although it has been shown to occur during ischemia in different cell types, there is not sufficient evidence for necroptosis of cardiomyocytes during reperfusion.

### Limitations of the cardiomyocyte cell model

The main limitation of the isolated cardiomyocyte model is that it does not consider interactions with other myocardial cells, in particular endothelial cells, fibroblasts and macrophages. While this simplification may be useful in mechanistic studies, it can be misleading. This limitation is partially overcome by different co-culture methods in which other cells types, for example fibroblasts, endothelial cells and pericytes, are added to the preparation either in the same chamber or in separate chambers communicating through filters or diffusible membranes [66]. This technical solution may be useful for specific purposes, such as for studying cardiomyocyte–fibroblast interaction through connexin 43 gap junctions. Isolated cardiomyocytes are quiescent cells. This feature eliminates the main energy expenditure of adult cells. Field stimulation simulates in vivo contractile activity; however, continuous pacing is not practical (nor stable) in prolonged studies. The fact that isolated cardiomyocytes are physically unrestrained, except for their adherence to the well, makes them prone to develop exaggerated rigor contracture and hypercontraction. Moreover, hypercontraction does not necessarily induce sarcolemmal rupture and cell death, in contrast to in situ myocardial hypercontraction. Although they can be maintained in culture, cardiomyocytes are postmitotic cells that rapidly lose their adult elongated phenotype. This makes it very difficult to use them for genetic modification approaches. Importantly, high-quality preparations of adult isolated cardiomyocytes are difficult to obtain and require well trained and experienced technicians. When preparations are not good enough, studies can still be conducted with the remaining few rod-shaped cardiomyocytes; nevertheless, this may introduce a selection bias. All laboratories experience problems from time to time with cardiomyocyte quality that may be very difficult to tackle and correct; occasionally, these problems persist for several weeks.

### Future perspectives of isolated cardiomyocyte models

The technology for cell physiology studies keeps evolving. The development of powerful confocal microscopy platforms, with increasingly sophisticated imaging acquisition software, runs in parallel with the development of novel fluorescent probes and methods to selectively target subcellular compartments. This technological development will bring a long and fruitful future to the cardiomyocyte model that may have further potential by co-culturing with other cell lines.

## Involvement of mitochondria in cardiomyocyte injury

Mitochondria are involved in the major functional and structural derangements characterizing the transition from reversible to irreversible injury during cardiac ischemia and reperfusion [108]. As outlined in the chapter "Isolated cardiomyocytes", during the early phase of ischemia, the failure of contraction and the rigor contracture, which are the most relevant changes in myocardial function, are caused by intracellular acidosis and severe reduction in ATP content, respectively. Then, the sudden changes produced by reperfusion on myocardial viability are concomitant with massive accumulation of calcium within the mitochondrial matrix. This seminal finding that highlighted the role of intracellular Ca^2+^ overload in myocardial pathology [380] indicates that the rapid transition towards cell death requires a coupled mitochondrial respiration. In fact, pre-ischemic respiratory chain inhibition or uncoupling of oxidative phosphorylation blunts enzyme release occurring at the onset of post-ischemic reperfusion [123, 145] and preserves mitochondrial function [226, 405]. Therefore, oxidative phosphorylation is essential for cell recovery, but can also contribute to the processes resulting in cell necrosis.

Upon reperfusion, mitochondria are exposed to high levels of Ca^2+^ that is increased in the cytosol due to both the failure of Ca^2+^ ATPases and uptake through the sarcolemmal Na^+^/Ca^2+^ exchanger. Since mitochondrial Ca^2+^ uptake and ATP synthesis utilize the same driving force, namely the mitochondrial membrane potential ΔΨ_m_, mitochondrial Ca^2+^ accumulation results inevitably in a significant decrease in ATP formation. In addition and importantly, the rise in intramitochondrial [Ca^2+^] ([Ca^2+^]_*m*_) promotes opening of the mitochondrial permeability transition pore (mPTP) and is associated with increased formation of reactive oxygen species (ROS). Eventually, a vicious cycle is generated whereby high [Ca^2+^]_*m*_, mPTP opening and ROS formation hamper mitochondrial function and jeopardize the maintenance of cell viability.

### Isolation of subsarcolemmal and interfibrillar mitochondria from cardiac tissue

To investigate mitochondrial function in detail, they must be purified in sufficient quantity and retain their functional capacity. The following protocols describe the isolation of two different mitochondrial subpopulations, subsarcolemmal (SSM) and interfibrillar (IFM) mitochondria, which are used to study mitochondrial function in terms of oxygen consumption, ROS formation, or calcium retention capacity. To date, there is no protocol available to purify perinuclear mitochondria which are clustered around the nucleus. Depending on the subpopulation(s), two different protocols can be followed. All steps of the isolation procedure are performed at 4 °C. In the following, focus is on isolation from mouse heart tissue. However, all processes can be translated to heart tissue of rats and pigs with appropriate adjustment of buffer volumes to tissue mass.

#### Isolation of subsarcolemmal mitochondria (SSM) only

Isolation buffer (prepared at the day of the isolation or filter-sterilized before use):

250 mmol/L sucrose

10 mmol/L HEPES

1 mmol/L EGTA

pH 7.4

Fatty acid-free bovine serum albumin (BSA, 0.5%) is added to the isolation buffer during the homogenization steps to protect the organelles from damage during the isolation process and to remove free fatty acids. After homogenization, mitochondria are kept in isolation buffer without BSA, supplemented with proteinase and/or phosphatase inhibitors.

Mice are sacrificed according to the recommendations of local authorities, e.g. by cervical dislocation without or with prior anesthesia with 5% isoflurane. Hearts are rapidly removed and placed immediately in a small volume (10 mL) of isolation buffer with BSA. Tissue is minced with a small scissor, and the buffer is changed several times to remove as much blood as possible. If mitochondria are isolated from Langendorff-perfused hearts, there is no need to wash the tissue. The tissue is further homogenized using an UtraTurrax (IKA, Staufen, Germany; two steps of 10 s, rotation rate 6500 rpm; samples are put on ice during homogenization, with 1 min intervals between homogenization steps to avoid warm-up of the mitochondria) or with a Teflon pestle in a glass potter (five strokes, pre-cooled glassware and pestle).

After homogenization, samples are transferred to fresh tubes and centrifuged at 700*g* for 10 min to remove intact cells and nuclei. The supernatants containing the SSM are transferred to fresh tubes and centrifuged at 14,000*g* for 10 min. The SSM, which are now sedimented, are resuspended in 1–2 mL isolation buffer (without BSA) and washed by centrifugation at 10,000*g* for 5 min. After this centrifugation, the mitochondria are resuspended in a small volume (150–500 µL) of isolation buffer, depending on the initial amount of tissue.

#### Isolation of both subsarcolemmal (SSM) and interfibrillar (IFM) mitochondria

The distinction between SSM and IFM has first been made by Palmer and colleagues [327]. The protocol presented here follows that reported by Judge and colleagues [231] with slight modifications. SSM and IFM differ in their respiratory capacity and in their calcium retention capacity, in that IFM consume more oxygen than SSM and take up more calcium ions until mPTP opening occurs [52, 327]. The use of the present protocol yields mitochondrial subpopulations with these characteristics. Morphologically, the cristae structure of SSM and IFM differs, in that SSM have mostly lamelliform cristae, whereas IFM have tubular or a mixture of tubular and lamelliform cristae [349]. The isolation of IFM requires the use of a protease to release IFM from the myofibrils. Nagarse is the most used protease (and the present protocol focusses on this enzyme); however, trypsin [92] or proteinase K [359] are also used to isolate IFM.

Homogenization buffer (prepared at the day of the isolation or filter-sterilized before use):

100 mmol/L KCl

50 mmol/L 3-(*N*-morpholino) propane sulfonic acid (MOPS)

5 mmol/L MgSO_4_

1 mmol/L EGTA

pH 7.4

Homogenization buffer with ATP (prepared at the day of the isolation):

The homogenization buffer is supplemented with 1 mmol/L ATP.

Myocardial tissue is removed and washed, as described above. The myocardial tissue is weighed before placing it in homogenization buffer with ATP or in isolation buffer [156, 388]. Tissue homogenization and the first centrifugation step are performed, as described above. Subsequently, SSM and IFM are processed individually. The supernatant of the first centrifugation step contains the SSM, which are further purified as indicated. IFM—together with intact cells, nuclei and debris—are present in the sediment of the first centrifugation step. This sediment is resuspended in homogenization buffer with ATP (10 mL/g myocardial tissue) and transferred to a glass potter. Nagarse (proteinase type XXIV) is added (2–8 U/g), and after incubation for 1 min on ice the sample is homogenized by 2–6 strokes of the pestle [156, 259, 388, 400]. Homogenization buffer with ATP is added to a total volume of 12 mL, samples are then centrifuged at 1000*g* for 10 min, supernatants are collected and centrifuged at 8000*g* for 10 min. The IFM, which are now sedimented, are resuspended in isolation buffer and washed by centrifugation at 8000*g* for 10 min. Finally, IFM are resuspended in a small volume (200–500 µL) of isolation buffer.

The incubation of samples with nagarse is the critical step during the isolation of IFM. If the concentration/activity of the enzyme is too low, IFM are not released; if it is too high, mitochondrial proteins are digested. In fact, immunoreactivity for mitofusin 2 is detected predominantly in SSM. However, after inhibition of nagarse by 1 mmol/L phenyl methyl sulfonyl fluoride, mitofusin 2 is also seen in IFM, indicating an underestimation of mitofusin 2 in IFM using the above protocol [259]. Therefore, protease sensitivity must be considered when assessing the presence and distribution of mitochondrial proteins using nagarse-based isolation.

Using an aliquot of the resuspended mitochondria, the protein concentration of the mitochondrial isolation is determined. For this quantification, a standard assay is used, e.g. the Lowry assay with BSA as external standard.

The above protocols yield mitochondria, which are suitable to study their function. However, these preparations are still contaminated with proteins of other cellular compartments. In case that an investigation of mitochondrial proteins is planned by western blot analysis, a further purification step should be included. The SSM and IFM are then layered on top of a 30% Percoll solution in isolation buffer, and a subsequent ultracentrifugation is performed at 35,000*g* for 30 min. The ultracentrifugation results in two bands, of which the lower one contains the intact mitochondria. The mitochondria are collected, washed twice in isolation buffer by centrifugation at 10,000*g* for 10 min, and the purified mitochondria can be stored at − 80 °C.

### Mitochondrial parameters and experimental models

The evaluation of mitochondrial function depends largely on the model tested (Table 1). The number of parameters that can be assessed in isolated mitochondria is significantly reduced in isolated cardiomyocytes, while only indirect information can be obtained in whole hearts. A relevant exception to the latter is represented by the elegant technique using arylboronic acid conjugated to triphenylphosphonium for the direct detection of mitochondrial ROS formation in vivo [80, 84]. Until now, this method has only been used for detecting the large burst in ROS formation occurring at the onset of reperfusion. The assessment of mitochondrial function in intact organs, including beating hearts, should become feasible by combining the targeted expression of fluorescent proteins with intravital microscopy [437].

**Table 1:** Detectable parameters of mitochondrial function

Preparation	Parameter
Isolated mitochondria	Oxygen consumption
	ATP synthesis
	Redox changes (NAD and FAD)
	ROS formation
	ΔΨ_m_ (quantitative)
	Matrix volume
	Ion movements
Isolated cells	Oxygen consumption
	Redox changes (NAD and FAD)
	ROS formation
	ΔΨ_m_ (semiquantitative)
	Matrix volume
	Ion movements (reliable only with genetically encoded probes)
Whole Hearts	Oxygen consumption
	(severe) mitochondrial ROS formation
	ATP content

*ATP* adenosine triphosphate, *NAD* nicotinamide adenine dinucleotide, *FAD* flavin adenine dinucleotide, *ROS* reactive oxygen species

The limitations imposed by the increasing complexity of the experimental models (i.e. isolated organelle vs whole heart) apply also to the assessment of mPTP opening (Table 2). Differences among the various methods are detailed in the following sections.

**Table 2:** Methods for detecting the opening of the mitochondrial permeability transition pore (mPTP)

Preparation	Parameter
Isolated mitochondria *(or permeabilized cells)*	Swelling
	Ca^2+^ retention capacity (CRC)
	Permeability to solutes
	CsA inhibitable changes
Isolated cells	Calcein redistribution
	Swelling
	CsA inhibitable changes
Whole hearts	Mitochondrial NAD depletion
	Mitochondrial accumulation of tritiated deoxyglucose
	CsA inhibitable changes

CsA: cyclosporine A

### Major methods for evaluating mitochondrial function in vivo

#### Oxygen consumption

Oxygen consumption can be assessed in intact cells, however, without testing the response to adenosine diphosphate (ADP) and specific respiratory substrates. More recently, the Seahorse extracellular flux analyzer (Agilent) has been introduced for the evaluation of oxygen consumption in intact cells. In this device, accurate measurements require the analyzed cells to be grown in a uniform monolayer configuration that cannot be obtained with adult cardiac myocytes. This technique can be adapted to the study of neonatal rat ventricular cardiomyocytes. Neonatal rat ventricular cardiomyocytes (60,000/well) are seeded onto Seahorse 24-well microplates 24 h prior to the analysis. Oxygen consumption rate data consist of mean rates during each measurement cycle consisting of a mixing time of 30 s and a waiting time of 3 min followed by a data acquisition period of 3 min. Rates displayed are basal respiration and rates following addition of 1 μg/mL oligomycin, 250–750 nM carbonyl cyanide-4-(trifluoromethoxy)phenylhydrazone (FCCP), 1 μM rotenone and 1 μM antimycin A. The addition of oligomycin mimics state 4 respiration, while the maximal values obtained with FCCP provide a reliable estimate of the total activity of respiratory chain complexes. The difference between FCCP or baseline and oligomycin values reflects the total respiratory capacity and the coupling efficiency, respectively. Of note, the FCCP concentration must be titrated to determine the optimal concentration necessary to maximize respiration (please note that at high doses FCCP inhibits respiration).

This approach is undermined by two major shortcomings: (1) variations in the number of seeded cells are paralleled by changes in respiration independently of mitochondrial function; (2) during the assay it is not possible to assess the occurrence of cell death that would obviously decrease the rate of oxygen consumption, thus flawing the evaluation of mitochondrial function.

#### Mitochondrial membrane potential

Although mitochondrial membrane potential (ΔΨ_m_) can be measured also in isolated mitochondria, its assessment represents the most common approach for the evaluation of mitochondrial function in intact cells [40].

The aim of cell respiration is to build an H^+^ gradient (ΔΨ_m_) in the intermembrane space of mitochondria that provides the energy necessary for ATP generation. Mitochondrial ATP synthase is the molecular machine that transforms the electrical power accumulated in the H^+^ gradient into ATP-containing chemical energy, following the chemiosmotic principle that governs the life of all organisms: the energy dissipated when H^+^ flows back to the matrix through the H^+^-conducting channel of the ATP synthase is used by the enzyme to catalyze the conversion of ADP into ATP. Therefore, the mitochondrial electrochemical gradient or membrane potential (ΔΨ_m_) results from the balance between H^+^ extrusion and H^+^ dissipation and may be used as an index of the efficiency of mitochondrial respiration and/or degree of respiratory (un)coupling. Several factors, such as high energy expenditure, the presence of pharmacological uncouplers (such as dinitrophenol or FCCP) or the expression of uncoupling proteins may contribute to the dissipation of ΔΨ_m_ which, in turn, reduces ROS production [72]. Under ischemic conditions, lack of O_2_ stops the activity of the electron transport chain through the respiratory complexes, and mitochondria progressively depolarize mainly because H^+^ extrusion to the intermembrane space is interrupted and H^+^ flows back into the mitochondrial matrix through ATP synthase and other uncoupling proteins [360].

Here, one potential and frequent source of misinterpretation is that in isolated cells ΔΨ_m_ can be maintained by the inverse operation of the FoF1 ATPase. In this process, ATP produced by glycolysis is hydrolyzed to maintain the proton gradient across the inner mitochondrial membrane. The addition of the FoF1 ATPase inhibitor oligomycin allows one to discriminate whether ΔΨ_m_ is maintained by the respiratory chain or ATP hydrolysis. This latter possibility is the direct consequence of alterations in respiratory complexes and/or mPTP opening.

The assay is based on the use of lipophilic cations with fluorescence emission that can be monitored by means of fluorescence microscopy. These fluorescent probes are good substrates for the multi-drug resistance (MDR) pump [40]. Thus, different levels of fluorescence can result from variations in MDR expression independently of mitochondrial function. To avoid this problem MDR activity should be inhibited. We suggest use of cyclosporine H, an analog of cyclosporine A (CsA) that inhibits the MDR without affecting mPTP opening.

To measure the ΔΨ_m_, cells are pre-incubated with 25 nM tetramethylrhodamine (TMRM, Thermo Fisher Scientific) in the presence of 1.6 µmol/L cyclosporine H. TMRM (excitation 535 nm and emission 600 nm) is a lipophilic rhodamine dye that accumulates in the mitochondria of live cells depending on their ΔΨ_m_. Fluorescence microscopy is used to generate images that are collected before and after the addition of 2.5 µmol/L FCCP to completely abolish ΔΨ_m_. TMRM fluorescence intensity is expressed as *ΔF* according to the following formula: *ΔF* = *F*_0_/*F*_FCCP_, where *F*_0_ = TMRM fluorescence intensity at baseline and *F*_FCCP_ = fluorescence intensity after addition of FCCP.

#### Mitochondrial calcium homeostasis

Rhod-2 is a high-affinity calcium indicator with a Kd of ~ 570 nmol/L that tends to accumulate in the mitochondrial matrix following the negative electrochemical gradient; however, part of the dye may diffuse (or be retained) in the cytosol. Permeabilization of the sarcolemma reduces the cytosolic rhod-2 fluorescence contribution, increasing the specificity of the signal, and allows a precise manipulation of the organelle environment (including the use of pharmacological inhibitors that otherwise would not cross the membrane barrier).

**Fig. 5:**
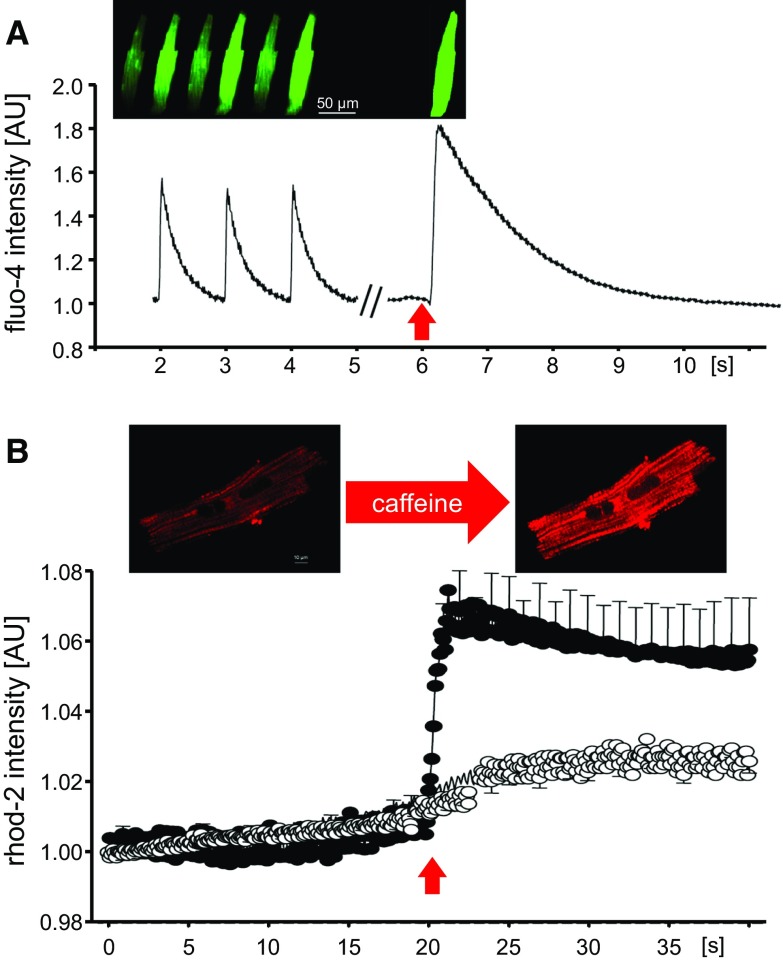
**a** Calcium transient recording in a fluo-4 loaded rat cardiomyocyte submitted to field stimulation (1 Hz, acquisition set at 40 images/s). After a period of stabilization, the cardiomyocyte is subjected to a single pulse of 10 mmol/L caffeine (red arrow) to induce maximal SR calcium release. The sequence of images shows diastolic and systolic calcium levels during pacing and after caffeine pulse. **b** Mitochondrial Ca^2+^ uptake throughout time in response to sarcoplasmic reticulum Ca^2+^ release (induced by a single pulse of 10 mmol/L caffeine, red arrow) in digitonin-permeabilized rhod-2 loaded mouse cardiomyocytes, under control conditions (black circles) or in the presence of 10 µmol/L of the mitochondrial calcium uniporter blocker Ru360 (white circles). Data correspond to mean ± standard error of the mean of *n* = 8 cardiomyocytes per group. Images correspond to a sarcolemmal-permeabilized cardiomyocyte loaded with rhod-2, before and after caffeine addition

Before permeabilization, cardiomyocytes are loaded with 4 μmol/L rhod-2 acetoxymethyl ester (previously prepared and aliquoted following manufacturer’s indications) using a cold–warm protocol, consisting of 60 min incubation at 4 °C followed by 30 min incubation at 37 °C. This double incubation enhances the retention of the dye within mitochondria. Alternatively, a simple warm protocol consisting of 50–60 min incubation with 4 µmol/L rhod-AM at 37 °C can be used when working with permeabilized cardiomyocytes, in which the cytosolic fluorescent contribution is negligible. Subsequently, cardiomyocytes are permeabilized and maintained in an intracellular-like buffer, as indicated above. When studying mitochondrial function, it is critical to work with healthy energized cells, because mitochondrial depolarization interferes with mitochondrial calcium uptake and retention of the fluorescent indicators. Changes in cytosolic calcium are induced by release of calcium from the sarcoplasmic reticulum through a caffeine pulse (10–20 mmol/L) while simultaneously monitoring changes in rhod-2 fluorescence throughout time with respect to the initial value (*F*/*F*_0_, excitation 561 nm/emission 605 nm). Permeabilized cells are more vulnerable to photobleaching and phototoxicity; therefore, it is important to quantify the contribution of photobleaching in control-permeabilized cells not submitted to any treatment and exposed to the same laser power (energy and speed of acquisition) as the treatment group (sham cells). When mitochondrial calcium is investigated, it is necessary to rule out the possibility that total sarcoplasmic reticulum calcium content is altered. To quantify total calcium load, intact (not permeabilized) cardiomyocytes are loaded with the cytosolic calcium indicator fluo-4 acetoxymethyl ester (5 μmol/L, 30 min at 37 °C), washed and post-incubated for 15 min at 37 °C. Cells are then placed on the stage of the microscope and submitted to a brief period of electrical pacing using field stimulation (0.5–1 Hz for 30 s) to allow calcium transient stabilization and appropriate replenishment of sarcoplasmic reticulum. During pacing, record calcium transients from fluo-4 fluorescence changes (excitation 488 nm/emission 520 nm). After a few transients, a single pulse of caffeine (10–20 mmol/L) is applied directly to the cells. The maximal amplitude of caffeine-induced fluo-4 fluorescence is normalized by the initial fluorescent value (*F*/*F*_0_); this value can be considered an index of total sarcoplasmic reticulum calcium load. Figure 5 shows an example of cytosolic calcium transients and maximal sarcoplasmic reticulum calcium release in intact cardiomyocytes (A) and the kinetics of mitochondrial calcium uptake in response to sarcoplasmic reticulum calcium release in permeabilized cardiomyocytes (B).

#### Permeability transition

Mitochondrial permeability transition has attracted much attention in the past decades as one of the critical determinants of cell death during ischemia/reperfusion [167, 174]. Sustained mPTP opening results in mitochondrial matrix swelling and energetic collapse that is incompatible with cell life. Its contribution to necrotic death during reperfusion has been unambiguously demonstrated in multiple studies [82, 174, 183, 224]. Moreover, several lines of evidence suggest that the infarct sparing effect of ischemic pre- and postconditioning is at least in part mediated by the prevention of mPTP opening [179, 324]. Although the exact molecular entity of mPTP remains debated, its opening is triggered by well-characterized factors, among which increased mitochondrial calcium, ROS and normalization of intracellular pH are of main importance [31].

Various methods are available for the direct evaluation of mitochondrial permeability transition in different experimental models, including primary cells (see above). Whatever method is used or consequence is detected, mPTP involvement is usually confirmed by the inhibitory effect of CsA. However, this approach can easily generate both false-positive and -negative results. First, mPTP-independent effects could be elicited, since CsA binds all the cyclophilins in addition to the mitochondrial cyclophilin-D. This is the case with calcineurin inhibition followed by a decreased occurrence of mitochondrial fission [68]. Second, cyclophilin-D is only a mPTP modulator; thus mPTP opening could still occur also in the presence of CsA. Thus, the absence of a CsA effect is not sufficient to rule out the involvement of mPTP opening in a given process [39].

##### Calcein release

The calcium retention capacity assay can be used in isolated cells following permeabilization with saponin. Nevertheless, it cannot be used in intact cells and monitoring mitochondrial swelling requires a complex procedure of confocal microscopy [232]. The reference method for detecting mPTP opening in intact cells is based upon the intracellular redistribution of calcein coupled with Co^2+^ quenching of cytosolic calcein fluorescence [338]. In particular, cells are loaded for 15 min with 1 mmol/L calcein acetoxymethyl and 1 mmol/L CoCl_2_ at 37 °C. Cells are then washed free of calcein and CoCl_2_, and the experiment begins by controlling the attainment of quenching of the cytosolic fluorescence signal resulting in the visualization of bright mitochondria glowing against a dark background.

**Fig. 6:**
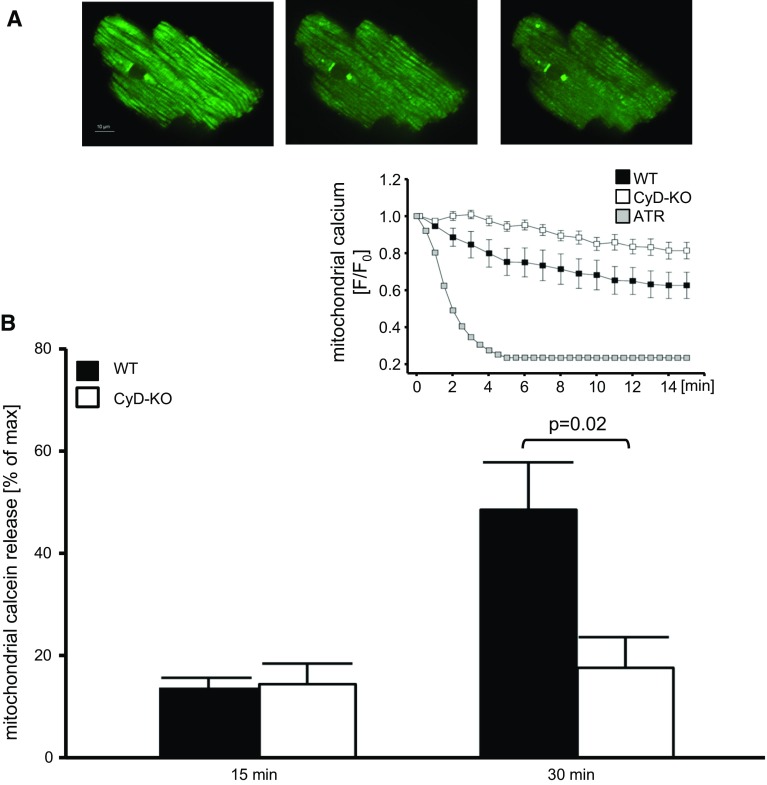
**a** Sequence of a calcein-loaded mouse cardiomyocyte with rigor contracture during the first 5 min of reperfusion (60 × magnification). Because calcein is entrapped in mitochondria, the decay of green fluorescence reflects mitochondrial permeabilization. **b** Quantification of total mitochondrial calcein release at 15 min reperfusion, with respect to maximal mitochondrial calcein release achieved after the addition of atractyloside (ATR), in isolated cardiomyocytes from wild-type (WT) and cyclophilin-D knock-out (CyD-KO) mouse hearts, previously submitted to either 15 or 25 min ischemia. The inset shows mitochondrial calcein kinetics [changes in fluorescence with respect to the initial value, *F*/*F*_0_)] throughout reperfusion, indicating mitochondrial permeability transition pore (mPTP) opening in the different groups of cells. Maximal mitochondrial calcein release was induced in WT cardiomyocytes treated with 20 µmol/L atractyloside. Data are expressed as mean ± SEM of 5–6 cardiomyocytes per group. Modified from [358]

Opening of mPTP is detected as a decrease in calcein fluorescence using a confocal or epifluorescent microscope (excitation 488 nm/emission 525 nm). To semi-quantitatively calibrate the signal, maximal calcein release can be pharmacologically induced at the end of the experiment by addition of 30 µmol/L alamethicin. Calcein release kinetics may be automatically quantified with the aid of imaging software and expressed as percent of calcein fluorescence decrease with respect to maximal calcein release after atractyloside treatment. Alternatively, calcein release can be normalized with respect to the initial fluorescent value (*F*/*F*_0_), although in this case, it is recommended to quantify the contribution of photobleaching to the total fluorescence decay. Also, to avoid the occurrence of reperfusion-induced hypercontraction that interferes with calcein quantification, a concentration of 5 mmol/L 2,3-butanedione monoxime can be added to the reperfusion buffer. Monitoring of mitochondrial calcein release reflects mPTP opening in cardiomyocytes submitted to ischemia/reperfusion (or other pathological conditions) and its occurrence usually correlates with the severity of cell damage, although the mechanisms of damage may vary among the specific experimental context and conditions (degree of calcium overload, kinetics of pH normalization, severity of mitochondrial dysfunction). Figure 6 shows an example of mitochondrial calcein release and quantification in cardiomyocytes subjected to ischemia/reperfusion.

#### Mitochondrial reactive oxygen species

In the past decade, several probes have been developed to measure intracellular and compartmentalized ROS formation inside the cell [110, 237, 457]. Small molecule fluorescent dyes, such as MitoSOX and reduced MitoTracker dyes, are commonly used for the detection of mitochondrial ROS formation in intact cells.

MitoSOX Red is a derivative of hydroethidine and is widely used for the measurement of O_2_^−^ formation in active mitochondria. This dye is specifically targeted to mitochondria because it contains the lipophilic cation triphenyl phosphonium substituent [88, 237]. MitoSOX Red is oxidized by O_2_^−^ to form a red fluorescent product 2-hydroxyethidium, which is excited at 510 nm and emits at 580 nm [88]. Even though MitoSOX is used to specifically detect O_2_^−^ formation, this probe can also be oxidized by other oxidants to form ethidium, which overlaps with the fluorescence peak of 2-hydroxyethidium [238, 463]. Moreover, at high concentrations, MitoSOX displays some non-mitochondrial staining, for instance, in the nucleus [351].

MitoTracker Orange CM-H_2_TMRos and MitoTracker Red CM-H_2_XRos are derivatives of dihydrotetramethyl rosamine and dihydro-X-rosamine, respectively [237]. Reduced MitoTracker dyes do not fluoresce until entering viable cells, where they get oxidized and become positively charged. The cationic fluorescent compound then accumulates in the mitochondria depending on ROS levels and mitochondrial membrane potential, and forms fluorescent conjugates with thiol groups [215]. The excitation/emission wavelengths for MitoTracker Orange CM-H_2_TMRos and MitoTracker Red CM-H_2_XRos are 554/576 and 579/599 nm, respectively [237]. Unlike MitoSOX, these reduced MitoTracker dyes are not specific for single oxidant species and thus detect general mitochondrial ROS [237]. Moreover, the fact that their mitochondrial localization and accumulation depend on the mitochondrial membrane potential is a crucial aspect to bear in mind for the correct interpretation of the fluorescence intensity levels [351, 463]. Indeed, it is necessary to measure mitochondrial membrane potential to avoid erroneous interpretations.

##### Use of genetically encoded fluorescent probes, exemplified by HyPer

As described above, fluorescent redox probes such as MitoSOX and MitoTracker dyes present limitations in terms of selectivity, sensitivity and localization. Therefore, genetically encoded biosensors have been developed to measure H_2_O_2_ or other species fluctuations in different compartments of living cells, such as reduction–oxidation-sensitive green fluorescent protein [114], Orp1, [11] and HyPer (Hydrogen Peroxide sensor) [114].

HyPer contains a H_2_O_2_-sensitive regulatory domain of E. coli transcription factor OxyR [36, 79], bound to a circularly permuted yellow fluorescent protein (cpYFP). OxyR can be oxidized by H_2_O_2_, leading to the formation of a disulfide bond between Cys-199 and Cys-208; this oxidative modification can be reversed by the activity of endogenous glutaredoxins. Oxidation-induced conformational change is transmitted to cpYFP that is integrated into the conformation-changing region in OxyR between residues 205 and 206 [79]. Therefore, H_2_O_2_-induced oxidation of OxyR is reflected by changes in fluorescence properties of cpYFP. In particular, a decrease in the 420-nm peak is coupled with a proportional increase in the 500-nm peak, making the sensor ratiometric [287].

The major advantages of this genetically encoded redox sensor are that it is ratiometric, reversible and can be targeted to specific compartments of the cell. Several variants of HyPer are commercially available, such as cytosolic (pHyPer-cyto), mitochondrial (pHyPer-dMito) and nuclear (pHyPer-nuc) constructs. In addition, other versions of the plasmid targeted to endoplasmic reticulum, lysosomes and different compartments within the mitochondria have been reported. Thus, HyPer is a useful tool to measure compartmentalized H_2_O_2_ formation [293].

HyPer has two excitation maxima (420/500 nm) and a single emission peak maximum (516 nm). Upon oxidation, the intensity of the 420-nm peak decreases proportionally to the increase of the intensity of the 500-nm peak, thus making HyPer a ratiometric sensor. An increase in H_2_O_2_ levels is directly proportional to the increase in fluorescence ratio *F*_500_/*F*_420_ [44].

Even though HyPer is widely used to detect H_2_O_2_, its fluorescence levels can be influenced by pH, potentially leading to erroneous result interpretation. Hence, it is important to monitor pH in the same compartment and experimental conditions in which HyPer is being used. This can be accomplished using SypHer, a form of HyPer bearing a mutation in one of the two H_2_O_2_-sensing cysteines of the OxyR domain, making it a H_2_O_2_ insensitive but pH-sensitive sensor [237].

### Major methods for evaluating mitochondrial function in vitro

#### Oxygen consumption

Oxygen consumption is usually measured by means of an oxygen electrode put at the bottom of glass chambers with sizes ranging from few microliters to 2 mL. The measurement of respiration states along with the specific evaluation of the activity of respiratory complexes can be obtained only in isolated mitochondria or permeabilized cells. Mitochondria are isolated by means of enzymatic and/or mechanical cell disruption followed by conventional procedures of differential centrifugation [106, 141, 278]. Permeabilization, i.e. loss of sarcolemmal integrity, can be obtained by adding 10 µmol/L saponin to cells incubated in the same buffer used for assessing respiration (125 mmol/L sucrose, 65–125 mmol/L KCl, 20 mmol/L HEPES, 1 mmol/L MgCl_2_, 100–200 µmol/L EGTA, 2.5 mmol/L KH_2_PO_4_, pH 7.4). Isolated mitochondria or permeabilized cells are added to the respiration chamber to achieve a final concentration of 0.1–0.2 mg protein/mL in the case of cardiac samples. Basal (state 4) respiration is monitored following addition of respiration substrates, 5/2.5–5 mmol/L glutamate/malate (complex I) or 5 mmol/L succinate (complex II) in the presence of 2 µmol/L rotenone. After 3 min, 0.6 mmol/L ADP is added to induce state 3 respiration. The ratio between state 3 and state 4 respiration, termed respiratory control index, is a reliable marker of the quality of the mitochondrial preparation. Values should be > 6 and > 3 with NAD-dependent and flavin adenine dinucleotide-dependent substrates, respectively. In a separate assay, or after the return to state 4 respiration, mitochondrial uncoupling can be obtained by adding (sub)micromolar amounts of FCCP, determined upon titration. This procedure is necessary to assess the maximal rate of respiration independently of its coupling with ATP synthesis. Indeed, a reduced state 3 rate could be affected by alterations in the activity of FoF1 ATPase and/or adenine nucleotide translocase, while uncoupled respiration depends only on respiratory chain complexes. Data are expressed as the rate of oxygen consumption normalized to mg of mitochondrial proteins in the case of isolated organelles or number of cells in the case of cell permeabilization.

#### Mitochondrial swelling

Mitochondrial swelling is monitored as the changes in absorbance at 540 nm. Incubations are carried out with 0.25 mg of mitochondrial protein/mL in a respiration buffer containing 0.25 mol/L sucrose, 10 mmol/L Tris-Mops, 0.05 mmol/L EGTA, pH 7.4, 5 mmol/L pyruvate, 5 mmol/L malate, and 1 mmol/L Pi-Tris, pH 7.4. Alternatively, a KCl-based buffer can be used containing 137 mmol/L KCl, 2 mmol/L KH_2_PO_4_, 20 mmol/L HEPES, and 20 μM EGTA, pH7.4, with pyruvate/malate or glutamate/malate as the respiratory substrates. mPTP opening is induced by the addition of 0.25 mmol/L CaCl_2_ [107]. Please note that variations of this general protocol can be used for the study of mPTP opening in de-energized mitochondria [19].

#### Calcium retention capacity

The assay measures the amount of Ca^2+^ that mitochondria can accumulate and retain before the precipitous release that marks mPTP opening [140]. Extra-mitochondrial Ca^2+^ fluxes are measured fluorometrically using Calcium Green-5N that increases its fluorescence emission upon Ca^2+^ binding. Mitochondria (0.1–0.25 mg/mL) are suspended in 200 μL of respiration buffer that also contains 1 μM Calcium Green-5N but no EGTA. The suspension is subjected to a train of Ca^2+^ pulses or spikes (typically 10 μM each). Each spike represents the increase in fluorescence (due to extra-mitochondrial Ca^2+^ binding to Calcium Green- 5N) followed by a decrease in fluorescence due to the accumulation of added Ca^2+^ into mitochondria. Mitochondrial accumulation of Ca^2+^ continues upon addition of subsequent pulses until the “threshold” for mPTP activation is reached and all the Ca^2+^ is released from mitochondria (represented by the dramatic terminal fluorescence increase) due to the opening of the mPTP. Dose–response curves are calculated by comparing the total Ca^2+^ added for mPTP activation in the presence of a test compound with respect to the calcium retention capacity in the absence of test compound.

#### Measurement of reactive oxygen species by Amplex Red

The Amplex Red assay is a widely used procedure for assessing H_2_O_2_ formation in cell-free extracts, including mitochondria [237]. This technique is devoid of limitations affecting other assays that are frequently used. Among these, the fluorescence of 2′,7′-dichlorofluorescein is hampered by its low specificity for H_2_O_2_ [65] and susceptibility to oxidation by iron or cytochrome c [238]. Amplex Red is a substrate of horseradish peroxidase, which is oxidized in the presence of H_2_O_2_ resulting in the production of a red fluorescent compound resorufin (excitation/emission: 540 or 571/580 or 585 nm) [402]. Amplex Red reacts with H_2_O_2_ in a 1:1 stoichiometry to produce resorufin. The major shortcoming is the significant light sensitivity of Amplex Red that can lead to the formation of resorufin even in the absence of horseradish peroxidase and H_2_O_2_ [459]. Thus, necessary precautions should be taken to prevent photooxidation of this probe. Moreover, the fact that Amplex Red is cell impermeable limits its use to the permeabilized cells [402].

Amplex Red assay is commonly used to measure ROS produced by the mitochondrial respiratory chain in the presence of substrates, such as succinate or glutamate/malate. Moreover, it is also possible to measure the activity of several enzymes that produce H_2_O_2_, including monoamine oxidases. These mitochondrial flavoenzymes catalyze the oxidative deamination of catecholamines and biogenic amines, resulting in the production of H_2_O_2_, aldehydes and ammonia [236].

### Measurement of mPTP opening in whole hearts

In the procedure devised in Halestrap’s laboratory, perfused hearts are first loaded with [^3^H] 2-deoxyglucose, which accumulates within the cytosol as [^3^H]-deoxyglucose-6-phosphate, but can only enter the mitochondria when the mPTP opens. Using this approach, it has been shown that mPTP remains closed during ischemia but opens upon reperfusion [80]. A similar conclusion has also been obtained using a procedure based on the release of mitochondrial NAD^+^ upon mPTP opening [82]. Besides representing a useful analytical tool, the loss of mitochondrial NAD^+^ provides additional pathways linking mPTP opening to cell death [83].

### Issues and limitations in evaluating mitochondrial parameters

Besides limitations associated with experimental models, one cannot be too cautious in interpreting results of mitochondrial dysfunction as evidence of cardiac injury or protection. The following list summarizes the major issues, as well as common misinterpretations.

1. Mitochondrial dysfunction is not necessarily related to cell death, since it is involved in many forms of cardioprotection.
2. The assessment of functional parameters in mitochondria isolated from a given cell or tissue exposed to injury does not provide reliable information on the function of mitochondria in situ and their contribution to loss or maintenance of viability. Indeed, the quality of the mitochondrial preparation is determined by the quality of the starting material. Invariably, a damaged heart yields a mitochondrial fraction with decreased function without indicating whether mitochondrial dysfunction is causally related to heart/cardiomyocyte derangements in situ.
3. Mitochondrial function or structure should not be evaluated as a single endpoint at the end of a given protocol. For instance, mitochondrial derangements are expected to occur at the end of reperfusion, but this kind of observation does not determine whether mitochondrial abnormalities are a cause or a consequence of reperfusion injury.
4. The observation that a given mitochondrial component is up- or down-regulated is hardly informative on mechanisms causing cardiomyocyte injury or protection. Mechanistic information should be obtained by demonstrating that the absence of that mitochondrial component or process modifies the extent of injury (or protection).
5. The qualitative assessment of mitochondrial changes is hardly predictive of cellular outcome and does not allow the elucidation of detrimental or beneficial pathways. This is especially the case with changes in levels of Ca^2+^ and ROS that appear to be involved in both injury and protection. What is lacking is the knowledge of a precise threshold separating protective from deleterious levels.
6. Mitochondrial changes associated with protection against acute injury, such as the classic ischemia/reperfusion model, are frequently detrimental in chronic settings. Besides the extreme (and seminal) examples of cardioprotection elicited by cyanide or mitochondrial uncouplers [123, 145], cardiac alterations have been reported to result from long to lasting mPTP inhibition [122]. Furthermore, cardiac function is inevitably hampered by respiratory chain inhibition that protects against acute ischemia/reperfusion protocols [69, 80].

## Cell types other than cardiomyocytes

### HL-1 cardiomyocytes

Immortalized cardiomyocytes, which proliferate in culture conditions and maintain their fully differentiated phenotype, do not exist. In the last decades, different attempts to create such cell models have been made with little success. Technical approaches have been based on the generation of transgenic mice that contained an oncogene, the expression of which was targeted to the myocardium using different cardiac-specific promoters [100, 245]. In 1998, Claycomb et al. developed the commercially available HL-1 cell line [83], which was established from an atrial subcutaneous tumor excised from an adult female C57BL/6J mouse. HL-1 cells can grow in culture for long-time and display a myofibrillar organization similar to the mitotic cardiomyocytes of the developing heart. They form cell-to-cell contacts through gap junction-containing intercalated disks and, after some days in the appropriate culture conditions, they exhibit spontaneous contractile activity that tends to synchronize in the form of clusters of contracting cells. Culturing of HL-1 cells is relatively easy. With the usual caution and sterility required for the management of cells, HL-1 cells can be efficiently passaged and expanded. They can also be frozen and recovered from frozen stocks. Nevertheless, long-term cultivation of HL-1 cells is expensive, because it requires the use of a very specific medium (Claycomb medium), the composition of which is protected under patent. Despite some limitations, i.e. progressive loss of contractile activity after several passages by not well-controlled mechanisms, the HL-1 cell line is a widely accepted model for basic research studies on cardiac pathophysiology [137], cell signaling [453], cardioprotection [357], electrophysiology [379] and pharmacology [159]. Indeed, HL-1 cells express important proteins of the adult heart (e.g. connexin 43, ANT, alpha isoform of the myosin heavy chain), preserve an adult pattern of sarcomeric genes, maintain several voltage-dependent electrical currents and exhibit cyclic calcium movements [83]. In addition, HL-1 cells are a suitable model for studies with gene transfection and protein overexpression [50]. However, of note, they do not represent true adult cardiomyocytes but rather a hybrid stage between embryonic and postmitotic cells. In contrast to adult cardiomyocytes, the energy metabolism of HL-1 cells relies on glycolytic activity [308]. Because HL-1 are atrial-derived cells, they may not share the functional traits of ventricular cardiomyocytes.

### H9c2 cells

H9c2 cells are a subclone of the original cell line derived from embryonic BD1X rat heart tissue [251], which proliferates in culture for a limited number of passages on a Dulbecco’s modified Eagle’s medium. H9c2 myoblasts have ventricular origin but share some properties with skeletal muscle. Indeed, they have the ability to transdifferentiate into multinucleated skeletal myotubes with very low proliferative capacity when serum is reduced to 1% concentration. In addition, when subjected to chronic treatment (5–6 days) with all-trans-retinoic acid (100 nmol/L^–1^ µmol/L), H9c2 may undergo an additional differentiation step into a cardiac-like phenotype [298]. Depending on their differentiation stage, H9c2 cells predominantly exhibit cardiac-specific voltage-dependent calcium channels, skeletal voltage-dependent calcium channels or a mixture of both [194, 298]. It is, therefore, of critical importance to establish the appropriate differentiation phenotype before planning experiments. In the fully cardiac differentiated stage, H9c2 cells display an elongated morphology and express sarcomeric proteins as well as proteins involved in calcium handling which are typical of the adult cardiomyocyte, including sarcoplasmic reticulum calcium ATPase 2, ryanodine receptor and phospholamban [59]. They also adopt a more oxidative metabolism, characterized by good coupling between glycolysis and mitochondrial respiration and indicative of higher energetic efficiency [336]. Different reports agree that cardiac-like H9c2 cells resemble more closely adult cardiomyocytes than HL-1 cells in terms of their metabolic signature, mitochondrial respiratory function and vulnerability to ischemia/reperfusion injury [2, 265, 314].

### Stem cells

Physiological experiments requiring cardiomyocytes are still limited by the fact that adult, terminally differentiated cardiomyocytes are mostly isolated from rodents which may differ from human cardiomyocytes, by the need of fresh isolation, and by their limited cell number. The use of stem cells may be regarded as an alternative strategy. The use of stem cells from various origins has become popular in regenerative medicine. The advantages and limitations of such protocols have recently been discussed in detail [291]. With respect to experimental studies, the most successful strategy is the use of human pluripotent stem cells (PSC) which routinely differentiate into cardiomyocytes by more than 80% [45]. However, the remaining cultures are still a mixture of atrial, ventricular, and nodal cells with different phenotypes and they represent a spontaneously beating culture which differs from native differentiated cardiomyocytes. Such cell culture models may be of specific advantage to identify the paracrine potential in myocardial infarction [290]. Similarly, embryonic stem cells have been used to study potential cytoprotective effects. The longevity of these cells under culture conditions can be prolonged by administration of NO [162, 163, 328].

### Endothelial cells

Cardiac endothelial cells are less susceptible than cardiomyocytes to oxygen deprivation but may develop hypercontracture, necrosis and apoptosis during reperfusion. In contrast to cardiomyocytes, endothelial injury may affect cardiac function by mechanisms other than the death of endothelial cells, including increased permeability and edema, and recruitment of platelets and inflammatory cells [407].

Endothelial cell necrosis can be detected in cell cultures with the same techniques used in cardiomyocytes, except for the absence of gross morphological changes, as hypercontracture of endothelial cells is much more subtle than that of cardiomyocytes.

There is strong evidence that endothelial cells may develop apoptosis during initial reperfusion. In cell cultures, apoptosis can be detected by a large variety of methods, including the quantification of cell membrane proteins, in particular those which are externalized early during the apoptosis process, detection of DNA fragmentation, or analysis of morphological changes in cell nuclei (for detailed protocols see [91]).

Apoptosis-induced translocation of the phospholipid phosphatidyl serine is easily detected with fluorescein-labeled annexin V (FICT-Fluorescein V). Annexin V labeling occurs before DNA fragmentation and persists until the end of the apoptotic process, but also occurs during necrosis, as permeabilization of the plasma membrane allows annexin V to reach intracellular phosphatidyl serine. To distinguish apoptotic from necrotic cells, a double labeling with annexin and propidium iodide is usually performed. Cells labeled with annexin V but not with propidium iodide are considered apoptotic, while those labeled with both are classified as necrotic. Detection and quantification of fluorescence can be performed by conventional or confocal fluorescence microscopy or by flow cytometry analysis.

Detection of apoptosis-induced DNA fragmentation may be carried out by different forms of electrophoresis and by sophisticated labeling methods in cell preparations but is less sensitive and less practical that Annexin V-based methods [18].

In tissue, detection of DNA fragmentation through electrophoresis does not allow identification of which types of cells are undergoing apoptosis. Histologic analysis of fixed tissue after staining with hematoxylin–eosin or other methods allows morphological identification of apoptotic cells, but the sensitivity, reproducibility and quantitative information provided by this method is limited.

## Quantitation of infarct size by morphology, biomarkers and imaging

Measuring the size of the infarct is essential for all cardioprotection studies. Quantification can be achieved by different techniques but they all carry specific concerns. Consequently, it is important to be aware of conceptual limitations of the measurement of infarct size that are common to all techniques.

### Common key issues for infarct size measurement

#### Why measure infarct size?

The measurement of infarct size is a crucial issue of cardioprotection, since the amount of irreversible myocardial damage influences left ventricular remodeling, recovery of contractile function, and patient’s prognosis. It has then naturally become the common way to evaluate and compare the beneficial effects of potential protective therapies, and is currently the endpoint of most experimental studies as well as phase II clinical studies in the field of ischemia/reperfusion [184]. This consensus should, however, not mask difficulties. Assessing the size of an infarct is not limited to technical questions, but first and foremost requires a good knowledge of some conceptual limitations and pitfalls of interpretation.

#### Infarct size according to models

##### Animal models

Progress in understanding the pathophysiology of ischemia/reperfusion comes largely from seminal studies using transient coronary occlusion in large animal models, particularly anesthetized dogs [348]. Infarcts were often large, transmural, corresponding to what was observed clinically at the time due to absent or late reperfusion. As it should be, these authors expressed the size of the infarct in grams of myocardial tissue irreversibly injured. Global ischemia in the rat isolated perfused heart has also been a very useful model for understanding the pathophysiology of ischemia/reperfusion especially with respect to its metabolic aspects. Nevertheless, the pathophysiology of cell death is different from that in in vivo models, and extrapolation from one model to the other of data regarding the beneficial effects of given treatment should be made with caution.

##### Cell death after hypoxia reoxygenation

Cell cultures and isolated cells are widely used for pathophysiological studies but also for testing pharmacological agents [326]. These experimental conditions are obviously very different from those of in vivo models, because of lack of contractile activity and the absence of neighboring cells such as fibroblasts, vascular cells and nerves. Importantly, quantitation of the number (or percentage) of dying cells after hypoxia and re-oxygenation is not the equivalent of measurement of infarct size. Consequently, observing a decrease in viability following any treatment does not presume an equivalent reduction in infarct size after the same therapeutic intervention in an in vivo model.

##### Clinical settings

The average infarct size observed nowadays in STEMI patients has been considerably reduced by shortened delay to PPCI and the development of interventional cardiology techniques. While smaller infarcts are much more frequent, their impact on left ventricular remodeling, recovery of contractile function, heart failure and mortality is less clear than for a large STEMI. Unfortunately, the size of the infarct is not even measured in many interventional cardiology centers even though routine biomarkers for that measurement are easily available and inexpensive. Although infarct size is recognized as a major predictor of prognosis, all patients with acute myocardial infarction patients usually receive the same treatment at discharge regardless of the amount of irreversible damage to the heart.

In contrast, peri-operative and peri-procedural myocardial damage is assessed in clinical settings such as cardiac surgery or endovascular procedures for chronic coronary or even valvular diseases [74, 193, 234, 330, 422]. Here, however, tissue destruction is fortunately quite minimal and its impact on ventricular function and clinical outcomes remains less clearly defined and in any case not comparable to that in STEMI. As a matter of fact, reducing by 40% a troponin release peak of 6 μg/L does not have the same meaning as reducing by 40% a troponin release peak of 150 μg/L [74, 340]. This identical use of the measurement of infarct size in STEMI and experimental conditions where infarction is very small deserves significant caution. As for cardioprotection, this use of infarct size measurement may have been misleading and in part explains some of the “failures” of the transfer to the clinic of certain cardioprotective interventions [182, 302].

#### Measuring reperfusion infarction: a critical unsolved issue

Most of the cardioprotection studies aim to reduce infarct size through new therapies administered at the time of PPCI, i.e. mainly targeting reperfusion injury. Unfortunately, we do not know how to specifically measure reperfusion lesions, i.e. to distinguish them from ischemic lesions: we measure a final infarct size which encompasses ischemic plus reperfusion injuries. The size of the reperfusion injury cannot be measured in a given individual but may only be averaged by comparing the mean infarct size in a treated group compared to that in a control group. Imaging of the infarct size before PPCI and following its extension after reflow would be the only way to address this question. For the moment, this poses unresolved technical problems and risks delaying reperfusion by having to image before coronary recanalization. Such developments, including new imaging methodologies such as computed tomography and cardiac magnetic resonance imaging (CMR) in the catheterization laboratory, are currently being studied.

#### Infarct size as a function of area at risk

The experimental studies have clearly shown that the final size of the infarct is determined mainly by the duration of ischemia, the size of the area at risk and the amount of residual myocardial flow in the ischemic bed [184]. Any measurement of infarct size should, therefore, be accompanied by a concomitant measurement of these determinants. All experimental models use a fixed duration of ischemia. Collateral flow may be measured using labeled microspheres; alternatively, one may choose an experimental model with little or no collateral circulation (e.g. rat, rabbit, pig).

While the measurement of the area at risk is well defined in experimental models (see below), it is much more difficult in clinical conditions. IV administration of ^99^Technetium sestamibi while the artery is still occluded allows a reliable assessment of the hypo-perfused area if single-photon emission computed tomography (SPECT) is completed within 6 h after injection. Unfortunately, it is often not compatible with emergency care because ^99^Technetium sestamibi must be prepared within a few hours preceding the SPECT imaging [20].

CMR is the most commonly used technique to measure both the area at risk and infarct size. T2-weighted CMR (T2w-CMR) allows the evaluation of the size of the area at risk. It does not measure tissue perfusion during the ischemic period but detects myocardial edema which develops particularly after reflow [301]. Experimental evidence suggests that the post-ischemic edematous zone matches the area at risk [12]. This well-standardized method nevertheless poses several major problems [210]. First, the edema area is indeed wider than the area of hypoperfusion during the ischemic phase. Second, the dynamics of the myocardial edema are unpredictable in a given patient and there seems to exist significant variation in tissue water content between day 1 and day 7 after reflow [131, 132]. Third, treatments that reduce infarct size also attenuate myocardial edema [423]. Then, the size of the area at risk measured in patients who received a cardioprotective therapy will appear smaller than the “real” area at risk, which is obviously a bias when compared with an untreated group. Altogether, T2w-CMR should not be used to assess the size of the area at risk in cardioprotection studies [89]. In contrast, CMR evaluation of infarct size by late gadolinium enhancement (LGE–CMR) (see below) is reliable and relatively simple, and is currently considered as the standard technique for measuring infarct size (expressed in grams of myocardium) in patients. Unfortunately, only a minority of interventional cardiology centers are capable of performing quality CMR evaluation of infarct size 24 h every day. Other modes of CMR imaging analysis are under development that might improve its performance.

### Reference techniques

#### Histology

For in vivo models, the recommended technique is the blue dye coloration for delineating the area at risk and the identification of viable myocardium by 2,3,5-triphenyl tetrazolium chloride (TTC) staining. At the end of the reperfusion period, the coronary artery is briefly re-occluded and 0.5 mg/kg Evans blue is injected intravenously to delineate the left ventricular area at risk [439]. The left ventricle is then excised and cut into five or six transverse slices. Each slice is then incubated in TTC at 37 °C to differentiate infarcted (pale) from viable (brick red) myocardial area. Slices are weighed and photographs of the proximal side of each slice are taken. Online software (e.g. Image *J*) allows measurement of surface areas of the whole slice, the risk region, and the necrotic area. For each slice, multiplication of the fraction of the slice’s surface area that is TTC negative by the slice weight results in the amount of infarcted tissue in grams. Addition of infarct sizes of each slice results in the total amount of infarcted tissue in grams for a given heart. Identification of necrotic vs. viable myocardium by TTC is not always easy due to failure of well-contrasted coloration. Therefore, analysis must be blinded and consistent (by the same investigator). In case of poor staining, the heart should be discarded.

#### Imaging

CMR assessment of infarct size should be performed 3–5 days after PPCI; for consistency, it is recommended to perform the examination always at the same time delay (e.g. 2 days) after PPCI. However, if the decision is made not to measure the edema-based area at risk, another strategy would be to measure infarct size (only), for example, at 1 month after PPCI, when most of the acute edema has disappeared. The inconvenience is that the patient must come back.

When eventually measured (but not recommended, as indicated above), T2w-CMR imaging is based on breath-hold T2w-short tau inversion recovery sequences covering the whole left ventricle [300]. The T2w-CMR hyper-intense area is quantified on the T2w-CMR images using semi-automatic detection with the full width at half-maximum approach. The myocardial edema area is quantified in each short-axis slice. The extent of myocardial edema is expressed as the indexed mass of the myocardial edema (in grams of tissue) according to the following formula: [hyperenhanced area (in cm^2^)] × slice thickness (in cm) × myocardial specific density (1.05 g/cm^3^)/body surface area (m^2^).

Infarct size is assessed as the area of hyper-enhancement on the LGE–CMR images. Delayed hyper-enhancement is evaluated 10 min after the injection of gadolinium (0.2 mmol/kg at 3 mL/s) with the use of a three-dimensional inversion-recovery gradient-echo sequence. The area of hyper-enhancement is expressed as the indexed mass of the infarcted myocardium.

#### Biomarkers

The release of ischemic biomarkers, mainly serum creatine kinase (CK) and troponins, has long been used for confirming the diagnosis of acute myocardial infarction. The amount of enzymes released into the blood circulation may be evaluated either at a single time point (usually the estimated peak of the release kinetics) or by the area under the curve of several measurement points [417]. In both cases, the value obtained is often considered as measuring the size of the infarct.

For example, in several phase two studies, blood samples were taken at admission, every 6 h after opening of the coronary artery during day 1, and every 6 h on days 2 and 3. The area under the curve (arbitrary units) of CK release (Beckman Kit, expressed in IU/L) was measured in each patient by computerized planimetry (Image *J* 1.29x) and used as a surrogate marker of infarct size [401, 419]. A similar approach can be used for TnI, although care should be taken that different assay kits yield discordant values. On the other hand, the CK assay is robust and in particular correlates well with the measurement of CMR infarction. However, whereas the CK kinetics are clear in patients with TIMI flow grade 0–1 on admission with an early peak near 6–8 h post-PPCI and return to near baseline values in about 72 h, the release profile may be significantly altered (e.g. no identifiable peak) in patients with TIMI flow > 1, in patients with cardiopulmonary arrest who underwent defibrillation, or in patients revascularized by thrombolysis. Above all, the interpretation of the infarct size and the effect on it by a therapeutic intervention must be made cautiously. One does not know the equivalence in grams of necrotic myocardial tissue of a given quantity of cardiac enzymes released into the blood. In other words, the CK or TnI value is not an infarct size. A percentage reduction in the CK/troponin peak or area under the curve following administration of a supposedly protective treatment should not be interpreted as a percentage reduction in infarct size. For example, in the study by Piot et al. CsA reduced peak CK release by 36% which corresponded to a reduction of 20% in infarct size as measured by LGE–CMR [340]. Hence, cardiac enzyme release is a robust method, the interpretation of which must be made with caution, in particular by assessment of the potential benefit of an infarct-size reducing drug.

### Practical summary: your best way to measure infarct size

Several reliable techniques are available to measure infarct size in various experimental models and in patients. Recommendations are: (1) to make sure that what is measured is really an infarct size, (2) to be aware of the technical limitations and difficulties of interpretation of the results, (3) to choose a largely available and well-validated method with respect to the experimental model, (4) to use largely accessible techniques in patients.

## Isolated perfused hearts

The method of retrograde mammalian heart perfusion was developed by Oscar Langendorff in 1895 [269]. It provides the ability to study the function of the heart in isolation from the rest of the body and nervous system, while retaining the advantage of studying cardiac cells in their native myocardial structure and environment. More detailed discussions of the rationale behind the model as described below can be found in reviews [35].

### Species and strain

Any mammalian heart can be perfused using the Langendorff technique, and hearts from mice, rats, guinea pigs, rabbits, dogs, primates and even humans have been studied [233], although their sensitivity to ischemic injury and responsiveness to myocardial protection varies [144]. These days, however, most experimentalists use rats or mice. Rat hearts have the advantage of being slightly larger, rendering the left anterior descending coronary artery (LAD) more accessible and hence easier to tie off, and infarct size determination is somewhat more accurate. The main advantage of mouse hearts is that it allows the use of genetically modified strains. Animals are usually 10–12 weeks old and 200–300 g (rats) or 8–12 weeks and ~ 20 g (mice). Older animals may have additional fat around the heart which may complicate harvesting.

The most commonly used rat strains are Sprague–Dawley, Lewis, and Wistar, generally for reasons of practicality or convenience. These strains are all outbred (i.e. a closed population of genetically variable animals that is bred to maintain maximum heterozygosity), which means that strains bred in different places may be slightly different due to genetic drift. Other strains of rats can also be used—in fact Fischer inbred rats may be advantageous as they have been reported as having a less variable coronary architecture [282]. Resistance to myocardial ischemia differs between different inbred strains of rats [28].

The most commonly used mouse strain for routine experiments is C57Bl/6. These are inbred and, therefore, genetically nearly identical. However, there are several different sub-strains of C57Bl/6, notably C57Bl/6J and C57Bl/6N which have significant phenotypic differences including their response to ischemia [320]. Therefore, the precise strain and its origin should be reported for all studies. There is wide inter-strain variability in sensitivity to ischemia/reperfusion [171]. Importantly, however, a reduction of infarct size by direct and remote ischemic preconditioning has been reliably demonstrated in all strains of mice tested [61]. Some transgenic or knockout strains may be on other backgrounds, in which case it is essential to use the same precise strain as the mutated mice for the WT controls, and preferably littermates [164].

Historically, most experiments have been performed using only male animals, as this is believed to reduce potential variability due to hormonal influences. However, the importance of studying the response of both male and female hearts is becoming clear, as sex can clearly influence sensitivity to ischemia [47, 323].

### The Langendorff apparatus

**Fig. 7:**
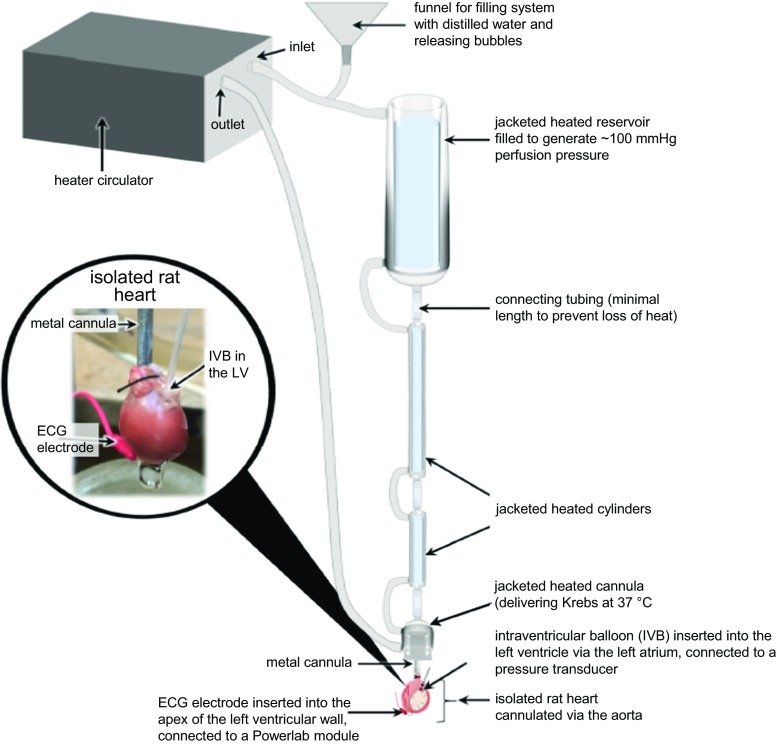
Diagram of a gravitational Langendorff perfused heart rig with an isolated rat heart

Several companies market fully functioning equipment for construction of a Langendorff apparatus. Alternatively, the necessary glassware can be acquired and assembled oneself. One key difference is that kits typically require a peristaltic pump (in either constant pressure or constant flow mode—see below), whereas a self-assembled apparatus is usually simpler and uses gravity to deliver the buffer at the required pressure. The essential components of the apparatus are shown in Fig. 7. The standard technique for infarction studies is retrograde aortic perfusion. In this configuration, the aorta is cannulated and perfused, which causes the aortic valves to shut and the perfusate to flow through the coronary arteries and then through the myocardium (Fig. 7). The apparatus may be designed so as to provide either a constant pressure or constant flow of buffer to the heart. Constant flow requires the use of a peristaltic pump and has the advantage of minimizing the total size of the apparatus and the total volume contained within the tubing. However, it is less suitable when performing regional ischemia, since it will cause an undesirable increase in perfusion pressure. When using constant flow, the perfusion pressure should always be measured and the flow adjusted to maintain a physiological pressure. Constant pressure can be delivered either by placing the buffer reservoir at sufficient height to obtain the desired pressure at the cannula, or using a peristaltic pump with the capability of adjusting to the pressure measured. When using a pump, an inline “Windkessel” device (compliance chamber) is required to minimize oscillations in the flow. This is also useful in either case for eliminating air bubbles.

A cannula may be purchased or made, e.g. from a 21-gauge stainless steel luer-lock needle (for mice), or 3-mm-diameter metal tube (for rats). The end should be filed flat, and machined grooves on the tip can aid in preventing the heart from slipping off. The perfusion pressure, measured at the cannula, should be 70–80 mmHg for rats and mice. In a gravity-fed apparatus this may require adjustment of the height of the buffer reservoir.

It is of vital importance to maintain the cleanliness of the apparatus as even minor contamination can drastically affect results. The apparatus should be flushed through with several liters of boiling water (e.g.: from a kettle) before and after every use. If it has not been used for more than 2–3 weeks, or if cardiac function is unexpectedly poor, the apparatus can be filled with 0.1 mol/L HCl, commercially available detergent or Decon 90, and left to decontaminate for several hours before rinsing thoroughly with distilled, de-ionized water. Note that detergent is advised in place of acid in the case of some commercial apparatus to protect the plastic from crazing. Worn or damaged tubing, particularly in the peristaltic pump, should be replaced.

### Harvesting and cannulation of the heart

Prepare a weight-boat by filling with ice-cold buffer. If necessary, add some crushed ice to keep it cold. The heart must be removed while still beating, and the animal must be deeply anaesthetized or dead when doing so. The animal can be anaesthetized using any standard technique in accordance with regulations, but deep anesthesia (loss of pedal reflex) must be verified before beginning surgery. The use of subcutaneous unfractionated heparin before harvesting the heart is advised. The chest is opened, and the heart is removed with scissors. The aorta should be cut leaving as long a stump on the heart as possible and cannulated as soon as possible to avoid prolonged ischemia. The cannula should be verified as being positioned such that the end is visible 1–2 mm above the aorta’s entry to the heart, and is, therefore, not inserted past the aortic valve.

To cannulate a mouse heart, a dissection microscope or jeweler’s lens is needed. The cannula can be removed from the apparatus. Use a syringe to prefill the cannula with buffer (excluding air bubbles) and place it in a Petri dish containing buffer and a small amount of ice. The cleaned heart can be held with fine forceps by the aortic root, slid over the end of the cannula and secured in place using a suture.

The entire process from cutting the aorta to starting perfusion must be < 3 min. It is absolutely essential to avoid any bubbles entering the aorta either during mounting or subsequent perfusion as this will cause air embolism and infarction. Once mounted and perfused, any remaining non-cardiac tissue can be removed with scissors.

### Perfusate buffer

Krebs–Henseleit buffer (KHB) is considered the most appropriate buffer for Langendorff perfusion and infarct studies. The standard recipe is: NaCl 118.5 mmol/L, NaHCO_3_ 25.0 mmol/L, KCl 4.7 mmol/L, MgSO_4_ 1.2 mmol/L, KH_2_PO_4_,1.2 mmol/L, glucose 11 mmol/L and CaCl_2_ 1.2 mmol/L. High quality, ultrapure water (Milli-Q) should be used. It is recommended to filter the perfusate (< 0.45 µm) as precipitates can otherwise degrade cardiac function [187]. There is some variation in the precise concentration of CaCl_2_ used. A higher concentration was used in the original recipe based on the concentration in blood, but was later adjusted downwards to account for the fact that much of this is bound to albumin [409]. Note that the 11 mmol/L glucose is well above the normal plasma level in rats (5–6 mmol/L) with the aim being to maintain sufficient glucose entry in the absence of insulin [413]. It is also important to be aware that although the heart demonstrates high metabolic flexibility, in situ it typically metabolizes more fatty acids and lactate than glucose [412]. It is possible to add fatty acids to the perfusate but they must be conjugated to albumin, which can cause substantial practical difficulties due to frothing and bubbles, unless care is taken using a non-bubbling aerator to gas solutions. The addition of pyruvate is not generally recommended as it has been shown to be cardioprotective [139, 366]. When using pyruvate 2.0 mmol/L, glucose is reduced to 5.6 mmol/L [388]. A further limitation of KHB is its low oncotic pressure due to the absence of proteins, which is partly responsible for causing edema in the myocardium. It is possible to use blood to perfuse the heart but this requires a donor animal and anticoagulation, and is usually outweighed by the technical complications and disadvantages [35]. Rather, it would usually be preferable to progress to the in vivo model if a more physiological system is required.

The buffers are pre-gassed with 95% O_2_/5% CO_2_ (“Carbogen”) by gentle bubbling through sintered glass, and gassing of the reservoirs is maintained throughout the protocol. The pH of this bicarbonate-based buffer should be 7.4 when bubbled with 5% CO_2_ at 37 °C (without requiring adjustment), and should be verified as such.

### Instrumentation

The heart must be instrumented to monitor perfusion conditions and heart function.

The contractile function of rat hearts is routinely measured so as to verify that the heart is functioning sufficiently well at the beginning of the experiment, as well as to monitor its condition during and after ischemia. Although many cardioprotective agents will result in an improved recovery of contractile function during reperfusion, this may be due to attenuated stunning and/or reduced infarction and, therefore, an additional, more direct measurement of infarction is required to confirm cardioprotection (see below). It is important to note that even under the most optimal conditions of Langendorff perfusion, left ventricular function will deteriorate naturally during the course of the experiment due to edema and gradual tissue damage. A 5–10% loss of contractile and chronotropic function per hour is to be expected [35].

In Langendorff’s original studies, left ventricular contractile function was measured by passing a tie through the apex and connecting it to an isometric force transducer. A more accurate measurement can be obtained by cutting off the left atrial appendage and passing a small, water-filled balloon through the mitral valve into the left ventricle [187]. The left ventricular contractions are isovolumic, but oxygen consumption is determined primarily by developed pressure with little contribution from muscle shortening. Therefore, function more closely resembles left ventricular work in vivo. The balloon pressure can also be increased step-wise to obtain Frank–Starling curves. However, the use of a balloon is particularly challenging with small hearts, and the quality of the data thereby obtained from mouse hearts may not be very reliable. Formation of the balloon is somewhat of an art. It needs to be made of a material that is highly compliant and thin, (e.g.: Saran wrap, cling film, or condom latex is ideal), so as to provide rapid feedback of pressure changes without any dampening. It should be filled with water, mounted on a length of non-compliant tubing (a 21-gauge flat needle can be used for mouse hearts) and tied off with a suture. The volume of the balloon is crucial—its unstressed volume must be greater than that of the ventricle so that the balloon fills the left ventricular cavity without stretching the balloon’s wall which would give an artificially high pressure reading. The balloon is inflated to give an end-diastolic pressure of 5–15 mmHg at baseline. The tubing, filled with water, is connected to a calibrated pressure transducer. This is attached to a recorder. There must be no leaks or air present within the circuit.

The left ventricular balloon can be used to measure a variety of contractile function data, including heart rate, systolic pressure, end-diastolic pressure, and maxima and minima of left ventricular pressure derivative. Left ventricular developed pressure is calculated as the difference between maximal systolic and end-diastolic pressures.

Rate-pressure product is sometimes used as an index of cardiac work and correlates with myocardial oxygen consumption (MV̇O_2_) in larger animals. However, since isolated rodent hearts demonstrate a negative force–frequency relationship (left ventricular developed pressure decrease with increasing heart rate) [409], rate-pressure product is not correlated with oxygen consumption in the Langendorff-perfused isolated rat heart [10].

### Coronary perfusion

Perfusion can be performed either at constant pressure or at constant coronary flow rate. In either case, coronary flow and perfusion pressure are continuously monitored to assess the conditions of the preparation and coronary vascular resistance. Excess perfusion pressure may damage the aortic valve and increase the rate of edema formation during ischemia/reperfusion protocols. Since coronary vascular resistance is equal to perfusion pressure divided by coronary flow, it may seem that there is theoretically no difference between performing experiments at constant perfusion pressure or at constant coronary flow. However, from a physiological point of view some differences exist between the two perfusion modalities. In models of regional ischemia, constant pressure may be preferable; since a constant flow system delivers more coronary buffer per unit of myocardial mass in the territory of non-occluded arteries, which can lead to a damaging shear stress. This issue is less important in the case of a global ischemia model although care must still be taken to avoid an aortic pressure of greater than 80 mmHg which may damage the leaflets of the aortic valve. A constant flow model allows for the presence of an ischemia/reperfusion injury independent of changes in global myocardial perfusion and oxygen consumption due to a Gregg effect (see also below regional and global ischemia) [334].

The coronary flow rate with perfusion of a crystalloid buffer should be around 10 mL/min/g of tissue. In a rat heart, therefore, it should be about 10–20 mL/min for the whole heart. With these flows, the expected perfusion pressure is in the range of 60–80 mmHg. If there is no correspondence between perfusion pressure and coronary flow, problems with the preparation exist and the heart should be discarded if the problem cannot be solved in a couple of minutes. For mouse hearts and for rabbit hearts, the coronary flow is about 2–3 mL/min and 35–40 mL/min, respectively, and pressure is between 60 and 80 mmHg.

#### Constant perfusion pressure model

In the constant perfusion pressure model, the perfusion pressure is usually set between 60 and 70 mmHg (815–950 mm H_2_O), although up to 80 mmHg can also be used (1090 mm H_2_O). The perfusion pressure must be maintained throughout the experiment. This is simply set using gravity by positioning a reservoir and its fluid meniscus at a set distance above the tip of the perfusion cannula (1 mm Hg = 13.6 mm H_2_O). Constant perfusion pressure can also be achieved via a peristaltic roller pump connected with a sealed pressurized chamber. In this modality, pressure can be monitored with a manometer and used to control perfusion pressure in a servo-controlled fashion [409]. Servo-controlled systems can be bought and offer the advantage of using either constant perfusion pressure or constant coronary flow, switching from one to another modality easily.

Retrograde flow in the aorta closes the aortic valve, thus preventing the crystalloid fluid from entering the left ventricle chamber. If the cannula erroneously passes the valve, the heart will not be well perfused and will perform poorly.

In a constant pressure model, coronary flow can be measured by a flowmeter placed within the perfusion line. Also, sequential collection of the heart’s effluent can be used to measure coronary flow, and, if required, the buffer can be recirculated to the reservoir via a micro-filter. However, this procedure can lead to inaccuracy and a flowmeter placed within the perfusion line is preferable; moreover, the non-recirculating procedure is preferable.

A constant pressure model is preferable when auto-regulation of coronary tone is of interest. However, a gravity-driven apparatus requires a large dead space, which may be a problem when expensive agents/drugs are to be used. Moreover, in a constant pressure model, changes in coronary resistance will alter the volume of fluid and the amount of drug delivered to the heart. Therefore, for testing drugs it is better to use a constant flow model.

#### Constant coronary flow model

Constant coronary flow can be achieved with a roller pump connected to a small compliance chamber. This model offers different advantages: (1) constant coronary flow may limit the Gregg effect (myocardial oxygen consumption increases when coronary perfusion increases); (2) it is easier to monitor coronary perfusion pressure with a manometer than to measure changes in coronary flow with a flowmeter; (3) the constant flow model facilitates rapid evaluation of the vasoactive properties of drugs by measuring the changes in perfusion pressure; (4) constant flow simplifies the infusion of drugs in a small volume of fluid, even if the drugs induce changes in coronary resistance.

### Working heart model

There are some important limitations to the Langendorff model of isolated heart perfusion. Most notably, the heart performs less work than in vivo, due to the absence of afterload in the retrograde configuration. Under standard conditions, the saline-perfused Langendorff heart preparation is energy deprived and relies on its stores of glycogen to obtain ~ 1/3 of the glucose needed for oxidative phosphorylation [352], resulting in a lower level of high-energy phosphates (phosphocreatine and ATP) in glucose-perfused Langendorff than in vivo hearts. This situation is slightly improved by the provision of 11 rather than 5 mmol/L glucose and can be further improved by the addition of insulin to drive glucose uptake and metabolism [352]. However, of note, insulin itself is cardioprotective [229].

**Fig. 8:**
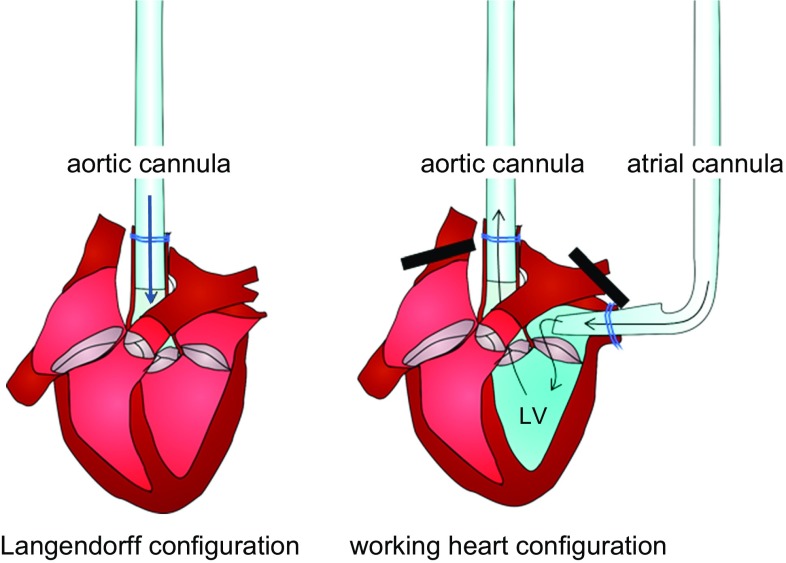
In the Langendorff configuration, buffer from the aortic cannula is prevented by the aortic valve from entering the heart, and passes instead through the coronary arteries (not shown), which originate from the base of the aorta. In the working heart configuration, buffer enters the left atrium via the atrial cannula, passes through the mitral valve to the left ventricle (LV), then is ejected during systole through the aortic valve into the aortic cannula

The hemodynamic limitations of the standard Langendorff model can be overcome by reverting the flow of the buffer through the heart to the antegrade direction, so that the left ventricle is filled via the left atrium and ejection occurs in the normal direction into the aorta (Fig. 8). Consequently, the heart performs physiological mechanical work against a resistance (afterload). In this “working heart” model, as first developed by Neely et al. in 1967 [105, 316, 409], atrial filling pressure is increased and the left ventricle pumps against a hydrostatic pressure head. This model also allows the coronary sinus effluent to be sampled.

Additional equipment is required for the “working mode” configuration but it is commonly used to assess ventricular performance with full control of preload and afterload, oxygen tension and heart rate. It also allows metabolic measurements to be made under more physiological conditions. It has been used for the investigation of myocardial responses to ischemia and reperfusion, particularly in relation to the recovery of ventricular contractile function [240, 307], but it can also be used to evaluate cardioprotection in terms of infarct size reduction [283]. The performance of the working rat heart is stable for up to 3 h [316], but after 1 h cardiac function may decrease [130], and time-matched controls are mandatory.

The following method describes the establishment of a working heart model using rat hearts, but protocols have also been developed for mouse [97, 169, 271], rabbit [23] and pig hearts [76].

The apparatus for the working heart model is similar to the standard Langendorff apparatus but with the addition of a second cannula that is attached to the left atrium (Fig. 8). This line must be capable of delivering at least 150 mL/min for a 1 g heart to ensure that the flow rate supports the maximum cardiac output of a working heart at any particular preload.

Initially, the heart is removed and the stump of the aorta cannulated for retrograde perfusion in precisely the same manner as described for the Langendorff perfusion method. The heart is then retrogradely perfused for at least 10 min to remove all blood and stabilize the heart. Differently from the Langendorff procedure, the pulmonary arteries and veins are then ligated, the left atrium incised and cannulated using a separate venous cannula, the left heart loaded and retrograde perfusion stopped, creating the working heart model.

The Langendorff-perfused heart is positioned to provide access to the posterior of the heart. The right pulmonary artery and veins are occluded using either a surgical clip or suture, followed by occlusion of the left pulmonary artery and pulmonary veins. The pulmonary vessels must be completely occluded to ensure adequate preload. The right ventricle will then increase its pressure, the right atrium will distend, and the heart may become bradycardic. If this does not occur, a further tie or clip may be necessary to eliminate potential leakage. However, as soon as it is established that there are no leaks, the pulmonary artery should be cut to depressurize the coronary veins. Viewing the heart from the anterior side, a transverse incision in the pulmonary artery approximately 3 mm above the pulmonary valve is made with a small scissor, relieving the pressure in the right heart.

Next, the left atrium is cannulated. The upper body of the left atrium is incised a few mm above the atrioventricular groove. Flow of buffer is started so that it drips through the left atrial cannula. The cannula is carefully inserted into the left atrium, such that there is no pressure on the atrial wall. A suture is then tied around the tissue of the left atrium to provide a tight seal around the cannula. The atrial cannula is positioned to ensure that there is free flow, then fully opened to impose preload on the left atrium.

The drips from the heart now come solely from coronary effluent, and its rate should not change, unless there are leaks. Optionally, the coronary sinus effluent can be collected by inserting a piece of 0.8 mm flexible tubing into the incision made previously in the pulmonary artery.

Transition to working heart mode is made by turning off the retrograde aortic pump. Preload (left ventricular end-diastolic volume) is now being delivered from the left atrial cannula. It can be adjusted by changing the pressure of the fluid entering the left atrium. Afterload is delivered via the aortic cannula. A pressure–volume catheter may be inserted into the left ventricle either via the aortic valve or via apical puncture. As with the standard Langendorff configuration, the heart may be electrically paced with wires sutured to the right atrial wall.

Filling pressures for rat hearts are usually in the range of 7–15 mmHg and afterloads in the range 45–75 mmHg. Under these conditions, the coronary flow from the heart from a 250 g rat is up to 25 mL/min and aortic flow is 50–80 mL/min. The end-diastolic pressure is set to about 3–5 mmHg, and the peak systolic pressure will be ~ 100 mmHg.

Because of the large volumes of perfusion fluid pumped by the heart, the working preparation is often operated in the recirculating mode, in which case an in-line 5-µm filter must be included in the circuit to remove any particulate contaminants which may originate from the hearts.

### Temperature monitoring and control

In an isolated heart preparation, it is crucial to continually monitor and regulate the temperature of the heart. This is particularly important in infarction experiments, since infarct size is proportional to the temperature of the heart during the ischemic period [305]. To help maintain the appropriate temperature, a water-jacketed reservoir and heat exchangers along the perfusion line are used. A temperature sensor (micro-thermistor) can be placed on the heart surface or into the right ventricle. Good temperature control can be achieved using a thermocouple. Usually, the selected temperature is the same as the core temperature of the animal species studied (temperature (e.g.: 37.0–37.5 °C. for rat hearts). If temperature tends to decrease as it passes through the tubing to the heart, it is tempting to set a slightly higher temperature for the fluid in the reservoir. However, setting a temperature in the water jacket of the reservoir at more than 0.5–1.0 °C above the required temperature should be avoided to prevent significant overheating in case of an increase in flow. The hearts tolerate a decrease in temperature better than an increase in temperature. A small incision can be made in the right ventricular outflow tract or in the pulmonary artery to retrogradely pass a thin thermocouple into the right ventricle, which is connected to a thermometer. A digital and servo-controlled system can be used to fine-tune the temperature of the fluid in the heated chambers. Although less precise, the temperature can be manually adjusted during the stabilization period by modifying the sources of exogenous heat, e.g.: by immersion in a temperature-controlled organ bath or exposure to a radiant external heat source such as a lamp.

Since the extent of ischemia/reperfusion injury is highly temperature dependent, in such studies temperature monitoring is particularly important and particularly challenging. Usually, with ischemia the heart temperature will drop. Furthermore, reperfusion exposes the heart to rapid variations in temperature. The thin thermocouple placed into the right ventricle during all phases of perfusion provides information about temperature variations during the experiment and can help to avoid these potential variations during the ischemia/reperfusion protocol. Immersion of the heart in buffer warmed to the preferred temperature is the best way to control heart temperature during ischemia/reperfusion. A heart suspended in room air is not a good solution for ischemia studies. Indeed, in this condition heart temperature decreases during ischemia and ischemic injury is affected in an uncontrolled manner.

### Electrocardiogram (ECG)

The ECG provides a convenient and simple measurement for routine assessment of electrical heart function. ST segment elevation provides a simple confirmation of ischemia. One lead of the ECG can be clipped to the (metal) cannula, and the other inserted into the apex of the left ventricle.

### Pacing

The extracorporeal heart is bradycardic due to the absence of neuro-hormonal regulation, and because the sinoatrial node is not supplied by the coronary vasculature, but from extra-cardiac vessels [35, 409]. The isolated perfused mouse heart contracts at 380–420 vs. 580–600 beats/min in vivo, and a rat heart at 250–320 beats/min compared to 350–400 beats/min in vivo. Some investigators prefer to add pacing wires to maintain heart rate and equalize it between hearts, as heart rate can influence cardiac function [409] and influence recovery from ischemia [187]. Pacing may not greatly affect infarct size in hearts from healthy animals, but can be beneficial for diabetic or aged hearts with reduced or variable heart rates. Pacing can be achieved relatively simply using the cannula as one electrode, and a silver wire or stainless steel needle as the other, inserted into the epicardium of the right atrium. Mouse heart pacing can be set at 500–600 beats/min, using a square form wave of 4-ms duration and voltage amplitude of 1.5 × the minimum threshold for stimulating contraction. Rat heart pacing should be set to 300–400 beats/min. Pacing should be paused during ischemia and restarted several minutes after reperfusion has commenced.

### Ischemia/reperfusion

The experimental protocol of ischemia/reperfusion studies consists of three fundamental phases: an initial stabilization period, a relatively long period of test/index ischemia and the phase of reperfusion during which the effect of ischemia and/or the effect of protective stimuli are assessed. Cardioprotective interventions can be performed before, during or after ischemia in the isolated heart model. When using global ischemia, cardioprotective interventions can only be performed prior to ischemia or at the onset of reperfusion.

#### Stabilization

After the cannulation and the beginning of the buffer perfusion, regular heart function is restored within a few seconds. However, more than 10–15 min may elapse before the heart function is stabilized. Therefore, after instrumentation, the experimental protocol should be preceded by at least 20–30 min of stabilisation to be sure that only very little, if any, changes in cardiac function (heart rate, coronary flow, perfusion pressure and left ventricular pressure) are present during this period. 40-min stabilization is recommended if western blot analyses of kinase phosphorylation are included in the study [403].

#### Ischemia

Short periods of ischemia, which may induce only myocardial stunning (a reversible attenuation of myocardial contractile function) under in vivo conditions may already result in myocardial necrosis in Langendorff hearts [96, 335]. Therefore, caution should be used with this model in studying myocardial stunning. In cardioprotection studies, 25–35 min of zero-flow normothermic (37.0–37.5 °C) ischemia is considered optimal for rodents, which results in approximately 50% infarction of the risk area. This infarct size leaves room for an appreciable reduction in its extension following a cardioprotective protocol. In experiments designed to investigate protection using two different drugs or protective modalities, in which > 50% infarct size reduction is anticipated, it may be necessary to use a longer period of ischemia to reveal such additive protection. However, this must be carefully tested, as too long an ischemic period may prevent proper reflow and reperfusion.

The isolated heart preparation can be used for studying global or regional ischemia at either zero or reduced inflow rates. Indeed, the whole heart (global ischemia) or just a part of the myocardium (regional ischemia) can be subjected to flow reduction in the Langendorff preparation. In both cases, ischemia can be achieved at various levels of flow. However, global ischemia allows easier and more precise approaches and more possibilities of different degrees of ischemia (through controlling flow). Regional ischemia in the isolated mouse heart should be avoided because the risk zone becomes so small that the ambiguity in discerning the infarct borders due to a blurry image becomes very significant.

##### Global ischemia

Global zero-flow ischemia is easily induced in the isolated heart model by stopping the perfusion pump, in the case of constant flow model, or occluding the perfusion inflow lines, in the case of constant pressure.

In the constant flow model, restriction of flow can be precisely obtained by adjusting the output of the perfusion pump. In the constant pressure model, a partial and gradual occlusion of the perfusion line can also be applied using, for example, a micromanipulator and a Starling resistance to create a stenosis along the perfusion line. However, there is an advantage to using a pump-controlled system, which with the precise calibration of the pump speed avoids the uncontrolled variation of coronary resistance and coronary flow, which, in turn, may influence the level of damage induced by flow restriction in a biased manner in constant pressure models.

##### Regional ischemia

Regional ischemia can be induced in isolated heart preparations by occluding a coronary artery (usually the LAD), and the size of the ischemic zone (risk area) can be influenced by the different position of the ligation point. The ligation is obtained by placing a snare around the artery. A snare can be made using a small-diameter polyethylene tube size 50 for a rat heart passing through a large-diameter tube. Regional ischemia can also be induced by ligating the artery with a silk thread. To prevent laceration of the vessel wall and to facilitate the reperfusion, the ligature is usually tied to include a small plastic tube placed along the artery.

Since coronary vessels are more easily seen in the presence of blood, the snare can be placed around the artery (without tightening) while the heart is still in situ. However, this procedure introduces additional complications requiring anesthesia, intubation, mechanical ventilation and the surgical opening of the chest.

As a guide for effective tightening of the suture upon induction of regional ischemia, one should expect an instantaneous reduction in coronary flow of at least 30% (of baseline coronary flow) in hearts with a large ischemic zone (placement of the suture proximal to the tip of the atrial appendage), and a reduction in heart rate and left ventricular developed pressure, along with an increase in left ventricular end-diastolic pressure. If these changes are not seen, it would suggest that the suture has not been tightened effectively to induce regional ischemia (zero flow to the ischemic zone), resulting in a smaller infarct. Similarly, if there is no increase in coronary flow or lactate release upon reperfusion, this too is indicative of poor occlusion [443].

In general, it is recommended: (1) to use the heart of rats, a species with minimal collateralization and minimal variation among individuals; and (2) to ligate the LAD proximally, so that if ischemia is accurately induced, the risk zone is about 45–50% of the left ventricle mass and coronary flow and left ventricular developed pressure should decrease by about 30% [35]. This standardized procedure will allow consistent application of the same degree of ischemia in various experiments. Validation of occlusion can also be ascertained by transient infusion of a colored dye or fluorescent particles.

The coronary circulation of some species, such as guinea pigs and hamsters are totally collateralized, thus preventing the study of regional ischemia. In rats and mice (the species most commonly used in ischemia/reperfusion studies), the scarcity of collateral vessels between the various coronary vascular beds is not a significant problem for the study of regional ischemia. Nevertheless, to completely avoid the problem of collateralization, global ischemia can be used. The Langendorff preparation is unique in this sense: it is the only model that allows global ischemia.

The isolated heart preparation is also suitable for studying hypoxia and reoxygenation, again either global or regional. Nevertheless, also in this case, global hypoxia is less complicated for studying the desired level of hypoxygenation by changing the oxygenation of the buffer used. A dual-lumen perfusion catheter can be used to perfuse the left and right coronary arteries separately. This catheter is a powerful tool for studying the regional effects of hypoxia or ischemia [24].

#### Reperfusion

In the global ischemia model, reperfusion can easily be achieved by opening a tap in the constant pressure model, or by restarting the roller pump when using constant flow. In the regional ischemia model, which is usually performed with constant pressure, reperfusion is obtained by loosening/removing the snare—an increase in coronary flow should occur.

Occasionally, ventricular fibrillation may occur when the heart is reperfused. If this does not resolve spontaneously, the heart can be gently flicked or squirted with some cold buffer. If this does not work, a small amount of cold KHB buffer can be injected into the side port of the apparatus. If all else fails KCl 0.1% can be injected into the side port. If the heart still does not recover the experiment must be abandoned.

Either reperfusion or reoxygenation can be performed at various rates and with different modalities (e.g. ischemic postconditioning obtained with intermittent reperfusion) for assessing several features of ischemia/reperfusion damage. Also, reperfusate compositions can be varied, thus providing a powerful tool to study cardioprotective agents of interest.

Duration: to assess infarct size, the duration of reperfusion is dependent upon the animal species used. For the rat heart, 60–120 min of reperfusion is mandatory. Frequent analysis of biomarkers reveals two distinct phases of reperfusion injury [342]. With the mouse heart, there is no detectable difference in infarct size following 30 or 60 min reperfusion [136, 325, 376, 408].

Nevertheless, at least in the mouse heart, infarct size measured by TTC staining depends on both the duration of ischemia and the length of the reperfusion period [353].

## Exclusion criteria

Exclusion criteria (Table 3) must be pre-defined and adhered to without exception, as they indicate problems had occurred with cannulation or perfusion, and inclusion of the data from these hearts may lead to erroneous results.

**Table 3:** Summary of exclusion criteria for Langendorff perfused hearts

Parameter	Mouse heart	Rat heart (250–400 g bw)
Baseline (prior to any intervention, e.g. IPC)		
Time to perfusion	> 3 min	> 3 min
Coronary flow	< 2 or > 5.5 ml/min	< 10 or > 28 ml/min
Arrhythmias	> 10 ectopics during 10 min baseline recordings (VT or VF should not occur)	> 10 ectopics during 10 min baseline recordings (VT or VF should not occur)
Heart rate	< 300 bpm	< 200 or > 400 bpm
Left ventricular end-diastolic pressure	< 5 or > 10 mmHg	< 5 or > 10 mmHg
Left ventricular developed pressure	< 60 or > 140 mmHg	< 70 or > 140 mmHg
Temperature	37 ± 0.5 °C (< 36 °C or > 38 °C for > 1 min)	37 ± 0.5 °C (< 36 °C or > 38 °C for > 1 min)
Reperfusion		
Coronary flow	≤ ischemic flow	≤ ischemic flow
Arrhythmia duration (ventricular tachycardia or fibrillation)	> 2 min (intervene immediately—flicking, cold buffer or KCl)	> 2 min (intervene immediately—flicking, cold buffer or KCl)
Heart rate		< 150 bpm (irrelevant if pacing)
Infarct criteria		
Area at risk	n/a for global ischemia	n/a for global ischemia; for regional ischemia: < 40 or > 70% of ventricular tissue

For mouse and rat hearts, recommended exclusion criteria are: (1) a time between cessation of blood circulation and the start of perfusion in the Langendorff mode of greater than 3 min; (2) a temperature outside the 37 ± 0.5 °C range; or (3) a perfusate flow rate of < 1 or > 6.5 mL/min during a stabilization period of at least 20 min.

For rat hearts, recommended exclusion criteria are displayed in Table 3. The ischemic risk area in regional ischemia experiments should be within a range of 40–70% of the heart mass. If not, the heart should be excluded from analysis.

### Measurement of injury by contractile function

Contractile function (left ventricular developed pressure) decreases during ischemia and recovers slowly during reperfusion. The recovery of function corresponds generally with the degree of tissue infarction [187], but it is important to note that some interventions may reduce infarct size without having a beneficial effect on the recovery of cardiac function [157], particularly when there is a degree of myocardial stunning [256].

### Measurement of infarct size by TTC staining

At the end of reperfusion of rat hearts, the balloon should be deflated and removed from the heart. In a heart subjected to regional ischemia, the suture should be re-tightened around the coronary artery and Evans blue dye injected via the sideport. In all cases, the heart is then removed from the apparatus, weighed and placed at − 20 °C for at least 2 h until frozen through. Make up 0.05–0.075 g TTC per 5 mL phosphate buffered saline and warm to 37 °C in a tube in a water bath. Slice the frozen heart into five equi-width slices from the point of the suture to the apex of the ventricle. Place the sliced tissue into the tube with the TTC solution for 5–15 min in the water bath. Scan immediately, or alternatively drain and place the slices into 10% formalin (5 mL/heart) for 24 h—overnight at room temperature. Arrange the slices between two glass plates, taking care to ensure all the slices are orientated the same way. Images of the slices are then digitally imaged, and planimetry software (i.e. Image *J* and/or Scion Image programs) used to calculate volume of infarct (which appears white), total heart volume, and, if regional ischemia was performed, risk zone volume (blue). The area at risk and infarct size are automatically transformed into volumes. The infarct size is calculated as infarct volume/left ventricular volume, or for regional ischemia: infarct volume/risk zone volume and expressed as percent of the area at risk.

For mouse hearts, it is easier to inject 5 mL of TTC in phosphate buffered saline through the aortic cannula and incubate the whole heart for 10 min at 37 °C. The heart is then weighed and then frozen overnight at − 20 °C. It is then sectioned perpendicular to the long axis, the slices transferred into 10% neutral formalin buffer for 1 h, then infarct measured as above.

For rabbits, the normally perfused area of the myocardium is determined by injection of either Evans blue dye (5 mL, 2.5% in normal saline) or Zn–Cd fluorescent particles (0.8 mg/mL in normal saline), which are infused through the aortic cannula. After 1–2 min hearts are frozen for 24 h at − 4 °C and then sliced in 1–2-mm sections. Sections are then incubated in TTC solution (1% TTC in phosphate buffered saline, pH = 7.4) for 25 min at 37 °C. Subsequently slices are fixed in 4% neutral buffered formalin at 25 °C for at least 1 h. After fixation, slices are pressed between two transparent glass surfaces and scanned/digitized using as above, and area at risk, infarct size and total area of the slice are quantified in each slice and averaged for each heart.

### Measurement of infarct size by necrotic marker proteins in perfusate

Similar to isolated cardiomyocytes, LDH is commonly used. For this analysis, coronary effluent samples are collected at 5, 10, and 15 min from the start of reperfusion, then every 15 min throughout reperfusion, noting the flow rate each time a LDH sample was collected. The samples can be stored on ice. LDH activity in the perfusate can then be determined by means of a commercially available assay kit (e.g.: CytoTox 96 Non-Radioactive Cytotoxicity Assay; Promega, United Kingdom), correcting for coronary flow and heart weight as: LDH concentration (pg/mL) × coronary flow (mL/min/g). Thus, the final units are pg/min/g.

### Harvesting of left ventricular tissue for western blot analysis

Myocardial samples and perfusate can be collected at various time points throughout reperfusion according to the desired parameters to evaluate. For instance, to evaluate phosphorylation of cardioprotective kinases it is recommended to collect myocardial samples between the 7th and 10th min of reperfusion when they are most strongly activated [426]. Moreover, care should be taken to analyze transmural samples [95].

### Hybrid studies using a cross-species approach

Mediators of cardioprotection by local and remote ischemic conditioning are transferable from one individual to another [161] and even between species [211, 225, 304, 381, 385]. A strategy using components, such as plasma or microvesicles from experimental animals, healthy volunteers or patients at different time points before and after a remote ischemic conditioning maneuver and testing their cardioprotective effects in a Langendorff-perfused mouse, rat or rabbit heart used as bioassay, represents a proof-of-concept way to translate the understanding of remote ischemic conditioning’s signal transduction to the clinical setting.

### Transfer of circulating cardioprotective mediators from humans to rabbit hearts

Circulating cardioprotective mediators from healthy volunteers or patients undergoing remote ischemic conditioning (four cycles of 5 min of upper arm ischemia achieved by inflation of a blood pressure cuff to 25 mmHg above systolic blood pressure followed by 5 min reperfusion) can be studied by transferring dialysate of blood samples obtained before and after the intervention to an isolated animal heart in the Langendorff model. Blood samples (150 mL) are collected before remote ischemic conditioning and 5 min after the last cycle of remote ischemic conditioning. Transfer from pigs with remote ischemic conditioning is also successfully performed to isolated mouse and rat hearts, but the preparation of the transferred material is different. For rat hearts, pig plasma is filtered (5-µm pore size, Macherey–Nagel, Düren, Germany) and added with a syringe pump to the perfusate in a 1:6—10 dilutions before passing the heat exchanger. For mouse hearts, pig plasma is placed in 12–14 kDa dialysis tubing (SpectraPor, Spectrum Europe B.V., Breda, NL) and dialyzed at 4 °C against a fivefold volume of modified KHB, resulting in a 1:6 dilution of the dialyzed plasma fraction. The plasma dialysate must be titrated to 2.0 mmol/L CaCl_2_ and 24.9 mmol/L NaHCO_3_, filtered (5-µm pore size, Chromafil Xtra PES-500/25, Macherey–Nagel, Düren, Germany), oxygenated and pre-warmed to 37 °C before use. Mouse hearts are perfused with undiluted plasma dialysate for 15 min before global zero-flow ischemia. In humans, the blood samples are drawn from the contralateral cubital vein, and in pigs arterial blood samples are taken. Samples are saved in heparinized vials, immediately centrifuged at 800*g* for 20 min at 4 °C, and the plasma is either immediately transferred for dialysate preparation or stored at − 80 °C until use. A total of 75 mL plasma is dialyzed for 24 h at 4 °C against 20-fold volume of KHB using a 12–14 kDa Spectra/Por Dialysis Membrane (Spectra/Por, Rancho Dominguez, California). Dialysates are adjusted to 2.5 mmol/L CaCl_2_ and 24.88 mmol/L NaHCO_3_, filtered (5 μm Chromafil Xtra PES-500/25, Macherey–Nagel GmbH & Co. KG, Düren, Germany), oxygenated and equilibrated to 37 °C before use. The resultant yield of 1.5 L dialysate/150 mL human blood sample (app. 75 mL of plasma) is used for interventional perfusion of one isolated heart. Primary endpoint is myocardial infarct size in a global or regional infarct model [304, 381].

Timing of plasma sample collection is important. Plasma taken 5–10 min after the remote ischemic conditioning maneuver by three cycles of 5 min ischemia/5 min reperfusion on one arm is somewhat protective in isolated mouse hearts, but protection is more robust when samples are taken after 30 min, and protection is seen with samples taken up to a week after the conditioning procedure [211].

### Transfer of circulating cardioprotective mediators from humans to mouse hearts

Similarly, circulating cardioprotective mediators can be transferred with human dialysate to an isolated mouse (C57Bl6/J; age 8–12 weeks; weight 20–30 g) heart in the Langendorff mode. When frozen plasma is used, 4 mL is thawed and centrifuged at 4500*g* for 10 min at 4 °C. The supernatant is placed in a 12–14 kDa dialysis tubing (SpectraPor, Spectrum Europe B.V., Breda, the Netherlands) and dialyzed for 24 h at 4 °C against a 20-fold volume of modified KHB. Dialysates are adjusted to 2.5 mmol/L CaCl_2_ and 24.88 mmol/L NaHCO_3_, filtered (5 μm Chromafil Xtra PES-500/25, Macherey–Nagel GmbH & Co. KG, Düren, Germany), oxygenated and equilibrated to 37 °C before use. The resultant yield is used for interventional perfusion of one mouse heart. Primary endpoint is myocardial infarct size, but the activation of intracellular kinases is also assessed by western blot [211].

### Transfer of extracellular vesicles between animals

Extracellular vesicles can be isolated from hearts perfused in Langendorff mode during either aerobic perfusion for 30 min (control) or after exposure to 3 × 5/5 min global ischemia and reperfusion as ischemic preconditioning. Extracellular vesicles are then isolated from the collected coronary perfusates by filtration and differential centrifugation. Perfusates are dialyzed against 0.45% saline containing 5 mmol/L EDTA for 4 h at room temperature to remove calcium ions, then vacuum-distilled to 40 mL. Concentrated perfusates are filtered through 800-nm pore filters and centrifuged at 12,200*g* for 20 min at 4 °C. Pellets are saved as microvesicle fraction. Supernatants are filtered through 200-nm pore filters and centrifuged at 100,000*g* for 90 min at 4 °C. Pellets are saved as exosome-rich pellets, and the supernatant is saved as extracellular vesicle-depleted perfusate. Pellets and perfusates are then reconstituted to their original volume with KHB and can be used in isolated heart perfusion experiments.

To confirm their presence, isolated vesicles must be visualized by transmission electron microscopy. Vesicle pellets are fixed with 4% formaldehyde, postfixed in 1% OsO_4_. Extracellular vesicles are block-stained with 1% uranyl acetate in 50% ethanol, then dehydrated in graded ethanol, and embedded in Taab 812 (Taab Laboratories, Aldermaston, UK). Ultrathin sections are cut and then analyzed with an electron microscope. Hydrodynamic average particle size of extracellular vesicles in perfusates is measured by dynamic light scattering apparatus Zetasizer Nano ZS (Malvern Instruments, Malvern Hills, UK) or by Nanoparticle Tracking Analysis. The presence and amount of extracellular vesicles are assessed by immunoblots from vesicular pellets and extracellular vesicle-depleted perfusates [161]. Extracellular vesicles can then be transferred to isolated heart preparations or recipient animals [396].

## Small animal hearts in situ

### Species (mouse, rat, rabbit)

Small animals (mice, rats and rabbits) are frequently used lab animals for cardioprotection research because they have small size, are relatively inexpensive, and are easy in handling and maintenance.

The mouse model of myocardial infarction was published first in 1995 by Michael et al. [303] to investigate pathophysiological mechanisms of myocardial ischemia. Since then, it has been widely employed in many experimental studies of myocardial infarction, mainly because of the high cost of larger animals and the ability to modify the murine genome which provides a wide variety of knockout or overexpressing lines [435, 438]. This mouse heart permits reliable and reproducible assessment of the area at risk and infarct size [347].

The rat model of myocardial infarction was established prior to the mouse model by Heimburger in 1946 [189] and further modified by other research groups [227, 246, 378]. Throughout the years, the rat model of myocardial infarction has been firmly established since rats are easier to handle and house, have a shorter gestation time, can be genetically manipulated and have lower maintenance costs than larger animals; therefore, they are highly suitable for “high-throughput” studies [64].

The rabbit is frequently used and has distinct advantages in experimental studies of myocardial infarction. Rabbits are medium-sized animals, they have a docile and non-aggressive character, are handled with ease and have a favorable cost-effectiveness [128]. More importantly, the rabbit heart lacks collateral blood flow and has minimal fatal arrhythmia and death after coronary occlusion compared with other species [143, 273].

### Strain, sex, age, weight

#### Mouse

C57BL6 is the standard strain of mice used in the majority of myocardial ischemia/reperfusion injury in vivo models [299]. Among 19 different strains, two strains, namely B6/ecSOD^WT^ and FVB/N mice, have smaller infarct sizes than other strains, indicating that these genotypes are inherently protected and, therefore, not recommended for ischemia/reperfusion studies. C57BL6 mice have average infarct sizes [171], and their use is recommended, since it is the best characterized strain and easily accessible. Since there are different sub-strains of C57BL/6 with different phenotypes and responses to ischemia, it is important to report the precise sub-strain (e.g. C57BL/6J and C57BL/6N) [320].

Age must be considered as a decisive factor of myocardial infarction and cardioprotection [51, 129]. Young mice (4–8 months) are usually used in ischemia/reperfusion experiments. Advanced age (i.e. over 22 months) is associated with increased susceptibility to myocardial ischemia/reperfusion injury and loss of cardioprotection [25, 49].

Female mice are less vulnerable to ischemia/reperfusion. Increased expression of *β*1-adrenergic receptors and estrogens has been proposed to explain pre-existing cardioprotection in female mice [312]. Use of mixed gender in myocardial infarction experiments can complicate the final outcome. For initial experiments, therefore, male mice should be used.

#### Rat

Sprague–Dawley, Lewis and Wistar rats have been used for myocardial infarction studies [276]. Lewis and Wistar are the most commonly used strains in ischemia/reperfusion experiments, present an intermediate infarct size compared to other inbred strains and are adequate for myocardial infarction studies [28].

Gender selection of rats is of utmost significance since estrogen plays a protective role in myocardial ischemia/reperfusion injury. Hearts of ovariectomized rats have more severe myocardial damage and cardiac dysfunction after ischemia/reperfusion than hearts of intact female rats [456]. To avoid the interference of estrogens (depending on the exact phase of the menstrual cycle), male rats should be used in preliminary studies. Rats at the age of 3–4 months weighing 200–300 g are recommended for initial experiments of ischemia/reperfusion.

#### Rabbit

So far, mostly New Zealand white rabbits have been used in myocardial infarction studies [15, 17, 18, 128, 218, 223, 244] although Japanese white rabbits have also been occasionally used [143]. New Zealand white rabbits are less aggressive in nature and have less health problems than other breeds [296].

Estrogen impact on cardioprotective effects and ovariectomized females is more susceptible to cardioprotective maneuvers [101, 367]. For this reason, male rabbits are usually taken for initial studies. Mostly, young adult rabbits are used weighing 3.0–5.0 kg [223, 244].

### Housing and feeding conditions

Housing and feeding conditions must be standardized for reproducibility and conform to the institutional guidelines and national and international laws and policies on the use of animals (Guide for the Care and Use of Laboratory Animals prepared by the National Academy of Sciences and published by the National Institutes of Health; NIH Publication No.85-23, revised 1996).

#### Mouse

Mouse colonies must be maintained in standard conditions [125] (maximum 5/cage—including litters—with a minimum area of 60 cm^2^/mouse, 21–24 °C and 40–80% humidity) and maintained on a 12/12-h light/dark cycle. It is important that mice are not housed together with rats, as they exhibit distress. Standard rodent chow, appropriate for mice, and fresh water must be provided ad libitum, unless special permission has been obtained from the Animal Ethics Committee of the institution to alter this practice.

#### Rat

Rats must be housed in standard conditions [125] (3/cage with a minimum area of 250 cm^2^/rat, 21–24 °C and 55 ± 10% humidity) and maintained on a 12/12-h light/dark cycle with the light on from 7:00 a.m. Rats should be fed the standard rat chow, with water ad libitum [341].

#### Rabbit

Rabbits must be housed in separate cages in an environmentally controlled animal research facility. All rabbits should be exposed to a 12/12-h light/dark cycle; room temperature should be maintained at 17–21 °C and humidity between 30 and 70% [142]. Standard rabbit chow and water should be provided ad libitum. Fasting of animals can be incorporated in special protocols [244].

### Pre-operative preparation

Preoperative preparation of the animals is mandatory to attenuate side-events that might appear during the surgery or postoperatively when a chronic model of ischemia/reperfusion (24 h of reperfusion) is used. Animals undergoing surgery for the induction of myocardial infarction must receive preoperative analgesia and—for chronic experiments—antibiotics. Opioids are cardioprotective per se, and their use before surgery must be considered for their value versus this limitation [116, 397].

#### Mouse

The use of an opioid analgesic, e.g. buprenorphine [0.1 mg/kg subcutaneously (s.c.) twice daily before surgery] is recommended. Non-steroidal anti-inflammatory drugs, such as caprofen (2–5 mg/kg s.c.), can also be administered preoperatively and postoperatively [258]. A broad-spectrum antibiotic should be used to prevent postoperative infections. Cefuroxim [100 mg/kg intraperitoneally(i.p.)] shortly prior to and after the procedure provides adequate antibiotic prophylaxis [118].

#### Rat

Different schemes of analgesics have been employed, such as caprofen, buprenorphine and tramadol [81, 446]. However, the selection of analgesics should be considered with caution. Tramadol reduces infarct size in rats at a dose of 12.5 mg/kg, i.v. [458] and should, therefore, be avoided. Buprenorphine (0.1–2.5 mg/kg s.c.) and caprofen [4 mg/kg i.p. or orally 1 tablet] the day before surgery and one dose of buprenorphine (0.1–2.5 mg/kg s.c.) prior to surgery can be used [446]. Ampicillin 100 mg/kg intramuscularly (i.m.) immediately prior to surgery (2 min before) is suggested.

#### Rabbit

Buprenorphrine (0.08 mg/kg, i.m.) relieves pain both prior to and after surgery [128], while lidocaine (20 mg/kg s.c.) for local anesthesia can also be used [415]. For chronic experiments, broad-spectrum antibiotics such as cefuroxime (25 mg/kg i.m.) should be administered to rabbits before and after the surgery [244].

### Anesthesia

Deep sedation is mandatory for the well-being of the animals and the decrease of mortality from arrhythmias [416]. Opioids can trigger cardioprotection through interaction with their receptors and reduce infarct size [116, 397]. Cardioprotection results also from volatile anesthetics, which can last for several days [34]. On the other hand, the cardioprotective efficacy of remote ischemic preconditioning is lost on a background anesthesia of propofol [260], whereas propofol per se induces cardioprotection [462].

#### Mouse

Anesthesia results in deep sedation, analgesia and immobility throughout ischemia/reperfusion in in vivo mouse studies. The level of anesthesia is evaluated by the loss of a pedal reflex in response to a toe-pinch stimulus as well as by the rate and depth of respiration [43].

A combination of xylazine with ketamine is often used, but xylazine results in low systolic pressure and bradycardia [242]. 100 mg/kg ketamine results in stable left ventricular function and is the recommended dose in mice [447]. Ketamine may cause excessive bronchial and salivary secretions and xylazine can cause bradycardia, undesired effects which may be prevented by atropine at a dose of 0.02–0.05 mg/kg body weight [292].

Isoflurane induces a dose-dependent blood pressure decrease [154], and the recommended dose is 4–5% for induction and 1–2% for maintenance to produce minimal cardiac depression [170]. Caution: isoflurane per se may be cardioprotective [34]. Pentobarbital causes significant bradycardia and contractile depression [451]. The recommended dose is 90 mg/kg as bolus followed by 15–20 mg/kg when required during the experiment [48].

Propofol can be also used for induction of anesthesia and administered intravenously at a dose of 12–26 mg/kg with repeated doses as needed; however, a high dose of propofol (50 or 100 mg/kg) results in significant bradycardia [382].

One of the best anesthetic combinations is ketamine–medetomidine–atropine, which preserves stable blood pressure in different genetic mouse strains. Medetomidine has a high selectivity for alpha-2 receptors [465].

Based on the above, the recommendation for anesthesia in mice is a combination of ketamine, xylazine, and atropine (final doses of ketamine, xylazine, and atropine 100, 20, and 0.6 mg kg^−1^, respectively), or pentobarbital 90 mg/kg as bolus followed by 15–20 mg/kg when required or isoflurane 4–5% for induction and 1–2% for maintenance.

#### Rat

Widely used and generally accepted anesthetics in rats are a ketamine/xylazine mixture or pentobarbital [3, 446]. A mixture of ketamine (50–75 mg/kg) and xylazine (1–5 mg/kg) induces anesthesia [464], while a single injection of pentobarbital [15–80 mg/kg] is also used [216, 446, 464]. However, in a recent study ketamine/xylazine induced bradycardia and increased serum-free fatty acids, while both ketamine/xylazine and isoflurane induced hyperglycemia. Therefore, pentobarbital is currently the best choice at a dose of 50 mg/kg [365]. Volatile anesthetics and propofol should be used with caution since these agents induce long-lasting cardioprotective effects.

#### Rabbit

Short-acting barbiturates are the anesthetics of choice. A single intravenous injection of sodium pentobarbital or sodium thiopental (30 mg/kg) injected into a peripheral vein of the rabbits is sufficient for the induction and maintenance of anesthesia [15, 17].

### Surgery

#### Tracheotomy, intubation and temperature

Orotracheal/endotracheal intubation or tracheotomy is used for ventilation, tracheotomy only after anesthesia.

##### Mouse

Surgical procedures for the induction of myocardial ischemia/reperfusion have been described in detail [55, 303]. In brief, a midline cervical skin incision is performed using micro-scissors after a slight lift of the skin with a straight forceps. The salivary glands are moved out of the way after gentle separation of the tiny muscles overlying the trachea and then fixed aside with ligatures (6–0 prolene). After the trachea is visible, a small cut between the fourth and fifth tracheal cartilage ring is performed and a cannula inserted into the trachea, ligated against the tracheal tube and connected to a rodent ventilator via a plastic tube. In case of experiments with longer reperfusion and recovery of the animals, orotracheal or endotracheal intubations are preferred. In brief, the end (approximately 5 mm) of a Teflon intravenous cannula (20 G) is cropped before inserting it into the mouse trachea. Mouse forelimbs are immobilized using adhesive tape and the upper incisors using a 4–0 silk ligature. The anesthetized mouse is secured against a plastic surface, which is then placed in a supine-angled position. The tongue is held with a small piece of gauze and a light-emitting diode flashlight held against the frontal surface of the neck. The intravenous cannula is inserted into the mouth and passed through the vocal cords. The proper orotracheal intubation is confirmed after connection of the cannula with a syringe bearing one drop of saline (drop will move back and forth with the animal’s in- and exhalation) [329]. If the cannula is falsely inserted into the esophagus, the drop will not move. Artificial respiration (e.g. 120–150 breaths/min, 180 μl volume, 0.8 L/min oxygen flow) is provided throughout surgery by a ventilator [16, 55].

Body temperature plays a major role for infarct size [75, 172, 375]. The temperature must be carefully controlled throughout the experiment by heating pads and lamps with continuous monitoring using a stabilized rectal thermometer. The heating pad should heat to 40–42 °C to maintain normal body temperature around 37 °C throughout the procedure [146].

##### Rat

In brief, a midline cervical skin incision is performed with micro-scissors after a slight lifting of the skin with a straight forceps. Subsequently, the salivary glands are dissociated, and the trachea is prepared by use of a 4–0 silk suture. A small incision is performed between the fourth and fifth tracheal cartilage ring and the endotracheal tube inserted and fixed on the trachea by use of the sutures [446]. In chronic experiments, endotracheal intubation is preferred. Briefly, an endotracheal polyethylene tube size 90- or a 18-gauge scalp vein needle tube, lubed with xylocaine gel, is inserted into the trachea after the animal is secured in a vertical position [214]. For both acute and chronic ischemia/reperfusion experiments, mechanical ventilation is set at 90 breaths/min and a tidal volume of 1.5 mL/100 g room air.

Body temperature can influence infarct size [93]. Body temperature must be maintained in the physiological rage, namely 37 ± 1 °C, by use of a heating pad and continuously monitored by a rectal thermometer.

##### Rabbit

Endotracheal intubation is the technique of choice in experiments that include the recovery of the animals after surgery. Intubation is performed with a 30^°^ rigid endoscope and an uncuffed (or cuffed) endotracheal tube size 2.5–4.5 mm, previously lubricated with xylocaine gel [425]. In acute models of myocardial infarction, tracheotomy can also be used. Briefly, the skin is incised on the middle line with a sterilized scalpel, and underlying muscles covering the trachea are gently pulled aside. Once the trachea is visualized, a small incision (preferably using cautery to avoid bleeding) is performed. Subsequently, an endotracheal tube size 2.5–4.5 mm is inserted into the trachea and fixed by use of 2–0 ligatures [15, 17, 43]. Adequate ventilation of the animals is afforded by 35–40 breaths/min and a tidal volume 15 mL/kg [404].

A thermal mattress is used to maintain body temperature at values of 38/39 °C. Mild hypothermia is cardioprotective and reduces infarct size [172, 424].

#### Thoracotomy, induction of ischemia and reperfusion

An ECG recording from baseline and throughout the ischemia/reperfusion is recommended. Technical details for the ECG recordings are available for small animal experiments with ischemia/reperfusion [377].

##### Mouse

In mice, the anatomy of the coronary arteries is variable and highly debated [263, 362]. The left coronary artery runs to the apex as either one or two major vessels with several smaller branches. It is usually referred to as the LAD because the circumflex artery is considered non-important [303]. The variability of both its branching pattern and vascular territories interferes with reproducibility in the size of the area at risk. Therefore, assessment of the area at risk after the experiment is mandatory [98, 239, 332].

To perform temporary ligation of the LAD, a left-sided thoracotomy in the third intercostal space is the standard and most widely used method. The skin is incised and the subcutaneous tissue dissected, the ventral serrated muscle of the thorax and the intercostal muscles are transected. The opening is broadened with a rib retractor. The lung is gently displaced with a cotton swab to visualize the left auricle, under which the LAD takes course toward the apex. A silk 6–0 suture is mostly used to occlude the LAD just proximal to the site where the artery divides into two smaller branches or alternatively at the level of the tip of the auricle [98]. In the majority of studies, the LAD is ligated between the conus arteriosus and the left auricle, resulting in large areas of myocardial infarction with poor short-term survival [9]. This approach has resulted in great heterogeneity of the final infarct size, ranging between 10 and 70% of the left ventricular region [33, 147, 332, 363, 414].

Ischemia is terminated by releasing the knot, and reperfusion is verified by direct visual inspection.

##### Rats

The animal is placed in its right lateral position and the skin incised using sterilized scissors. Underlying muscles are dissected using a hemostatic forceps. A small incision is made between the fourth and fifth ribs with caution to avoid bleeding and lung injury. The thorax is slightly lifted with the forceps, and a rib retractor is then positioned within the fourth intercostal space, so that the left ventricle is revealed, taking maximal care not to damage the lung. Finally, the pericardium is dissected and the myocardium exposed [138].

In rats, the anatomy of the myocardium is similar to that of mice. However, the circumflex artery is absent. The LAD lies beneath the epicardium and is embedded in myocardial tissue. Therefore, the LAD is rarely visible to the naked eye, creating controversies on the method of its ligation and the outcome of the infarction. LAD ligation very close to its origin induces infarction and severe arrhythmias, resulting in high mortality. On the contrary, LAD ligation at a site close to the apex induces small infarction only [322]. The optimal site for LAD ligation is 2.0–3.0 mm below the anterior–inferior edge of the left atrium along a line connecting the insertion of the left auricular appendage with the apex or 4.0 mm from its origin between the left atrial border and the pulmonary artery sulcus. The ligation is achieved by use of a propylene or silk suture 5.0. The more atraumatic propylene sutures are preferred. LAD ligation at this site induces an infarct size of approximately 65% of the area at risk [214].

Reperfusion is achieved by release or removal of the knot. If animals are required to recover after the surgery, the rib retractor is removed and the intercostal space closed by ligature of the ribs using 2–0 sutures. The negative intrathoracic pressure is restored by a Bülau tube (connected to a three-way stopcock and a syringe). Finally, muscle layers and skin are closed using 4–0 prolene sutures [214, 446].

##### Rabbit

In contrast to smaller rodents (such as mice and rats), the coronary arteries are quite prominent in rabbits and appear as pale red epicardial vessels originating from underneath the left auricle and running down the left ventricular wall. The LAD is small in rabbits and the apex of the heart is supplied by a prominent coronary branch labeled as the circumflex in Ref. [444], and this is the recommended ligation site. This branch can clearly be seen on the LV’s surface anterior to the intraventricular grove where the residual LAD resides, and the branch’s occlusion will render about a third of the ventricle ischemic. The artery branches out along its course to the apex of the heart. These branches are evident mainly as bifurcations in young rabbits and as bi- or trifurcations in aged rabbits [310]. Therefore, ligation should be performed above the division of the vessel at a site approximately 2 cm below its origin.

Open-chest procedures best mimic the clinical scenario [310]. Briefly, hair is removed and the skin incised using a sterilized scalpel and hemostats. Subsequently, underlying muscular layers are dissected. Major vessels, arteries and veins are located superficially under the skin or in the thoracic muscles and can be easily injured during surgical manipulations, causing severe or even ominous bleeding. Therefore, the superficial incisions must be done with caution. Once the ribcage of the animals is revealed, the chest is opened via a left thoracotomy in the fourth intercostal space and maintained open using a chest retractor. After pericardiotomy, the beating heart is exposed. A 3.0–4.0 silk thread is passed around a prominent branch of the coronary artery, and regional ischemia is induced by pulling the ends of the suture through a small polyethylene tube to form a snare. Clamping the tube with a hemostat keeps the coronary artery occluded. The successful induction of ischemia is verified by visual inspection (cyanosis) and by ST segment elevation on the ECG [218].

Reperfusion is achieved by unclamping the tube. Maintaining the suture loosely around the vessel at the site of occlusion facilitates re-occlusion at different time points and for post-mortem staining. Successfully reperfused myocardium is distinguished by the recovery of the color on the ventricular surface; however, bruising of the myocardium can also be observed as a result of hemoglobin degradation [220, 427]. In case the protocol requires recovery of the animals after surgery for further monitoring, the chest retractor is removed, the intercostal space is closed by intermittent ligatures using 2.0 sutures and the muscle layers are sutured together with 2.0 silk sutures [128]. As in rats, the restoration of negative intrathoracic pressure by a Bülau drainage is preferable for the achievement of successful recovery [445].

#### Duration of ischemia and reperfusion

##### Mouse

The duration of ischemia must be sufficient to cause significant infarction, but not complete death of all cardiomyocytes at risk in the control cohort. The most frequent periods of ischemia in mice in vivo are 30 min in duration [299]. Many studies have shown loss of cardioprotective potential after longer periods of ischemia, e.g. 60–90 min [295]. Reperfusion is extremely important for quantitative evaluation of ischemia/reperfusion injury. The time course of reperfusion needed differs for different endpoints. The duration of reperfusion must be sufficient to allow an accurate and unequivocal delineation of necrotic and viable myocardium. Reperfusion is the start of the healing process in reversibly damaged cells, but may also exaggerate injury in irreversibly damaged cells [188]. A minimum duration of 1–2 h reperfusion is mandatory for adequate TTC staining in murine hearts. More prolonged reperfusion, such as 24 h, is recommended when the experimental protocol is designed for investigation of inflammatory responses or when more long-term protection from an intervention is studied.

##### Rat

Ischemia duration in rats is quite standardized, and 30 min of ischemia is the duration of choice. The duration of reperfusion in rats is also quite standardized [393]. Reperfusion for 120 min is sufficient for experiments investigating acute effects of myocardial infarction. Systolic and diastolic left ventricular function deficits can be present as early as 3 h after coronary occlusion [346], while after 3–4 weeks there is hypertrophy which manifests in elevated left-ventricular end-diastolic pressure and right ventricular systolic pressure [339]. Reperfusion for 120 min is the minimum time for infarct size determination in cardioprotection experiments. Keep in mind that shrinkage of the infarcted area with scar formation is observed in long-term experiments (months) [250].

##### Rabbit

30-min regional ischemia is adequate for the induction of myocardial injury and commonly used in myocardial infarction protocols in rabbits [219, 393].

The reperfusion duration can vary among myocardial infarction protocols [46, 393]. Reperfusion for 120 min is inadequate to reveal the full extent of myocardial injury compared to 3, 4 and 6 h reperfusion. Insufficient NADH leakage from irreversibly impaired cardiomyocytes at 120 min reperfusion might lead to underestimation of the actual infarct size (~ 20% of the ischemic area). On the other hand, infarct size does not differ between 3 and 6 h reperfusion (~ 40% of the ischemic area), indicating that 3 h reperfusion is sufficient for an acute model of myocardial infarction [46, 219, 393].

### Endpoints

In studies assessing cardioprotection, the primary endpoint must be the quantitative determination of myocardial necrosis. Determination of infarct size and area at risk is fundamental for ischemia/reperfusion studies [435]. The methods to assess myocardial infarct size in mice, rats and rabbits is TTC staining. Biomarkers have been also used to evaluate the extent of myocardial injury, such as cardiac troponins, CK, and plasma proteins (i.e. macrophage migration inhibitory factor) [90]. The infarct size/area at risk ratio decreases with interventions that protect myocardium against ischemia/reperfusion injury [441].

#### Determination of infarct size

##### Mouse

The area at risk is delineated by injection of microsphere particles or Evans blue staining after re-tightening of the suture at the same anatomical site of the artery where it was occluded. After snare release reperfusion starts, and mice are sacrificed at the end of the reperfusion period; the heart is rapidly excised, directly cannulated and retrogradely washed with 2.5 mL saline–heparin 1% for blood removal. After the tightening of the suture, 2.5% Evan’s Blue solution is infused through the catheter for the identification of the non-ischemic part of the myocardium. Afterwards, hearts are frozen for 24 h and then sliced in 1-mm sections, which are incubated for 20 min in TTC (1% in phosphate buffered saline pH = 7.4) for the identification of the infarct area [16]. Many investigators use the salvage index which is estimated from the formula [(area at risk—infarct size):area at risk] [42, 113, 119]. Other methods for the determination of infarct size in mice include transthoracic myocardial contrast echocardiography [331].

##### Rat

Infarct size is the primary endpoint of most of studies on myocardial infarction in rats. TTC staining is the widely accepted method to measure infarct size [214]. Transthoracic echocardiography [71, 436], CMR [124, 166, 288] and positron emission tomography [175, 177] have also been used in studies of myocardial infarction in rats.

##### Rabbit

Rabbits are euthanized by a high bolus dose of thiopental, and hearts are excised, blood is washed out using heparinized saline, and the suture is re-ligated around the coronary artery. Infarct size is the primary endpoint of most studies on myocardial infarction in rabbits. TTC staining is the most widely accepted method to measure infarct size [46, 285].

For post-reperfusion monitoring of infarct size and ventricular function, other non-invasive techniques such as CMR [127, 128] and transthoracic echocardiography [117, 223, 415] have gained increasing interest.

## Larger mammal hearts in situ

### General considerations

Large animal models, using dogs, pigs, or sheep, have both advantages and disadvantages over small rodent models. Size and anatomy of the heart and systemic hemodynamics, notably heart rate, are closer to those of humans, and potential interventions in large animal models bear closer similarity to potential clinically feasible treatments. Disadvantages of larger animals are the higher costs for purchase and housing. Other than the often inbred and more homogenous genotype of small rodents, large animals have a wider genetic variation which results in larger variation in hemodynamic responses and infarct size development, and which ultimately requires larger sample sizes to detect differences between interventions. Nevertheless, such variability better resembles the clinical scenario and favors translation. The development of transgenic large animal models is costly, time consuming and difficult.

### Animals

#### Species and strains

While dog models were more frequently used in the past [313, 344, 460], pig models are now favored in cardioprotection research [209]. For pigs, several strains such as Göttingen, Yorkshire, Yucatan and CLAWN minipigs, but also full-size landrace pigs have been used. So far—unlike in mice [171] and rats [28]—there is no evidence that specific dog or pig strains are more or less prone to benefit from cardioprotection than others. We recommend dog or pig models as those are established in a large number of experimental facilities, providing not only a comprehensive portfolio of background data (e.g. the expected range of infarct size after ischemia/reperfusion) but also the possibility of collaboration with standardization of protocols across different labs.

#### Age and sex

Compared to patients, animals are most frequently younger. Cardioprotection is more difficult to achieve in very young [369] and very old [51] animals, and use of young and old animals should be avoided unless age is a specific parameter under question. It is yet unclear whether gender influences the effect of cardioprotection in larger mammals; in both male and female animals, cardioprotection from local and remote ischemic conditioning has been validated [129, 312, 393]. The use of sexually mature animals is recommended; thus, dogs aged more than 12 months and pigs aged at least 6–7 months should be used. In particular, in some pig strains such as the landrace, body weight somewhat limits the upper age range, as pigs reach about 75% of their mature weight at an age of 24 months. To ensure a better long-term comparability of data, age and body weight should adhere to a narrow range. Animals should be housed in a local facility for at least 2 and optimally 4 days prior to experiments, as transport may induce substantial stress in both, dogs and pigs, which can potentially modify their response to cardioprotective stimuli within a second window of cardioprotection lasting 72–96 h [58].

### Pre-medication and anesthesia

The anesthetic regimen should be carefully selected to meet the species-specific properties of sufficient anesthesia on the one hand and the requirements for successful translation of the experimental results on the other hand. Local authorities often mandate the exclusive use of veterinary drugs. As these drugs are not used in patients, unknown side effects may interfere with the experimental results. An anesthetic regimen which is also used in clinical cardiovascular surgery is preferred [252, 385]. Of note, anesthetic drugs may have an impact on the study results. Barbiturate anesthesia is associated with cardiodepressive effects [294], and volatile anesthetics are cardioprotective per se [247, 270, 275]. Propofol interferes with the cardioprotection by remote ischemic preconditioning [261]. Such confounding effects must be avoided or taken into consideration.

Sedation is induced by intramuscular injection in the back/neck or hindlimb (pigs: benzodiazepines, e.g. diazepam 0.5 mg/kg or flunitrazepam 0.4 mg/kg [252, 385, 394]; azaperone 4 mg/kg can be added to enforce for quicker effect). After sedation, a vein on the front paw of dogs or an ear vein in pigs is cannulated with an indwelling venous cannula for infusion of short-acting anesthesia (dogs: pentobarbital 30–40 mg/kg; pigs: etomidate 0.3–0.5 mg/kg with/without sufentanil [252, 385, 394]). Anesthesia is maintained by artificial ventilation with oxygen enriched air (~ 35% O_2_ vol.) and volatile anesthetics, e.g. 1.5–2.5% isoflurane, or intravenous infusion of pentobarbital [248]. Due to the unfavorable anatomy of the throat in minipigs, a tracheotomy is an alternative to endotracheal intubation for ventilation.

An alternative anesthetic regimen without potential protection by inhaled gasses in pigs uses intramuscular injection of ketamine (20 mg/kg), xylazine (2 mg/kg), and midazolam (0.5 mg/kg) and is maintained by continuous intravenous infusion of ketamine (2 mg/kg/h), xylazine (0.2 mg/kg/h), and midazolam (0.2 mg/kg/h) [131, 132, 152, 153].

### Experimental preparation and monitoring

**Fig. 9:**
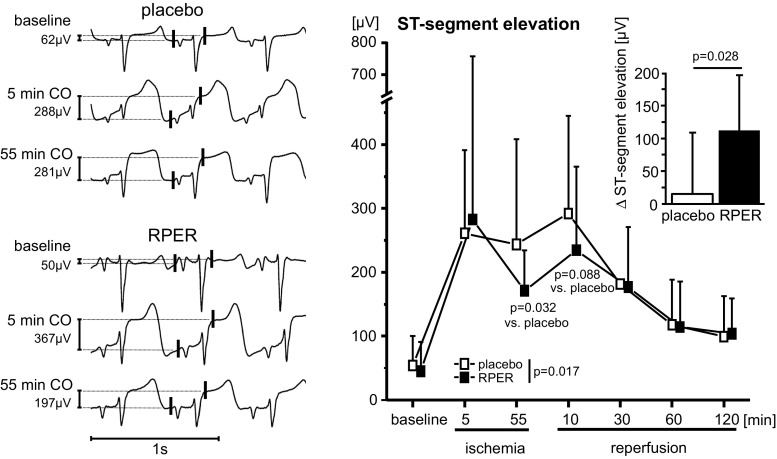
Original ECG recorded in one pig undergoing remote ischemic perconditioning (RPER) and one undergoing placebo intervention (PLA). ST-segment elevation is displayed as amplitude difference between two points (vertical red lines) 30 ms before the *P* wave and 20 ms after the *J*-point, respectively [252]

Large animal preparations should mimic clinical circumstances. Monitoring of rectal or esophageal temperature, end-expiratory CO_2_ levels, arterial blood samples to secure sufficient ventilation and physiologic electrolyte levels are mandatory to warrant a stable preparation. Irregularities, including arrhythmias, should be treated as quickly as possible. If the use of drugs cannot be avoided, their potential interference with the experimental protocol must be considered. Body temperature of dogs and pigs is higher (37.5–39.5 °C) than that of humans [221, 317] and must be kept constant by use of a heated table and drapes. Continuous monitoring of the ECG is not only useful for surveillance of the preparation, but can also serve to reflect the magnitude of injury (ST segment elevation) to assess the success of a protective intervention, and to distinguish between an ischemic and reperfusion-related component of injury (Fig. 9) [252, 354].

For invasive blood pressure measurement and cardiac catheterization, the easiest access is through the internal carotid artery, which may be identified by dissection along the anterior border of the sternocleidomastoid muscle. The internal jugular vein is found alongside the internal carotid artery. Alternatively, the percutaneous Seldinger technique has been used to achieve arterial and venous access but requires use of ultrasound imaging for guidance. This approach is less invasive than the surgical one and recommended if postoperative recovery of the animals is intended. Anticoagulation, particularly when introducing catheter-induced infarction, is advised, but increases risk of diffuse bleeding. Pigs are somewhat resistant to unfractionated heparin and, therefore, 300 IU/kg has been recommended [133].

#### Induction of myocardial ischemia: closed vs. open-chest models

In vivo models use regional myocardial ischemia and thus resemble the clinical scenario of STEMI. Regional ischemia is induced by temporal occlusion of a large epicardial coronary artery, commonly the LAD distal to its 1st or 2nd diagonal branch. Coronary occlusion can be achieved either by direct ligation using a tourniquet (leave in place for later demarcation of area at risk, see below) [385] or by an intracoronary balloon, placed under fluoroscopic guidance [131, 132, 152, 153, 248]. Permanent occlusion of the left circumflex coronary artery can also be achieved by placement of an intracoronary coil, which electrically generates a thrombus with a subsequently large infarcted area, as well as mitral regurgitation and atrial infarction [8]. Ligation by a tourniquet requires a thoracotomy which changes the intrathoracic pressures and thus affects hemodynamics, but these effects are minor and not prohibitive for precise measurement of left ventricular function. Exposure of the heart by either sternotomy or left lateral access depends on the body frame of the animal. Minipigs do not tolerate a prolonged position on their back due to their compact body frame, and lateral access is preferred. An obvious advantage of the open-chest model is that the area at risk is anatomically precisely defined distal to the placement of the ligature and an unintended closure of side branches is virtually impossible. The placement of an intracoronary balloon under fluoroscopic guidance makes the determination of area at risk less accurate. Intracoronary delivery of microspheres (diameter of 40–50 µm) through a catheter or with direct injection into the coronary artery is used to induce microembolization [115, 389] to study the effects of coronary microembolization per se [387] and its interference with cardioprotective maneuvers [386, 390, 394]. On the other hand, the closed chest model with coronary occlusion performed by a balloon catheter is clinically more relevant as it mimics the clinical procedure for PCI [29, 132].

#### Hemodynamics, myocardial blood flow and regional myocardial function

Placement of open-end catheters from the internal carotid into the left ventricle and through the femoral artery into the aorta provides a simple access to measure left ventricular and aortic pressures, respectively. A more precise measurement of ventricular pressure can be achieved by a high-fidelity pressure-tip catheter placed in the left ventricle or by a micromanometer implanted into the left ventricular apex. Such high-resolution pressure measurement avoids catheter-induced over- and undershoots in the pressure signal [241] which is important in studies investigating complex changes in systolic and diastolic ventricular function, e.g. the maximal rise of left ventricular pressure during systole and calculation of the left ventricular relaxation time constant.

Continuous digital data acquisition systems (e.g. Notochord, Notocord Systems, Croissy-sur-Seine, France; Biopac, Biopac systems, Goleta, CA, USA; Labchart, ADInstruments Pty Ltd, New South Wales, Australia) allow off-line analysis of hemodynamics, ECG, respiratory parameters, and temperature.

The measurement of regional myocardial blood flow is mandatory in species with high collateral blood flow, notably in dogs [279], but it is also recommended in species with low collateral blood flow, e.g. pigs, where there still is an inverse relationship between residual blood flow and infarct size [384]. Even the rather small variation in residual blood flow during ischemia in pigs is reflected by the more frequent occurrence of ventricular fibrillation and by larger infarcts [384]. The gold standard for measurement of regional myocardial blood flow is the injection of labeled microspheres [112] into the left atrium with simultaneous, calibrated withdrawal of blood from the descending thoracic aorta [252, 384, 385, 395]. Use of color-labeled microspheres (FluoSpheres, Life Technologies, Eugene, OR, USA) is easy, but somewhat labor intensive in the subsequent sample processing [262]. Neutron-activated microspheres (BioPal, Worcester, MA, USA) can only be analyzed at the manufacturer’s laboratory which induces additional costs and delays. Radioactive-labeled microspheres are now obsolete.

Regional myocardial contractile function can be measured by transit-time [418] or Doppler [178] sonomicrometry in the area at risk and a remote control area as reference, and regional contractile function is usually expressed as systolic wall thickening or systolic segment shortening. Calculation of these variables requires exact timing of diastolic and systolic time intervals which can be derived from high-fidelity left ventricular pressure signals or the ECG [392].

#### Blood samples, microdialysis, tissue samples/biopsies

Blood and/or tissue samples can be taken at various time points of the protocol. In this respect, the larger mammal heart size is favorable. Accurate timing in relation to ischemia/reperfusion and interventions is of utmost importance as biologic effects may occur within very limited time frames, e.g. in the first few minutes after myocardial reperfusion [373, 374, 390].

Microdialysis catheters can safely be placed in the ventricular wall to sample fractions of interstitial fluid during an experimental protocol and to evaluate changes in, e.g. adenosine, lactate and other metabolites [297, 374].

Large mammal hearts permit multiple sampling of myocardial tissue from the area at risk and remote areas which can serve as an intraindividual Ref. [205, 393]. Using hollow-core drills with a diameter of 2 mm, transmural tissue samples of about 10 mg can be retrieved. Of note, taking biopsies from areas with apparent arteries and veins must be avoided and sutures must stop bleedings from bore holes. Experiments with myocardial tissue samples bear the risk of arrhythmias. If large volume blood samples are withdrawn, e.g. for preparation of plasma/plasma dialysate [385], the volume loss must be compensated with saline.

#### Imaging

Cardiac imaging can be performed in dogs and pigs with protocols similar to human investigations. CMR [60, 315], computed tomography, echocardiography [102, 103], positron emission tomography [318, 372], single-photon emission computed tomography and fluoroscopy have been performed successfully in dogs and pigs. Due to the chest shape, it may be difficult to obtain optimal scanning angles, which may compromise advanced echocardiographic measurements, e.g. with tissue Doppler techniques. For the precise measurement of ventricular function, we recommend invasive methods or CMR.

#### Ischemic conditioning

Local ischemic pre- or postconditioning can be induced using an intracoronary balloon or a tourniquet in open-chest preparations. Remote ischemic conditioning is mostly achieved through intermittent restriction of blood flow in a limb. Dissection of the femoral/iliac artery and intermittently occluding it with a tourniquet can be used to obtain intermittent limb ischemia/reperfusion, but external compression using a strong tourniquet around the proximal part of a limb is easier and equally efficient [248, 252, 385]. Verification of limb ischemia and successful reperfusion must be performed. The timing of ischemic conditioning protocols in relation to the index ischemia in the heart is important and should be kept strict. The number and duration of the conditioning cycles must be kept constant and accurately reported. In pigs, three or four cycles of 3–5 min local or remote ischemia/reperfusion induce efficient and reproducible cardioprotection in most [158, 228, 252, 385, 393], but not all studies [29]. In a recent study in pigs, remote ischemic perconditioning attenuated ischemic injury even before reperfusion, as assessed by attenuated ST-segment elevation during ongoing ischemia [252]. The evolution of intracoronary ST-segment elevation during ischemia can also be analyzed, even in the clinical settings to assess cardioprotection during PCI [429].

#### Pharmacologic pre-, per- or postconditioning

Infarct size reduction after ischemia/reperfusion has been performed with a number of drugs (e.g. adenosine [452], glucagon-like peptide 1 analogs [121], metoprolol [217], and CsA [391]) in dogs and pigs. However, translation to clinical use has so far not been successful, and a consistent improvement of outcome in patients with STEMI has not yet been reported. Such discrepancy may be explained by inter-species pharmacokinetic and pharmacodynamic differences, but may also be related to the more complex nature of clinical ischemia/reperfusion scenarios, often confounded by comorbidities and comedications compared to the rather simplified experimental models. Nevertheless, pharmacological studies can provide important mechanistic insights [201, 254]. As with mechanical conditioning strategies, timing is of major importance. Generic drug names, producer, solvents, dosage, timing, and administration route must be clearly reported in detail. Measurement of serum levels of the administered compound is recommended to secure that a sufficient concentration is reached. It may be particularly important for future experiments to perform studies on novel pharmacological targets with a pre-existing background of P2Y_12_ antagonists which are now routine clinical anti-platelet therapy, share cardioprotective signaling with ischemic conditioning and induce cardioprotection per se [85, 448–450].

### Area at risk and areas of no reflow

Prior to euthanasia, it is important to delineate the perfusion territory exposed to prolonged ischemia and reperfusion, i.e. the area at risk. Direct access to the coronary artery is necessary, and if a closed-chest model has been used, a thoracotomy should be performed at this stage and a tourniquet placed around the coronary artery at the best estimate of the site of balloon occlusion; perivascular hematoma may guide the placement. Commonly, dyes (e.g. Evan’s blue, Patent blue, fluorescein) or fluorescent microspheres are used to demarcate the area at risk. After re-occlusion of the coronary artery, the dye or microspheres are injected into the left atrium, and after staining of remote areas the heart is arrested by quick injection of 2 mol/L potassium chloride solution or electrical induction of ventricular fibrillation.

Areas of no reflow can be demarcated at the end of an experiment by thioflavin S stain which binds to endothelial cells and thus tissue with intact perfusion at the time of injection. One mL/kg body weight of pre-warmed 4% thioflavin-S solution (Morphisto, Frankfurt, Germany) is filtered through a 0.2-µm pore syringe filter to remove particulate debris before slowly infusing it over 2–3 min into the left atrium prior to re-occlusion of the coronary artery. In *post*-*mortem* inspection of tissue slices, thioflavin S-stained tissue exhibits a yellow-green fluorescence when examined under ultraviolet light (340–360 nm). Areas of no-reflow remain without fluorescence (thioflavin-S negative) [173, 384]. Fluorescence can be documented by digital photography in an ultraviolet-illuminated light box.

### Euthanasia

General advice cannot be given as euthanasia of laboratory animals has to comply with national regulations.

### Infarct size

After the heart is excised and flushed with cold saline, it is cut into slices parallel to atrioventricular grove and measured using TTC. Note that the area at risk must be documented before TTC-staining, as the deep red formazan can mask the blue stain used to detect the area at risk. A blinded observer must quantify infarct size. CMR-based infarct size quantification is a valid alternative to pathology, in particular in studies with longer follow-up in which ventricular function is also addressed and scar formation quantified.

### Exclusion criteria

Exclusion criteria must be defined prospectively. Exclusion is justified by severe anesthetic incidents and surgical faults during the initial preparation, by persistent arrhythmias such as atrial fibrillation and intractable ventricular fibrillation, by higher residual blood flow during ischemia (> 0.06 mL/min/g in pig myocardium) [252, 384], by large areas of non-reperfused tissue as determined from measurement of regional myocardial blood flow at early reperfusion (e.g. 10 min), by hypo- or hyperthermia with more than 1 °C difference to normal body temperature, and by obvious errors in the timing of the experiment.

## The human right atrial trabeculae model of simulated ischemia and reperfusion

The human atrial trabeculae model of simulated ischemia/reperfusion injury was developed by the Yellon group in 1995 [440]. It uses human atrial trabeculae isolated from right atrial appendages harvested at the time of cardiac surgery [440]. The human atrial model has proved to be a unique translational tool, confirming in humans what has been shown in cell models and in vitro/in vivo animal studies [253].

### Outline of model

Human atrial muscle is taken from patients undergoing cardiac surgery following informed consent.

The exclusion criteria typically consist of

1. Patients over the age of 80.
2. Patients who have had a troponin-positive event within the last 6 weeks of being admitted for surgery.
3. Patients with unstable angina in the last 72 h.
4. Patients in permanent atrial fibrillation or any arrhythmic episodes such as atrial flutter or ventricular tachycardia within the last 6 weeks, or on any anti-arrhythmic therapy such as amiodarone.
5. Patients with congestive heart failure (pulmonary edema and/or ejection fraction < 50%).
6. Diabetic patients are excluded from all experiments except the experiments specifically required for the study under investigation.
7. Patients with renal failure.

### Sample collection and transport

During surgery, and prior to insertion of the venous cannula of the cardiopulmonary bypass machine, right atrial appendages are harvested by placing a purse string suture around the base of the appendage. Appendages are immediately placed in a falcon tube containing 50 mL modified Tyrode’s buffer (comprising in mmol/L 118.5 NaCl, 4.8 KCl, 24.8 NaHCO_3_, 1.2 KH_2_PO_4_, 1.44 MgSO_4_·7H_2_O, 1.8 CaCl_2_·2 H_2_O, 10.0 glucose, and 10.0 pyruvate) oxygenated with a 95% O_2_–5% CO_2_ gas mixture to maintain pH between 7.35 and 7.45, pO_2_ > 55, and pCO_2_ between 4.0 and 6.0 kPa. In addition, the temperature is maintained at less than 4 °C by transporting the falcon tube in a thermos flask containing ice as quickly as possible to the laboratory.

Alternatively, appendages are transported and trabeculae are dissected in cardioplegic buffer (in mmol/L: 100 NaCl, 10 KCl, 1.2 KH_2_PO_4_, 5 MgSO_4_·7H_2_O, 50 taurine, 5 3-(*N*-morpholino)propanesulfonic acid) at 4 °C. The average transport time must be kept to a minimum, i.e. from operating theater to laboratory.

### Dissection of atrial trabeculae

**Fig. 10:**
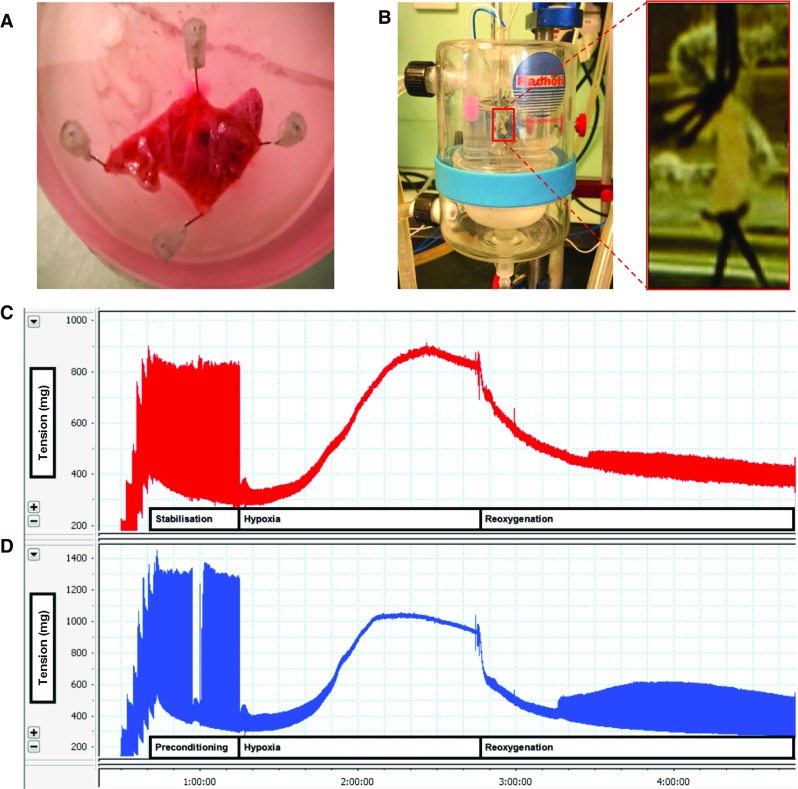
**a** Atrial trabeculae on the surface of the appendage being dissected. **b** Atrial trabecula mounted between two platinum pacing electrodes. **c** Functional readout from the atrial trabecula showing the change in contractile function (difference in tension) during hypoxia and reoxygenation. **d** The recovery of contractile function at the end of the experiment is greater in an atrial trabecula that had been preconditioned prior to hypoxia and reoxygenation

Once in the laboratory, the sample is placed in buffer contained within a clean sterile Petri dish on a bed of ice. The inner surface of the sample is placed upwards and its edges pinned down, to expose the atrial trabeculae on the inside (Fig. 10a). Trabeculae are then isolated by tying a surgical knot at either end of the trabeculae with 5–0 silk. The trabeculae are excised and suspended in a 25-mL water jacketed organ bath between two pacing electrodes (Radnoti Glass Technologies, Dublin, IE) containing modified Tyrode’s buffer bubbled with a 95% O_2_–5% CO_2_ gas mixture, to maintain pH between 7.35 and 7.45, pO_2_ > 55, and pCO_2_ between 4.0 and 6.0 kPa (Fig. 10b). The temperature is maintained at 37 °C using a heat exchanger.

Alternatively, measurements of force of contraction are performed in a modified four-chamber myograph (Multi Wire Myograph System 610M, DMT, Denmark). Atrial trabeculae are mounted between a fixed and a mobile steel hook which is attached to a force transducer. Pacing electrodes are inserted in polyvinyl chloride cubs, covering the bottom and sides of the chambers. Inserted tubes enable gas supply and buffer changes. After pacing atrial trabeculae with rectangular pulses (5 ms) and a field stimulation at 1 Hz, trabeculae are gradually pre-stretched to their maximal force of contraction [267, 311]. When further stretching does not cause greater force of contraction [311], stretch is reduced to 90% of the length with such maximal force [99].

### Simulation of ischemia and reperfusion

The trabeculae are bathed continuously in the modified Tyrode’s buffer coming from a chamber which is oxygenated with 95% O_2_–5% CO_2_ gas mixture and maintained at 37 °C with the buffer being re-circulated. Pacing electrodes in the organ bath are attached to stimulators and the trabeculae are paced constantly at 1 Hz. One end of the trabecula is attached to a quad bridge transducer which creates an electrical voltage that is proportional to the force generated by the muscle strip. A Powerlab (AD Instruments) then digitizes that voltage and stores the digital data on a computer for future analysis with Chart five for Windows software. The amplitude of the spike represents the force of contraction of the trabeculae. The trabeculae are then allowed to stabilize.

At the end of the stabilization period, ischemia is simulated by replacing the oxygenated glucose-containing Tyrode’s buffer with a hypoxic, glucose-free buffer (containing in mmol/L 118.5NaCl, 4.8 KCl, 24.8 NaHCO_3_, 1.2 KH_2_PO_4_, 1.44 MgSO_4_·7H_2_O, 1.8 CaCl_2_·2H_2_O, 7.0 choline chloride) from another chamber and stimulation is increased from 1 Hz to 3 Hz to deplete ATP levels. The induction of ischemia is immediately apparent on the developed tension recording chart, as indicated by a reduction in contractile force (Fig. 10c). Simulated ischemia is maintained for 60–90 min before the trabeculae are reoxygenated (reperfused) with oxygenated modified Tyrode’s buffer and the pacing returned to 1 Hz during the reoxygenation period (Fig. 10d). This is continued for 30–120 min until the end of the experiment. At the end of the experiment, the length and width of the trabeculae are measured along with the weight in milligrams.

### Recovery of function

The amplitude of the contraction spikes, representing the force of contraction, is recorded on a spreadsheet at baseline and at various points during the simulated ischemia and reperfusion period. The cross-sectional area of trabeculae is calculated by dividing muscle mass by length times density, assuming a cylindrical shape and a density of 1.0 mg/mm^3^. To ensure that comparisons of developed force of contraction are not affected by variable muscle size, the cross-sectional area of the right atrial trabeculae is used to calculate developed contractile stress in mN/mm^2^ [309]. Additionally, the developed contractile stress can be expressed as a percent of baseline.

**Exclusion criteria:**

1. Trabecular diameter greater than 1.2 mm
2. Damaged trabeculae, determined visually
3. Irregularly contracting trabeculae either at baseline or at the end of reperfusion
4. Baseline force development less than 4 mN

### Critical analysis of the model

The human atrial trabecular model is a functional model that uses force of contraction as a measure and recovery of function as a surrogate marker of myocardial injury. A drawback with this model is that the human myocardium utilized is atrial and not ventricular because of the ease of availability of atrial tissue as opposed to ventricular tissue.

The human atrial model depends on superfusion as a method of delivery of oxygen and solutes to its cells. The diffusion of these substances into the inner core of the trabeculae is inversely proportional to the diameter, the cut-off value being 1.2 mm. Trabeculae larger than 1.2 mm in diameter do not behave the same way when subjected to simulated ischemia/reperfusion.

## Unbiased “omics” technologies for mechanistic studies and identification of molecular targets

Problems of reproducibility of mechanistic experimental data in cardioprotection studies have become evident by now [255, 371]. This may be at least in part due to strong hypothesis-driven biased selection of molecular mechanisms that may lead to lack of proper randomization and biased interpretation of the data on top of general biological variations [337, 371]. Therefore, studies aiming at acute cardioprotective mechanisms and/or identification of potentially cardioprotective molecular targets may be performed using unbiased “fishing” approaches, such as robust “omics” assays, i.e. genomics, epigenomics, transcriptomics, epitranscriptomics, proteomics and metabolomics followed by bioinformatics and systems-based analysis to predict the most relevant pathways or targets [337, 432].

### Genomics and epigenomics

Since the completion of the Human Genome Project [268, 434], a major effort has been undertaken to link this information to a healthy or unhealthy cardiac phenotype. However, it has become evident by now that several additional mechanisms related to epigenomic, transcriptomic, epitranscriptomic, proteomic, and metabolomic regulations might be crucial to determine the pathological phenotype of myocardial ischemia/reperfusion injury and cardioprotection. As the genetic sequence is largely static from birth, genomics have little chance to reveal molecular targets for acute cardioprotection. However, the relatively fast epigenomic alterations may be relevant for cardioprotection. So far, there is a paucity of studies in this field due to technical limitations to measure all epigenetic changes [337]. As an example, in mice subjected to ischemic preconditioning, histone H3 lysine 9 demethylation (H3K9me2) levels were increased in the area at risk compared to remote myocardium, and this epigenetic mark was proposed to be involved in the cardioprotective effect of ischemic preconditioning through regulation of autophagy [160].

### Transcriptomics and epitranscriptomics

While transcriptomics refers to the characterization of gene expression at the ribonucleic acid (RNA) level, epitranscriptomics refers to post-transcriptional RNA modifications; the most frequent ones are pseudouridine (also called the “fifth nucleoside”) and *N*^6^-methyladenosine [192]. Currently, transcriptomics seems to be useful for unbiased mechanistic studies at the transcript level, including coding or non-coding RNAs, since current technologies allow full and accurate assessment of all transcripts in biological samples [333, 337, 432, 433]. Transcriptomics can be performed by different techniques [337]. Since these techniques require special and rapidly changing technologies (e.g. deep sequencing, DNA microarray, nanostring, etc.), such measurements are suggested to be outsourced to specialized core facilities or R&D companies. However, proper sample collection and handling, RNA isolation, quality check of the RNA isolate, transport, and omics data evaluation are key issues to obtain reproducible data [337].

Not much is known on the potential role and regulation of epitranscriptomics in cardioprotection. Several technical constraints have to be overcome before more insight can be obtained.

#### Sampling and storage

Different types of samples can be used for transcriptomics analysis, from liquid biopsies to cardiac tissue samples. It is important to follow simple recommendations and to limit the variations between the collection procedures to ensure sampling quality and consistency of results. Liquid biopsies have to be collected in tubes devoid of any heparin, since this anti-coagulant is able to inhibit, even in low concentration, downstream applications such as the most widely used polymerase chain reaction for gene expression analysis [53]. Platelet-free plasma samples have to be stored at − 80 °C in aliquots and assayed as soon as possible, but no later than after a very few years. Before aliquoting, it is essential to homogenize the centrifugation supernatant of the blood samples to ensure homogeneity between aliquots. Plasma samples can be stored at − 20 °C for maximally 1 month to avoid RNA degradation. Storage has to be done in RNase-free tubes, and repeated freeze–thaw cycles must be avoided since, unlike miRNAs, not all RNAs are resistant to temperature variations.

Whole blood samples are also used for transcriptomic biomarker discovery [104]. Whole blood samples must be collected in special tubes containing a RNA-stabilizing solution, thereby allowing recruitment of patients and collection of blood at any time of the day without the need for immediate processing. These tubes, providing the blood is correctly homogenized with the stabilizing agent by at least ten inversions, can remain at room temperature for up to 3 days before long-term storage at − 20 or − 80 °C.

Heart tissue samples have to be collected in RNase-free tubes filled with RNA stabilization solution and stored at − 80 °C and assayed for transcriptome as soon as possible but no later than after a very few years.

Unfortunately, there are no valid data on the long-term stability of different transcripts, some being more stable than others. Therefore, time-matched sampling and storage time as far as possible are recommended. This is even more relevant to epitranscriptomics studies since the stability of many RNA modifications has not been determined.

#### RNA isolation and quality check

After collection of biological samples, a variety of commercially available RNA isolation kits enable RNA isolation. Due to the different kind of kits, it is not recommended to use different kits in the same study, since some will favor the isolation of short RNAs while others will enrich the RNA fraction with long molecules. RNA isolates must be stored at − 80 °C in RNase-free tubes, in several aliquots when the quantity of RNA is sufficient. Before sending the RNA isolates for transcriptomic assays, the quality of RNA isolates should be checked to make sure that the total RNA concentration and the RNA integrity number are suitable for the actual transcriptomics technologies used [27]. Some techniques to assess RNA expression are less sensitive than others to RNA degradation. Undegraded RNA, however, is mandatory to obtain reliable results.

#### Data evaluation

Raw data should be obtained from the transcriptomics assay location, and data evaluation should be done by a bioinformatics professional, or by the application of user-friendly, validated, commercial bioinformatic tools for transcriptomic data analysis and target pathway predictions based on, e.g. microRNA expression fingerprint or full messenger RNA expression profiling [7].

#### Validation of transcriptomic data

Transcriptomic data and bioinformatic predictions based on such data must be always validated experimentally by targeted real-time polymerase chain reaction analyses at the RNA level and/or at the protein level by antibody-based assays or targeted proteomics [337]. Replication of findings in another set of animal experiments or in independent patient cohorts is paramount to obtain reliable and reproducible findings.

### Proteomics

Proteomics address a large-scale characterization of protein species in biological samples. However, unlike in transcriptomics, a protein equivalent of the polymerase chain reaction does not exist, such that amplification of minute samples is not possible. This results in the major limitation of unbiased proteomics analysis, i.e. the large abundance proteins mask the changes of low-abundance proteins which also make a significant contribution to cellular signalling. Discovery proteomics using high-resolution mass spectrometry gives the most unbiased view of proteins; however, quantification especially of low-abundance proteins is very limited. Targeted discovery or targeted proteomics focusing on selected panels of proteins gives more accurate quantification of low-abundance proteins, but loses the unbiased feature of proteomics. Moreover, when analyzing cardiac tissue samples, blood contamination with plasma proteins and other intracellular high-abundance proteins significantly masks any possible changes in low-abundance proteins and post-translational modifications [165]. Currently available techniques to remove top or medium abundance proteins are not sophisticated enough to exclude depletion of low-abundance proteins of interest. Moreover, protein function is largely affected by more than 300 post-translational modifications at over 500,000 post-translational modification sites. However, proteins with post-translational modifications are typically low-abundance proteins—again no unbiased technique is available for their detection. Therefore, targeted proteomics with special techniques and further chemical modifications or labeling are required [277]. In conclusion, so far proteomics show several limitations that restrict the use of this technology for unbiased discovery for all proteins, especially with their posttranslational modifications, but the technologies are rapidly developing [280]. Similarly to transcriptomics, discovery proteomic results should be experimentally validated by targeted proteomics and/or antibody-based assays.

### Metabolomics

**Fig. 11:**
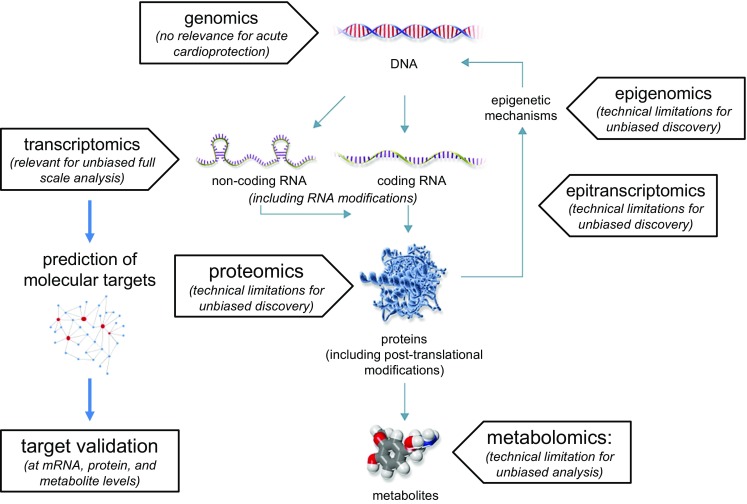
Different omics approaches from the genome to metabolome. Current technologies so far allow unbiased analysis of all genes and transcripts. Based on transcriptomic fingerprints, in silico prediction of molecular targets can be achieved. Predicted targets must always be validated at the protein level. Other omics technologies are rapidly emerging; however, their use is currently limited to assess only fractions of the total epigenetic/transcriptomic, proteomic and metabolomic pool

Metabolomic technologies based on mass spectrometry or NMR techniques after pre-analytical chromatography sample preparation to reduce metabolite diversity can measure thousands of small molecule metabolites from biological samples. Given the total number of small molecule metabolites in humans (estimated up to 100.000 in Human Metabolome Database) and the fact that only a fraction of them can be measured by high-throughput metabolomic technologies (typically measuring around 1000 molecules even in full scan discovery mode at a time) so far metabolomics are not useful for unbiased analysis of all metabolites [73]. Nevertheless, targeted metabolomics may serve as a reflection of protein functional activity and be useful for validation of proteomic data at the metabolite level (Fig. 11).

### Multiomics

Taken together, the transcriptomics approach fulfills the term of unbiased target discovery as this technology allows measurement of theoretically all transcripts in biological samples. Targeted proteomics and metabolomics are very useful tools for validation of molecular targets identified from transcriptomics data. Nevertheless, pulling together transcriptomic, proteomic and metabolomic data by multiomics approach, based on the concepts of systems biology, will open up a new field to identify biomarkers and molecular targets for complex diseases, such as ischemic heart disease, and eventually cardioprotection [26, 337]. Some preliminary data using multiomics recently gained more insight into mechanisms of cardiac remodeling in mice. The authors used integration of transcript abundance, protein abundance, and protein turnover data, leading to 75% gain in discovered disease gene candidates in six genetic strains of mice [272]. Analysis of multiomics data (target predictions, pathway analyses) needs excessive bioinformatic expertise. However, user-friendly software is rapidly emerging, but their diversity in mathematical models and source data do not yet allow accurate comparison of different results.

## Proof-of-concept clinical phase I/II trials with surrogate endpoints

Although phase I/II studies defining dosing, timing and safety of cardioprotective interventions may appear to be a straightforward task, the field of cardioprotection by not only mechanical but also pharmacological interventions is characterized by a surprising lack of adequate phase I/II studies. While animal studies allow the establishment of an optimal algorithm of cardioprotective treatments by studying the influence of number and duration of cycles, influence of effector organ mass and timing of remote ischemic preconditioning on infarct size [228, 393] and such studies are also feasible in larger animals, they are more challenging in the clinical setting, because dose–response studies using infarct size or myocardial salvage require acute settings and a large number of patients, regardless of whether the endpoint is determined from a biochemical injury marker or an imaging modality.

### Surrogate endpoints: peripheral circulation

Hence, the translation to human proof-of-concept studies frequently requires use of surrogate endpoints. Studies should be initiated in healthy volunteers and subsequently proceed to basal conditions [343] or an acute ischemia/reperfusion equivalent that may be precipitated by exercise, pharmacologically induced myocardial ischemia, or elective percutaneous coronary intervention (PCI) in patients with stable coronary artery disease [213]. During elective PCI, measurements of biochemical myocardial injury markers can be used, whereas studies in healthy volunteers and patients with stable coronary artery disease not undergoing coronary interventions require alternative endpoints. Because ischemia/reperfusion induces endothelial dysfunction, the most frequently used surrogate endpoint is endothelial ischemia/reperfusion injury of the human forearm induced by 20 min of upper limb ischemia by inflation of a blood pressure cuff to 200 mm Hg followed by reperfusion [248]. Forearm blood flow in response to acetylcholine at baseline and 15 min after reperfusion can be assessed by venous plethysmography [248]. Alternatively, high-resolution ultrasound can be used to measure the diameter of the superficial brachial artery at rest, during reactive hyperemia (with increased flow causing endothelium-dependent dilatation), and after sublingual glyceryl trinitrate (causing endothelium-independent dilatation) [67]. The latter model requires measurements on at least two occasions before and after any intervention, to optimize the probability of showing a significant effect from any potentially beneficial therapy [398].

The finger hyperemia index correlates with values of flow-mediated dilatation by means of brachial artery ultrasound scanning [264]. This modality measures fingertip pulse-volume amplitude to calculate a reactive hyperemia index, assessed as a measure of peripheral microvascular endothelial function. Finger reactive hyperemia is evaluated noninvasively in the index finger of the left hand of participants using, e.g. the EndoPAT 2000 device (Itamar Medical, Caesarea, Israel). A beat-to-beat plethysmographic recording of the arterial pulse wave is obtained over 5 min of rest. Brachial arterial occlusion is then induced by inflating an upper arm cuff to suprasystolic pressure (60 mmHg above systolic pressure) for 5 min and released to elicit reactive hyperemia. Pulse amplitude responses to hyperemia are calculated from the hyperemic fingertip as the ratio of the postdeflation pulse amplitude to the baseline pulse amplitude and adjusted for the corresponding ratio in the right control hand and expressed as finger reactive hyperemia [266]. Although these studies bring important insights into our understanding of the pathophysiology of ischemia/reperfusion injury, one must be aware that they may not reflect or predict the actual impact of a given putatively “protective” intervention to be tested on the “ultimate” insult, i.e. irreversible myocardial injury following a prolonged coronary artery occlusion [207]. None of the above-mentioned variable/surrogate endpoints is indeed predictive of clinical outcomes in patients with acute myocardial infarction, i.e. truly indicative of “cardioprotection” (which is primarily infarct size reduction).

### Surrogate endpoints: coronary circulation

The coronary circulation is a major target for cardioprotective strategies [198]. Blood flow velocity in the LAD can be assessed non-invasively by transthoracic Doppler echocardiography before and after a cardioprotective intervention [461]. Doppler signals of coronary blood flow should be recorded at baseline and 1, 3, 6 and 9 min after the cardioprotective intervention in three cardiac cycles. The ECG must be continuously monitored to register changes in heart rate. Outcome variables include the mean coronary blood flow velocity, peak diastolic velocity, mean diastolic velocity, peak systolic velocity and mean systolic velocity. Following a baseline blood flow velocity measurement, intravenous adenosine infusion of 140 μg/kg/min for 90 s allows coronary blood flow reserve quantification and hence assessment of microvascular function. However, caution should be exerted about repetitive measurements with short intervals because adenosine per se exerts cardioprotective effects [281], so that measurements on separate days are advisable to avoid interference between a cardioprotective treatment and adenosine.

The epicardial endothelial and endothelium-independent vascular function [87] and the impact of coronary collaterals [20] can be studied in patients with stable coronary artery disease undergoing a coronary intervention. To avoid any interference, the discontinuation of the patient’s long-acting antianginal medication is recommended 48 h prior to the investigation. Endothelium-dependent vasodilator function is assessed preferentially both in a stenotic target and a non-stenotic non-target coronary artery during incremental intracoronary acetylcholine doses at 1, 3, 10, and 30 μg/min for 2 min at each dose. Non-endothelium-dependent vasodilator function is measured subsequently using intracoronary nitroglycerine, 0.40 mg. Coronary luminal diameter is assessed by quantitative coronary angiography and coronary blood flow velocity measured by Doppler. The influence of any cardioprotective treatment on coronary blood flow reserve can be determined using intracoronary adenosine 40 and 80 μg in the right and left coronary artery, respectively. As with non-invasive studies, venous blood sampling before and after the cardioprotective procedure or sham should be added, as circulating markers yield additional information on inflammatory, (e.g. myeloperoxidase, interleukin-6) coagulatory, (von Willebrand factor, asymmetric dimethylarginine) and fibrinolytic (tissue plasminogen activator) function. As with non-invasive studies, results obtained by repetitive measurements must be interpreted in the light of the fact that adenosine per se exerts cardioprotective effects [86].

Invasive results relate to the epicardial coronary artery [208] and they only reflect the coronary microcirculation when coronary flow reserve is quantitated using adenosine (intravenous infusion of 140 μg/kg/min for 90 s). Positron emission tomography allows quantification of myocardial blood flow before and after a cardioprotective intervention in patients with stable angina pectoris, and positron emission tomography imaging enables regional visualization of non-ischemic and reversibly and irreversibly ischemic myocardial areas so that therapeutic effects on the “true” cardioprotection target (namely infarct size) can be assessed separately in these regions [343].

## ST segment elevation myocardial infarction

### Patients

STEMI patients recruited for cardioprotection studies must meet the absolute indication for revascularization, i.e. chest pain, < 12 h since symptom onset, ST-segment elevation in at least two contiguous leads (≥ 2.5 mm in men < 40 years, ≥ 2 mm in men ≥ 40 years, or ≥ 1.5 mm in women in leads *V*_2_–*V*_3_ and/or ≥ 1 mm in the other leads) in the first ECG.

The recruitment of patients depends on the conditions, and patient selection may vary. Selected patients, who are most likely to benefit from a novel cardioprotective strategy [57], can be identified prior to admission, when a large anterior infarction presents on the ECG or at the catheterization laboratory or when the LAD is occluded proximally. An unselected group to determine efficacy in dependence of infarct location, vessel patency, collateral status, etc. may be preferred to clarify the influence of these confounders. Baseline characteristics should include the variables specified in Table 4.

**Table 4:** Baseline characteristics for STEMI patients included in cardioprotection trials

Age
Gender
Risk factors
Smoking
Comorbidities
Dyslipidemia
Hypertension
Diabetes mellitus
History of coronary artery disease
Previous MI
Preinfarction angina within 1 week before the index event
Infarct-related artery
Left anterior descending artery
Circumflex artery
Right coronary artery
Not identifiable
Number of other vessels with clinically significant disease
Symptom-to-balloon time
TIMI flow grade at admission
0/I/II/III
Stenting of culprit lesion by pPCI
TIMI flow grade after procedure
0/I/II/III
History of medication
Beta-blockers
Statins
ACE-inhibitors
AT-blockers
Calcium channel blockers
Long acting nitrates
Metformin
Insulin
Other antidiabetic drugs
Drugs given at time of pPCI
Heparin
Aspirin
P2Y_12_-inhibitors

### Outcome measures

In proof-of-concept clinical studies, it may be sufficient to measure serum cardiac enzymes, i.e. CK-muscle/brain (CK-MB) and troponin-T (TnT) or TnI. However, a more robust surrogate clinical endpoint is infarct size (measured by myocardial nuclear scanning or CMR). Ultimately, clinical outcome measures such as (cardiac) mortality and/or hospitalization for heart failure must be determined.

#### Surrogate endpoints

##### Imaging data

Two experienced nuclear cardiology investigators blinded to treatment assignment and clinical data must analyze the data independently. Images can be analyzed with a commercially available automatic program. In case of failure of the automatic quantification algorithm, methods to mask extracardiac activity or to define the valve plane and apex of the left ventricle, or both, can be used. If the difference in infarct size between the readers exceeds 3%, a consensus reading is recommended from the two or additional readers.

##### Quantification of area at risk

CMR: This technique has been used extensively in recent years to quantify the areas of edema as a surrogate to area at risk. It cannot be recommended to use CMR to retrospectively quantify area at risk, for the reasons given in the section "Quantitation of infarct size by morphology, biomarkers and imaging" [210].

SPECT: Before reperfusion therapy is started, 700 MBq (± 10%) ^99^Tc-sestamibi is administered intravenously; within 8 h of injection of the radionuclide, SPECT must be performed with a high-resolution parallel-hole collimator dual headed rotating gamma camera, see section "Quantitation of infarct size by morphology, biomarkers and imaging".

##### Quantification of infarct size

CMR: LGE–CMR imaging must be performed to quantify infarct size 10–15 min after intravenous administration of 0.20 mmol of gadopentetate dimeglumine contrast agent per kg of body weight, see section "Quantitation of infarct size by morphology, biomarkers and imaging".

SPECT: infarct size is quantified using the same protocol as for the area at risk quantification 30 days after the STEMI event.

##### Biomarkers of myocardial injury

Extending section "Quantitation of infarct size by morphology, biomarkers and imaging", CK or preferably high-sensitive troponins (TnT or TnI) are measured at 0, 6, 12, 24, 48 and 72 h following the revascularization procedure for establishment of a 72 h area under the curve. Attention must be paid to use a unique kit for one assessment of these biomarkers.

##### Clinical endpoints

Cardiac mortality and readmission for heart failure at 30 days and 1 year are the most important clinical outcome endpoints. All-cause mortality at 30 days and 1 year should also be recorded, as well as MACCE at 30 days and 1 year.

### The cardioprotective intervention

More than a few cardioprotective studies have been forced rapidly from inadequate preclinical experiments to clinical trials that may have been premature [56, 181, 184, 196]. To overcome the obstacles in translating new cardioprotective strategies discovered in the laboratory into the clinical setting for patient benefit, the strategy should be founded on solid experimental, preclinical, and translational data, including in vitro models in established animal models of ischemia/reperfusion injury and including human myocardial tissue models of simulated ischemia/reperfusion injury as described above. The preclinical translation studies should not only include experiments, which involve a variety of confounding factors that may modify the efficacy of the cardioprotective strategy, but also reflect the polypharmaceutical environments in which the cardioprotective strategy will be employed in clinical trials, e.g. on the current background of current medical therapy for STEMI patients including opiates and P2Y_12_ inhibitors which share cardioprotective signaling with ischemic conditioning and induce cardioprotection per se, as discussed in section "Larger mammal hearts in situ" [85, 450]. Dose–response studies are not only required in pharmacological studies but also in studies of mechanical conditioning such as remote ischemic conditioning. Such studies may be challenging because a direct cardioprotective effect in humans relies on surrogate markers, most frequently endothelial ischemia/reperfusion injury, and available methods are not sufficiently sensitive to measure minor differences between doses. A hybrid method using different cycle numbers and durations of ischemia/reperfusion in humans with subsequent transfer of cardioprotective plasma samples to a mouse, rat or rabbit heart exposed to ischemia/reperfusion injury in a Langendorff mode may be recommended for establishing human dose–response studies. Of note, the conditions of reperfusion by PCI may also influence the efficacy of the supposedly protective intervention, including use of direct stenting or “pre-dilatation” or “post-dilatation” that may per se trigger inadvertent postconditioning and bias the final analysis [196].

## Cardiac surgery

### Patients

Overall, outcome following cardiac surgery is favorable [6]. The majority of complications relate to mechanical obstacles or bleeding that may not be significantly modified by cardioprotective strategies. Nevertheless, perioperative myocardial damage attributed to ischemia/reperfusion injury has been associated with increased perioperative morbidity and mortality [111, 422]. The risk of perioperative myocardial damage is increased in patients with a EuroSCORE (European System for Cardiac Operative Risk Evaluation) > 5, and cardioprotective efficacy is thought to be enhanced in patients with left ventricular hypertrophy or impaired left ventricular systolic function, patients undergoing three-vessel coronary artery bypass graft with/without valve surgery or redo coronary artery bypass graft surgery and patients with diabetes mellitus. Hence, the investigation of a new cardioprotective strategy should focus on such patients. Baseline characteristics should include the variables specified for STEMI patients, except those specifically relating to culprit lesion treatment in STEMI patients.

### Outcome measures

In proof-of-concept clinical studies, perioperative release of cardiac ischemic biomarkers, such as CK, preferably as the myocardial fraction CK-MB, TnT or TnI is the only way to assess the efficacy of the novel cardioprotective strategy on myocardial injury [186, 420, 421]. One must, however, be aware that cardiac surgery including myocardial incision or coronary clamping (e.g. coronary bypass) will per se cause cardiac enzyme release, hence alter interpretation of the data. Other clinical endpoints include: inotrope score, duration of mechanical ventilation, length of intensive care unit and hospital stay, left ventricular ejection fraction, acute kidney injury, cognitive function, cardiovascular mortality, and hospitalization for heart failure at 30 days and 1 year. Although there is no direct link between perioperative cardioprotection and the need for coronary revascularization, and although heart failure is not included, a frequently used clinical endpoint is a composite endpoint of the rate of MACCE (death from cardiovascular causes, nonfatal myocardial infarction, coronary revascularization, or stroke), assessed within 12 months after randomization [182]. An independent event validation committee must validate all primary events.

### Anesthesia

Inhaled anesthetic agents such as isoflurane and sevoflurane, the recommended first choices in patients at risk of myocardial ischemia [455], and intravenously administered propofol have all been reported to confer cardioprotection during coronary artery bypass graft surgery in preclinical and clinical studies [410]. It is important that any novel cardioprotective strategy is shown to be effective in the presence of routine medical therapy. Even so, it is necessary to take interference with cardioprotective strategies into consideration. Propofol seems no more cardioprotective than isoflurane per se, but in contrast to isoflurane and sevoflurane, propofol specifically abrogates the protection by remote ischemic conditioning in patients undergoing elective coronary artery bypass graft surgery [32, 260, 261, 454], and seems to be a common denominator of all studies that failed to see protection with remote ischemic conditioning [204, 302]. So, propofol must be avoided specifically in studies investigating the efficacy of remote ischemic conditioning.

### Concomitant medication

While it may be possible to standardize the anesthetic regimen, concomitant medication in clinical cardioprotection studies may vary significantly. Providing the study is adequately powered and properly randomized, confounding factors should be distributed equally between the study intervention and the standard treatment groups. When it is difficult to perform a large clinical study without standardization of concurrent medical therapy, stratification of the study with respect to confounding factors must be considered.

## Abbreviations

ADP: Adenosine diphosphate
ANOVA: Analysis of variance
ATP: Adenosine triphosphate
CK: Creatine kinase
CK-MB: Creatine kinase-muscle/brain
CMR: Cardiac magnetic resonance imaging
CsA: Cyclosporine A
ECG: Electrocardiogram
EGTA: Ethylene glycol-bis(β-aminoethyl ether)-*N*,*N*,*N*’,*N*’-tetraacetic acid
FCCP: Carbonyl cyanide-4-(trifluoromethoxy)phenylhydrazone
FCS: Fetal calf serum
HEPES: 4-(2-hydroxyethyl)-1-piperazineethanesulfonic acid
ICH: International conference on harmonization
IFM: Interfibrillar mitochondria
JC-1: Tetraethyl-benzimidazolyl-carbocyanine iodide
KHB: Krebs–Henseleit buffer
LDH: Lactate dehydrogenase
LGE: Late gadolinium enhancement
LOCF: Last observation carried forward
MACCE: Major adverse cardiac and cerebral events
MOPS: 3-(*N*-morpholino)propanesulfonic acid
mPTP: Mitochondrial permeability transition pore
MTT: (3-(4,5-dimethylthiazol-2-yl)-2,5-diphenyltetrazolium bromide) tetrazolium
PPCI: Primary percutaneous coronary intervention
RNA: Ribonucleic acid
ROS: Reactive oxygen species
SPECT: Single-photon emission computed tomography
SSM: Subsarcolemmal mitochondria
STEMI: ST segment elevation myocardial infarction
T2w-CMR: T2-weighted cardiac magnetic resonance imaging
TMRE: Ethyl ester of tetramethylrhodamine
TMRM: Methyl ester of tetramethylrhodamine
TTC: 2,3,5-triphenyl tetrazolium chloride

## Acronyms

ARRIVE: Animal research: reporting of in vivo experiments
CAESAR: Consortium for preclinical assessment of cardioprotective therapies
CONDI-2: Effect of remote ischemic conditioning on clinical outcomes in STEMI patients undergoing pPCI
ERIC-PPCI: Effect of remote ischemic conditioning on clinical outcomes in ST-segment elevation myocardial infarction patients undergoing primary percutaneous coronary intervention
EuroSCORE: European system for cardiac operative risk evaluation
TIMI: Thrombolysis in myocardial infarction
